# Wildlife Population Dynamics in Human-Dominated Landscapes under Community-Based Conservation: The Example of Nakuru Wildlife Conservancy, Kenya

**DOI:** 10.1371/journal.pone.0169730

**Published:** 2017-01-19

**Authors:** Joseph O. Ogutu, Bernard Kuloba, Hans-Peter Piepho, Erustus Kanga

**Affiliations:** 1 Biostatistics Unit, Institute for Crop Science, University of Hohenheim, Stuttgart, Germany; 2 Kenya Wildlife Service (KWS), Nairobi, Kenya; Centre for Cellular and Molecular Biology, INDIA

## Abstract

Wildlife conservation is facing numerous and mounting challenges on private and communal lands in Africa, including in Kenya. We analyze the population dynamics of 44 common wildlife species in relation to rainfall variation in the Nakuru Wildlife Conservancy (NWC), located in the Nakuru-Naivasha region of Kenya, based on ground total counts carried out twice each year from March 1996 to May 2015. Rainfall in the region was quasi-periodic with cycle periods dependent on the rainfall component and varying from 2.8 years for the dry season to 10.9 years for the wet season. These oscillations are associated with frequent severe droughts and food scarcity for herbivores. The trends for the 44 wildlife species showed five general patterns during 1996–2015. 1) Steinbuck, bushbuck, hartebeest and greater kudu numbers declined persistently and significantly throughout 1996–2015 and thus merit the greatest conservation attention. 2) Klipspringer, mongoose, oribi, porcupine, cheetah, leopard, ostrich and Sykes monkey numbers also decreased noticeably but not significantly between 1996 and 2015. 3) Dik dik, eland, African hare, Jackal, duiker, hippo and Thomson’s gazelle numbers first increased and then declined between 1996 and 2015 but only significantly for duiker and hippo. 4) Aardvark, serval cat, colobus monkey, bat-eared fox, reedbuck, hyena and baboon numbers first declined and then increased but only the increases in reedbuck and baboon numbers were significant. 5) Grant’s gazelle, Grevy’s zebra, lion, spring hare, Burchell’s zebra, bushpig, white rhino, rock hyrax, topi, oryx, vervet monkey, guinea fowl, giraffe, and wildebeest numbers increased consistently between 1996 and 2015. The increase was significant only for rock hyrax, topi, vervet monkey, guinea fowl, giraffe and wildebeest. 6) Impala, buffalo, warthog, and waterbuck, numbers increased significantly and then seemed to level off between 1996 and 2015. The aggregate biomass of primates and carnivores increased overall whereas that of herbivores first increased from 1996 to 2006 and then levelled off thereafter. Aggregate herbivore biomass increased linearly with increasing cumulative wet season rainfall. The densities of the 30 most abundant species were either strongly positively or negatively correlated with cumulative past rainfall, most commonly with the early wet season component. The collaborative wildlife conservation and management initiatives undertaken on the mosaic of private, communal and public lands were thus associated with increase or no decrease in numbers of 32 and decrease in numbers of 12 of the 44 species. Despite the decline by some species, effective community-based conservation is central to the future of wildlife in the NWC and other rangelands of Kenya and beyond and is crucially dependent on the good will, effective engagement and collective action of local communities, working in partnerships with various organizations, which, in NWC, operated under the umbrella of the Nakuru Wildlife Forum.

## Introduction

Communally and privately protected pastoral areas are critically important for wildlife conservation in Kenya and jointly support about 65–70% of the country’s wildlife population [1,2]. The Nakuru Wildlife Conservancy (NWC) located in the Nakuru-Naivasha region of Kenya supports both livestock production and wildlife conservation on publically, communally and privately owned conservation areas. Early European explorers and hunters documented several wildlife species in the Nakuru-Naivasha region. [3] observed several waterbuck (*Kobus ellypsiprymnus)* on the Cresent Island in Naivasha in 1902. Waterbuck used to regularly swim, usually at night, to feed on the Cresent Island in Lake Naivasha [4]. By 1903 the vicinity of Lakes Naivasha, Nakuru and Elementaita supported one of the richest wildlife assemblages in East Africa. The climate was mild and the grass never became extremely tall. Thomson’s (*Gazella thomsoni*) and Grant’s (*Gazella granti*) gazelles were particularly numerous in the dry country between Lakes Nakuru and Elementaita before 1914 [4–6]. Immense numbers of both gazelles congregated here in January [4,6,7]. There were more Thomson’s gazelles near Nakuru than anywhere else in East Africa during the seasonal migration at the end of the rains between north of Nakuru and areas near Lakes Nakuru and Elementaita [4]. These migrations also involved zebra (*Equus quagga*) [4]. Elephants (*Loxodonta africana*) also used to move frequently between the Nakuru area and the Laikipia plains [8]. Zebra also used to trekk from time to time from Elementaita to Solai, Njoro and Molo Rivers. They first walked to Nakuru where they split up, one half going to the south of Menengai and then on to Njoro and Molo, the other half going to Solai and Hannington District (Baringo County) [6]. All these movements of gazelles, zebra and elephants no longer occur in the region except for a few elephants that still occasionally move from Baringo County to salt licks on the shores of Lake Solai. Coke’s hartebeest (*A*. *b*. *cokii*) but not Nakuru hartebeest (*A*. *b*. *jacksoni x cokii*) occurred in Naivasha [6,9]. Nakuru hartebeest [3] was first reduced by heavy shooting in the 1920s and 1940s before being exterminated from the region [6,9]. Many species of other large mammals have similarly been killed, exterminated or driven out of the area, including elephants, lions (*Panthera leo*) and the bearded vulture or lammergeyer (*Gypaetus barbatus*), which recently became extinct in the region. Some lions still move between the NWC and the surrounding regions. Spotted hyenas (*Crocuta crocuta*), though also persecuted in the past, have managed to maintain a small population in the NWC.

The human population in the Nakuru County (7510 km^2^) increased by more than 300% from 1969 to 1187039 in 1999 and by a further 74% to 1603325 by 2009. Average human population density was 41/ km^2^ in 1969, 158/ km^2^ in 1999 and 214 people/ km^2^ in 2009, with higher densities concentrated around the urban centers and areas of intensive agriculture. The human population growth is associated with a dramatic expansion of the area covered by the three major towns (Nakuru, Naivasha and Gilgil), increased development and human presence in areas adjoining the developed areas. The increasing human population is also associated with increasing disturbance or harassment of wildlife by people, dogs, lights, noise, traffic and other factors. The increase in human density within the conservancy plays a significant role in discouraging wildlife from using the area to a greater degree [10]; a problem more likely to worsen rather than improve with time as the area continues to grow [11].

The principal land uses in the NWC and its immediate surroundings include subsistence and commercial agriculture (mainly flower farming for export), developed areas, pastoralism, cattle ranching and wildlife tourism in three national parks (Hell’s Gate, Mt. Longonot and Lake Nakuru), private conservation areas and forest reserves. Developed areas are dominated by built structures, including homes, villages, storage sheds, urban areas, flower farms (greenhouses), roads, and other infrastructures. These areas characteristically have high human densities and low wildlife densities. Expansion of the developed areas has been associated with marked increases in the coverages of bare soil and bushland but reduced grassland cover and patch size [11]. The land outside of the parks is facing mounting pressures from the growing human population and aspirations for conversion to human uses. Notably, the area has experienced phenomenal growth of the commercial flower farm industry since the first flower farm started in the early 1980s. Correspondingly, there has been a large amount of growth in developed areas due to the infrastructure required to support the flower industry and the workers employed in it. The large growth in the human population and developed areas, combined with the expansion of small scale agriculture, are having deleterious effects on wildlife in the Conservancy. These effects are likely to intensify as habitat loss, fragmentation and degradation become more widespread and pronounced. Overgrazing by livestock and poor land management practices, implicated in grassland degradation elsewhere [11–15], are likely responsible for the increase of bare soil and bush encroachment in the Conservancy as well.

The environmental and political pressures acting on the Conservancy, including intensification of land use and rising population pressures, accelerate habitat loss, fragmentation and degradation [16]. Thus, the Conservancy and Lake Naivasha are threatened by several processes, including the following [17–21]: (*i)* Expansion of the intensively irrigated floriculture and horticulture, (*ii*) rapid and unplanned urban, slum and other developments, including perimeter fencing of some properties, (*iii*) excessive water abstraction from Lake Naivasha, rivers and ground water for irrigation and power generation, (*iv*) extensive destruction of vegetation through expansion of cultivation and settlements, fires, felling of trees for building poles, firewood and charcoal burning, (*v*) overgrazing by livestock, (*vi*) water pollution caused by agrochemicals used by the horticultural farms and smallholders in the upper catchment, (*vii*) siltation by river sediments and rising water turbidity, (*viii*) invasive plant and animal species within the lake drainage basin, (*ix*) increased intensity of human use, including fishing, tourism and over use of wetland plants and (*x*) illegal hunting, snaring and poisoning of wildlife. The catchment may also be threatened by increasing extractions of surface and underground water upstream, increasing erosion, agrochemicals, deforestation and increasing industry and transport. There is also pollution (domestic and industrial effluents) from the rapidly expanding Naivasha Town and inter basin water transfer to areas to the north with less water supply [22]. The competing land uses further contribute to human-wildlife conflicts. Conflicts arise because of competition for space, crop destruction, livestock depredation and threats to human life by wildlife [23]. Poaching, widespread poverty around the conservancy, large-scale snaring of wildlife for meat and skins, fires, competition between livestock and wildlife for space and food, land subdivision, fencing and accidents involving wildlife crossing the Nakuru-Nairobi highway further threaten wildlife in the region [24].

Nevertheless, Nakuru Wildlife Conservancy still sustains a diverse wildlife community. We consider 44 common species censused in this study (S1 Table), including zebra, eland (*Tragelaphus oryx*), waterbuck, hartebeest (*Alcelaphus buselaphus Cokei*), Thomson’s and Grant’s gazelles, impala (*Aepyceros melampus*), warthog (*Phacochoerus africanus*), African hare (*Lepus capensis*), helmeted guinea fowl (*Numida meleagris*), steinbuck (*Raphicerus campestris*) and dik dik (*Madoqua kirkii*). Other species recorded in the Conservancy during the censuses but that were too rare to model their trends, include gerenuk (*Litocranius walleri*), zorilla (*Ictonyx striatus*), secretary bird (*Sagittarius serpentarius*) and Kori bustard (*Ardeotis kori*) (S1 Table and S3 Data). The mammalian species inhabiting the riparian land include the African buffalo (*Syncerus caffer*), colobus monkey (*Colobus guereza*) and waterbuck.

No earlier studies seem to have examined the population dynamics of wildlife in the NWC. [11] analyzed trends in the cheetah (*Acinoyx jubatus*) population in relation to their prey species in the NWC between 1996 and 2003. [23] examined trends in reported human-wildlife conflict incidences in the NWC between 1993 and 2007. [2, 25–27] analyzed wildlife population trends in Lake Nakuru National Park (LNNP), adjoining the NWC to the northwest and fully fenced since 1976, based on bimonthly counts covering the periods 1970–1973, 1970–1992, 1970–2002 and 1970–2011, respectively.

Here, we briefly review the historical population status of wildlife in the NWC in the early years when land use change was limited and human population size was small and analyze wildlife population dynamics in the NWC from 1996 to 2015. We infer the importance of the communal and private conservancies, sanctuaries and ranches, as a strategy for countering rapid wildlife declines in pastoral lands of Kenya [2,28–31], using the example of the Nakuru Wildlife Conservancy. We discuss why communal and private conservancies are important for conservation in Kenya, how community or private engagement is achieved and lessons learnt concerning how communities should be involved and when their involvement is likely to work, also using the example of the NWC. We also assess threats to wildlife conservation in the Nakuru-Nakuru region.

(H_1_) We expected to find persistent declines in wildlife numbers due to habitat loss, fragmentation and degradation associated with the land use changes, growing human population and other pressures in the region. In particular, (H_2_) we expected large-bodied animals to be more adversely affected by the land transformations and hence to decline more than small-bodied animals because of their larger forage and range requirements and greater likelihood of conflicts with humans. (H_3_) If culling of large herbivores from 1992 suppressed their population growth in NWC before it was outlawed in 2002, then we would expect to see an increase in their population size after but not before 2002.

In African savanna environments, rainfall is the key climatic component governing vegetation production and quality [32–36]. Rainfall thus controls the quantity and quality of forage for herbivores and is significantly correlated with the Normalized Difference Vegetation Index (NDVI) that measures grass greenness, with the correlation being as high as 0.89 [37,38]. As a result, there are strong positive correlations between rainfall and animal biomass [39–41], abundance [38, 42] and population dynamics [43,44] in African savannas. (H_4_) Accordingly, we also expected the aggregate biomass of the wild herbivores and the densities of the individual herbivore species to increase with increasing cumulative past rainfall, indexing changing food availability and habitat suitability for herbivores [39–41].

## Materials and Methods

### Study area

**Fig 1:**
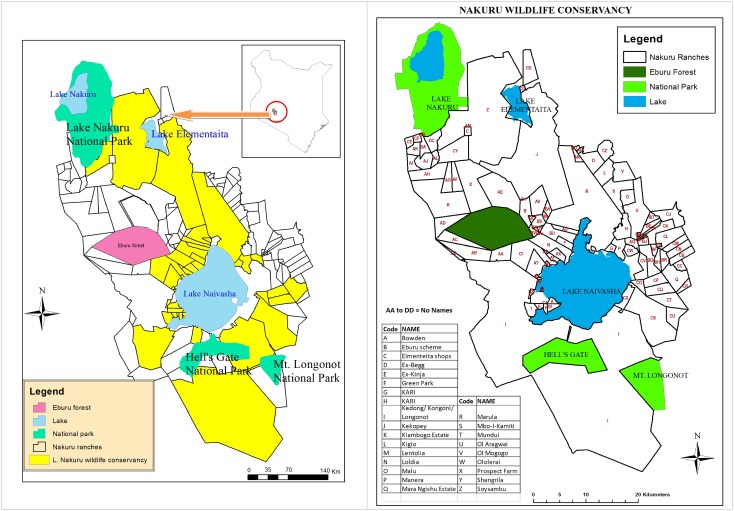
**a) A map showing the properties constituting the Nakuru Wildlife Conservancy and the neighbouring non-conservancy properties in the Naivasha-Nakuru region of Kenya (Left panel)**. b) **A map showing the areas covered by the total counts in the Nakuru Wildlife Conservancy and the neighbouring non-conservancy properties in the Naivasha-Nakuru region of Kenya (right panel)**. Areas covered by the total counts are labeled with alphabetical letters.

The Nakuru Wildlife Conservancy is located in the Nakuru County of Kenya, about 100 km northwest of Nairobi (latitude -0° 27′ 54″ and longitude 36° 12′ 4″, Fig 1a). The Conservancy, covering an area of about 1417 km^2^ currently consists of about 33 different properties (S2 Table) and is managed by the Nakuru Wildlife Forum (NWF), a grouping of communal, private and public land owners and managers who work together to make landscape-level management decisions for the benefit of the Conservancy. Several small- to large-scale wildlife and livestock ranches, wildlife sanctuaries, national parks and forest reserves are part of the Conservancy. The parks and sanctuaries include the Hell’s Gate National Park (68.25 km^2^, but the counts cover about 65 km^2^ because part of the park is fenced off and used for Geothermal Power Generation and hence has no animals), that adjoins Lake Naivasha to the south and has an access corridor to the lake, and Mt. Longonot National Park (52 km^2^, but the counts cover an additional 29 km^2^ of the adjoining Kedong Ranch and a private Game Sanctuary found on the Cresent Island on the Lake). The fourth largest city in Kenya, Nakuru, is located on the northern edge of the conservancy. Naivasha and Gilgil are the two other major towns neighbouring the Conservancy.

Lake Naivasha (0° 42′-50′ S, 36° 16′-26′ E), located in the study area, is an Important Bird Area, designated as a Ramsar site in April 1995. The Lake occurs at an altitude of 1890 m. Lake Naivasha (*ca*. 150 km^2^) is the largest water body in a system of four lakes, the other three of which are Oloidien (5.5 km^2^), Cresent Lake (2.1 km^2^), and the Crater Lake or Sonachi (0.6 km^2^). Lake Naivasha is an important national water resource because it is the only fresh water lake on the floor of the Eastern Rift Valley in Kenya. The Lake is generally shallow (average depth: 4–6 m) and has a subsurface outflow. The Lake depth has varied by as much as 12 m during the last century due to strong evaporation (ca. 1720 mm /year) and highly variable river inflow associated with interannual rainfall fluctuations [17, 45–47]. The Lake is characterized by submerged macrophytes, notably *Potamogeton spp*., and floating rafts of the exotic Water Hyacinth *Eichhornia crassipes* [48]. Most of the shore is fringed by extensive papyrus (*Cyperus papyrus*).

The Lake and its immediate environs support over 400 bird species, 90 of which are aquatic or semi-aquatic. The Lake serves as an important stopover point for many migratory birds. Besides birds, the lake supports numerous other animal species including hippos (*Hippopotamus amphibius*). The most common fish in Lake Naivasha is *Tilapia spp*, including *T*. *nigra* (introduced in 1925), *T*. *leucosticta* (introduced in 1954) and *T*. *zillii* (introduced in 1965). Another common fish is the large mouthed Black Bass *Micropterus salmoides* (introduced in 1929, 1940 and 1951). Additional fish species found in the Lake are *Oreochromis leucostictus*, *Barbus amphigramma* and *Lebistes reticulata*.

The Lake catchment area covers 3200 km^2^. The main rivers draining into the Lake are the perennial Malewa (1750 km^2^) and Gilgil (420 km^2^) and the ephemeral Karati (70 km^2^) River catchments. Malewa and Gilgil Rivers drain the northern part of the Lake whereas Karati drains the eastern part. Several small basins with a combined size of about 1000 km^2^ drain the southern and western parts of the Lake Basin. The upper water catchments comprise five upland forests, namely the Mau, Eburru, Kipipiri, Kinangop and the Aberdares [21,49–51].

The dominant habitat types in the conservancy include closed forests, open forests, grassland, bushland, water bodies and baldlands. Forests of yellow fever acacia (*Acacia xanthophloea*) are common around the lakes, rivers, in areas with high ground water and black cotton soil and dominate closed forests with dense understory in the conservancy. Other tree types common in the closed forests are blue gum (*Eucalyptus globulus*), pine (*Pinus sp*.), euphorbia (e.g., *Euphorbia bussei*, *E*. *candelabrum*) and deciduous mixed hardwoods. Open forests have an understory dominated by grasses and the yellow fever tree. Open forests also typically fringe lakes, rivers and other waterways. Grassland areas are dominated by grasses, short herbaceous plants, dead biomass and patches of bare soil. Bushland areas are dominated by shrubs, bushes and short trees interspersed with grasses and patches of bare soil. The most common species found in the bushland areas include leleshwa (*Tarchonanthus camphoratus*), acacia (*Acacia sp*.), croton (*Croton sp*.), grewia (*Grewia sp*.) and rhus (*Rhus sp*.). Agricultural areas consist of subsistence and commercial (mainly flower farming for export) farms, homes, storage sheds and other buildings. The principal water bodies include the lakes, rivers and streams. Badlands consist of thick bush and sparse grasses and occur on old lava flows and other types of rocky outcrops. They are dominated by bushes and herbaceous species, including *Aloe sp*., *Croton sp*., *Euphorbia sp*., *Acacia sp*., *Grewia sp*. and *Rhus sp*. Grass cover is sparse on baldlands. Mudflats around the lakes, degraded lands and cleared patches around urban areas are dominated by bare soil [11].

## Methods

### Rainfall measurements

**Fig 2:**
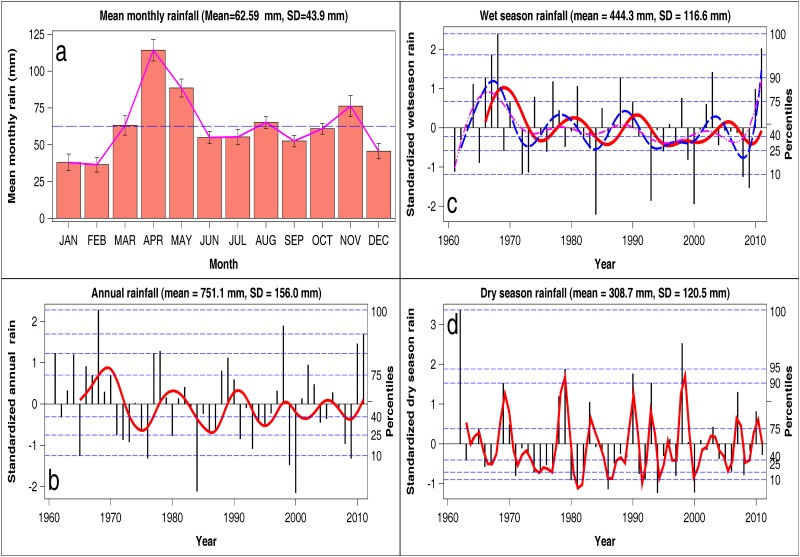
a) The distribution of the total monthly rainfall across months in Nakuru Wildlife conservancy averaged over 1967 and 2011 and the standardized deviates for the b) annual, c) wet season and d) dry season rainfall components. The vertical needles in panels **b**-**d** are the observed standardized deviates, the thick solid lines are the 4-year moving averages for the annual and wet season rainfall components and the 3-year moving average for the dry season rainfall component. The dashed horizontal lines are the percentiles of the standardized deviates for each rainfall component. The two dashed curves in the panel for the wet season rainfall are the secondary cycles with 3.5 and 2.2-year periods.

The study area is semi-arid. Rainfall increases along a south-north gradient and averages 652 mm (range 336–942 mm) in the south on the shores of Lake Naivasha and 869 mm (range 363–1146 mm) in Lake Nakuru National Park and its immediate environs in the north. Rainfall falls during the long (March-June) and short (September-November) rainy seasons. The driest months span December-February and July. Total monthly rainfall was obtained for seven recording stations within the Lake Naivasha catchment basin (Fig 2). The mean annual temperature is around 26°C and the maximum temperature averages 30°C. The coldest months are April and July, and have a mean annual temperature of 16–17°C. The hottest months, January-March, have a mean temperature of 28–30°C. The evapotranspiration around the lake far exceeds precipitation.

#### Animal counts

Animals were counted using attempted total counts. The attempted total count method is widely used for counting animals in African savannas. For example, attempted total counts from a vehicle are used to count animals every two months in the Lake Nakuru National Park adjoining the Nakuru Wildlife Conservancy since 1970 [25–27,52] and in Nairobi National Park of Kenya since 1960 [6,30,53–57]. Attempted aerial total counts have also been used to count animals in the Kruger National Park in South Africa from 1977 to 1997 [44,58,59], Masai Mara National Reserve of Kenya since 1962 [60] and the Serengeti National Park since 1958 [61]

Even though it cannot provide estimates of the accuracy of population size estimates or detectability of animals, the attempted total count method is able to provide estimates for even rare animal species, which are of great conservation concern but which are hard to count using sampling techniques. For example, after the total counting procedure was replaced with aerial distance sampling in the Kruger National Park in 1998, it became difficult to obtain reliable estimates of trends for several species, such as warthog, wildebeest and waterbuck, even with a coverage intensity exceeding 22% of the park area [62]. Similarly, in the Nakuru Wildlife Conservancy, [11] used distance sampling to count potential cheetah prey species along 13 foot transects, covering a total of 55.3 km of the Conservancy. As expected, sightings of rare species were insufficient to model detection functions. Population size estimates for several species could only be obtained by pooling together several species of “similar size” and that “provide similar visual cues”. Even so, population estimates could not be calculated at all for some species, such as warthog, which had insufficient sightings to model a detection function and are hard to group with others [11].

The Nakuru Wildlife Forum has conducted biannual (twice a year) game counts throughout the conservancy area since March 1996, with the exceptions of 2001 and 2013 when only one count was conducted and 2015 for which the dry season count was not yet available. Counts on all properties are conducted on the same day and at the same time, as much as possible, to reduce the possibility of double counting animals. The different properties are subdivided into blocks using road networks, vegetation and area to be covered. Teams of counters consisting of at least one driver, one spotter and one recorder drive through each block and record total counts of all medium to large animal species encountered. The counts are undertaken jointly by volunteers from the Kenya Wildlife Service (KWS) and the individual conservancies who know the landscape and animal distribution in the conservancy well. The counts start at 0600 am and end at 1100 am when animals are most active and likely to be encountered in the open landscape. During the counts the conservancy is partitioned into the southern and northern sections, separated by Lake Naivasha and dense human settlements that limit interchange of wildlife between the two sections within the counting period. The counts are done using 4-wheel drive vehicles, on foot where the terrain is not accessible by vehicle due to steep or rugged topography, or vegetation cover and using boats on the shores of Lake Naivasha. The maximum vehicle speed during the surveys is 20 km/h. The counts are done with the aid of binoculars. On average, the entire counting exercise takes about three days to complete. The first day is used to train the new counters on the counting method, and for volunteers to meet the landowners and coordinate the survey logistics. The landowners also use the first day to inform volunteers about any changes in the conservancies or ranches since the last census. Actual counting is then carried out during the next two consecutive days. The expansive northern ranches, including Delamere, Kikopey and Marula ranches and the surrounding areas are covered in one day by about 50 counters and 15 vehicles. The Kedong ranches and other southern properties, including Hell’s Gate National Park, Longonot National Park and Oserian, are also covered in one day by about 50 counters and 20 vehicles. The foot counts are only carried out in the densely vegetated areas that are inaccessible by car, located predominantly in Oserian and Delamere properties and along the Lake Naivasha shoreline in Marula; all together totaling less than 20% of the entire Conservancy area. A boat is used for about one hour only to count buffaloes occurring inside the reeds as well as hippos along the shoreline. The total counts cover virtually all of the NWC as shown in Fig 1b.

Volunteers from a conservancy, park or ranch always count in the same counting blocks but volunteers from outside, including those from KWS, change occasionally as the individual volunteers vary over time. The same tracks are followed as much as possible during the surveys. The counts are done mainly in September-October and March-May of each year. These periods were selected to represent the dry and the wet season conditions, respectively. Availability of green plant biomass for wildlife is highest in March-May and lowest in September-October. The block counts are later summed to obtain total counts for each property and for the whole conservancy to track changes in animal numbers over time. We used all the counts carried out during 1996–2015 for this study. Because the same methods are applied in each count, any potential biases in the counts should be comparable, so that changes in density over time should reliably reflect overall changes. Except for the national parks and the Oserian sanctuary, all the properties constituting the conservancy support both wildlife and livestock, with Marula and Soysambu supporting the largest livestock herds. However, the livestock counts were not made available to us.

### Ethics Statement

All the bimonthly animal censuses were conducted by members of the Nakuru Wildlife Forum, Kenya Wildlife Service staff and volunteers.

### Statistical modeling and data analyses

#### Rainfall

We analysed temporal variation in rainfall from 1964 to 2011 to provide a broad historical context for evaluating rainfall variation during our study period spanning 1996–2015. Monthly rainfall was averaged across all the seven recording stations to account for spatial variation. The resulting average monthly rainfall was used to calculate the total annual (January-December), wet (March-August) and dry (September-January) season rainfall components. It was also used to compute the total quarterly rainfall in terms of the early (March-May) and late (June-August) wet season and the early dry (September-November) and late dry (December-February) season components.

We used an unobserved components model (UCM), a special case of the linear Gaussian state space model [63], to decompose the time series of the annual, wet season and dry season rainfall series (*r*_*t*_) into the trend (*μ*_*t*_), cyclical (*ψ*_*t*_), seasonal (*γ*_*t*_) and irregular (*ϵ*_*t*_) components as follows

$$\begin{array}{ll} r_{t}=\mu_{t}+\psi_{t}+\gamma_{t}+\sum_{j=1}^{m}\beta_{j}x_{jt}+\epsilon_{t}; & t=1,2,\ldots ,n \end{array}$$ (1)

where *β*_*j*_ are regression coefficients, *x*_*jt*_ are regression variables with fixed effects and (*ϵ*_*t*_) are independent and identically (*i*.*i*.*d*) normally distributed errors or disturbances with zero mean and variance $\sigma_{\epsilon }^{2}$. In other words, we assume *ϵ*_*t*_ to be a Gaussian white noise process. The trend, seasonal, cyclical and irregular components are assumed to be statistically independent of each other.

We assume a random walk (RW) model for the time trend, which is equivalent to assuming that the trend (*μ*_*t*_) remains roughly constant through time. The RW trend model is

$$\mu_{t}=\mu_{t-1}+\eta_{t}$$ (2)

where $\eta_{t}\sim \;i.i.d.\;N\left(0,\;\sigma_{\eta }^{2}\right)$. Note that if $\sigma_{\eta }^{2}=0$ then *μ*_*t*_ = *constant*.

We further assume a stochastic cycle (*ψ*_*t*_) with a fixed period (*p* > 2) and damping factor (*ρ*) but a time-varying amplitude and phase specified by

$$\left[\begin{array}{c} \psi_{t}\\ \psi_{t}^{*} \end{array}\right]=\rho \left[\begin{array}{cc} cos\lambda & sin\lambda \\ -sin\lambda & cos\lambda \end{array}\right]\left[\begin{array}{c} \psi_{t-1}\\ \psi_{t-1}^{*} \end{array}\right]+\left[\begin{array}{c} \vartheta_{t}\\ \vartheta_{t}^{*} \end{array}\right]$$ (3)

where 0 ≤ *ρ* ≤ 1, *λ* = 2 × *π*/*p* is the angular frequency of the cycle, *ϑ*_*t*_ and $\vartheta_{t}^{*}$ are independent Gaussian disturbances with zero mean and variance $\sigma_{\vartheta }^{2}$ and 0 < *λ* < *π*. Values of *ρ*, *p* and $\sigma_{\vartheta }^{2}$ are estimated from the data alongside the other model parameters. The damping factor *ρ* governs the stationarity properties of the random sequence *ψ*_*t*_ such that *ψ*_*t*_ has a stationary distribution with mean zero and variance $\sigma_{\vartheta }^{2}/\left(1-\rho^{2}\right)$ if *ρ* < 1 but is nonstationary if *ρ* = 1. We allowed and tested for up to three cycles in the annual, wet season and dry season rainfall components.

In addition to the random walk model (2) we modelled the trend component using a locally linear time trend incorporating both a level and slope and described as

$$\begin{array}{ll} \mu_{t}=\mu_{t-1}+\beta_{t-1}+\eta_{t}, & \eta_{t}∼i.i.d.\;\left(0,\sigma_{\eta }^{2}\right)\\ \beta_{t}=\beta_{t-1}+\xi_{t}, & \xi_{t}∼i.i.d.\;\left(0,\sigma_{\xi }^{2}\right), \end{array}$$ (4)

where the disturbance variances $\sigma_{\eta }^{2}$ and $\sigma_{\xi }^{2}$ are assumed to be independent. The UCM models (1) and (4), without the seasonal and regression components, were fitted by the diffuse Kalman filtering and smoothing algorithm in the SAS UCM procedure [64].

#### Modeling temporal trends in animal density

Because the number of properties participating in the counts, and thus the total area surveyed varied over time, the total number of animals of each species counted in each census was divided by the total area covered by the census to obtain density (number / km^2^) estimates for each species. We therefore analyzed time trend in the density of each species and the biomass density of all herbivores, primates and carnivores. Both the wet and dry season counts were analyzed together because there are no large scale migratory or seasonal dispersal movements, nor substantial seasonal variation in visibility, and hence detectability of the species, due to a preponderance of short to medium grasses.

We modelled trends in the densities of all the wildlife species simultaneously using a flexible semi-parametric model that accommodates irregularly spaced, missing and non-normally distributed counts with many zeroes. The model assumed that the animal counts follow a negative binomial distribution in which the variance is a quadratic function of the mean.

The negative binomial distribution (NB) of animal counts (*Y*) can be given by

$$P\left(Y=y\right)=\frac{\Gamma \left(y+k\right)}{y!\Gamma \left(k\right)}{\left(\frac{\mu }{\mu +k}\right)}^{y}{\left(\frac{k}{\mu +k}\right)}^{k}.$$ (5)

The mean of *Y* is given by *μ* = *E*(*Y*) and its variance by Var(*Y*) = *μ*(1 + *μ*/*k*). *k* is a shape or dispersion parameter and quantifies the amount of overdispersion [65].

Let **z** ≡ (***x***, *u*) be a covariate vector with ***x*** = (*x*_1_, …, *x*_*p*_, *x*_*p*+1_, …, *x*_*q*_), a 1 × (*p* + *q*) covariate vector and *u* a continuous independent variable. Further, denote the mean of *Y* with *u*(***z***) = *E*(*Y*|***z***). Then the negative binomial probability distribution in Eq (5) can be recast in exponential format by

$$P\left(Y=y;z\right)=y\text{log}\left\{\frac{\mu \left(z\right)}{\mu \left(z\right)+k}\right\}-k\text{log}\left(\mu \left(k\right)+k\right)+k\text{log}k+\text{log}\left\{\frac{\Gamma \left(y+k\right)}{\Gamma \left(k\right)}\right\}-\text{log}\left(y!\right).$$ (6)

This formulation shows that the NB model belongs to the exponential family of distributions for known *k*.

Next, we model the temporal trends in the animal counts by letting *u* be an unspecified smooth function of time and the fixed effects in ***x*** to be linear. Assuming a log link function, because the canonical link log[*μ*(***z***)/{*μ*(***z***) + *k*}] implied by Eq (6) can be problematic to work with as it is always negative, the expected counts given the covariates is then specified as

$$\text{log}\left\{\mu \left(z\right)\right\}=\text{x}\beta +\;s_{1}\left(u_{1}\right)+s_{2}\left(u_{2}\right)+1.\text{log}(\text{area})$$ (7)

where ***β*** = (*β*_1_, …, *β*_*p*_, *β*_*p*+1_, …, *β*_*p+q*_)^*T*^ is a 1 × (*p* + *q*)-parameter vector for the *p* + *q* -covariate vector ***x*** = (*x*_1_, …, *x*_*p*_, *x*_*p*+1_, …, *x*_*p+q*_), *s*_1_(·) and *s*_2_(·) are unspecified smooth functions that capture the effects of *u*_1_ and *u*_2_. The logarithm of the total area counted in each census, log(area), is an offset used to calculate animal density. The offset has a coefficient equal to unity by construction. The unspecified smooth functions *s*_1_(·) and *s*_2_(·) are approximated by penalized *B*-spline basis functions as

$$\begin{array}{ll} s_{1}\left(u_{1}\right)=\sum_{l=1}^{L}b_{l}B_{l}\left(u_{1}\right) & ={\text{Z}}_{s_{1}}{\text{u}}_{s_{1}}\\ s_{2}\left(u_{2}\right)=\sum_{j=1}^{J}c_{j}B_{j}\left(u_{2}\right) & ={\text{Z}}_{s_{2}}{\text{u}}_{s_{2}} \end{array}$$ (8)

where *b*_*l*_ and *c*_*j*_ are the penalized cubic *B*-spline coefficients to be estimated. The computational details and mathematical properties of *B*-splines can be found in [66].

We now use the time series of the 44 species monitored in NWC from 1996 to 2015 to clarify how the smooth functions *s*_1_(⋅) and *s*_2_(·) in Eq (8) are approximated by a mixed model. The number of all observations is $n=\sum_{i=1}^{44}n_{i}=1400$, where *n*_*i*_ is the number of all observations for the *i*-th of the total of 44 species counted. Furthermore, *p* = 44 is the number of coefficients to be estimated for the species main effect and *q* = 44 is the number of coefficients representing the species × time interaction effect. Accordingly, the full design matrix of fixed effects ***x*** in Eq (7) has dimension *n* × (*p* + *q*) = [1400 × (44 + 44)] whereas the vector of fixed effect parameters ***β*** has dimension 1 × (*p* + *q*) = 1 × 88.

If we let ${\tilde{\text{Z}}}_{s}$ represent the (*n* × *K*) matrix of *B*-splines of degree *d* and ***D***_*r*_ the (*K* − *r* × *K*) matrix of *r*-th order difference penalty, then the (*n* × *K* − *r*) matrix ${\text{Z}}_{s}\;=\;\left[{\text{Z}}_{s_{1}},{\text{Z}}_{s_{2}}\right]$ used to fit the mixed model specified by 7 and 8 equals

$${\text{Z}}_{s}={\tilde{\text{Z}}}_{s}{\left({\text{D}}_{\text{r}}^{T}{\text{D}}_{r}\right)}^{-}{\text{D}}_{r}^{T}$$ (9)

The total number of *B*-spline knots used to specify ${\tilde{\text{Z}}}_{s}$ equals the number *m* of equally spaced interior knots plus *d* knots placed at the starting date and *max*{1, *d*} knots placed at the ending date of the censuses. The total number of columns in the B-spline basis is thus *K* = *m* +*d* + 1.

The number of variables (columns) representing the penalized *B*-spline (called *P*-spline) random effect of time trend common to all the species is given by $d_{s_{1}}=\left(K-r\right)=\left(m+d+1-r\right)=20+3+1-3=21$. Here, the number of interior knots is *m* = 20, the degree of the B-spline basis is *d* = 3 and the order of differences of the spline coefficients is *r* = 3. The number of variables (columns) representing the random *P*-spline effect for the time trend specific to each species (species interaction × time) is then given by $d_{s_{2}}=d_{s_{1}}\times \;p=21\times 44=924$. It follows that for these data the random effect design matrix ${\text{Z}}_{s_{1}}$ in Eq (8) has dimension $n\;\;\times \;d_{s_{1}}=1400\;\;\times \;21$ whereas ${\text{Z}}_{s_{2}}$ in Eq (8) has dimension $n\;\;\times \;d_{s_{2}}=1400\;\;\times \;924$. The full design matrix of random effects ${\text{Z}}_{s}\;=\;\left[{\text{Z}}_{s_{1}},{\text{Z}}_{s_{2}}\right]$ therefore has dimension $n\;\;\times \;\left(d_{s_{1}}+\;d_{s_{2}}\right)=1400\;\;\times \;\left(21+924\right)$. The vector of parameters of random effects ${\text{u}}_{s_{1}}$ in Eq (8) has dimension $1\;\;\times \;\;d_{s_{1}}=1\;\;\times \;21$ whereas ${\text{u}}_{s_{2}}$ has dimension $1\;\times \;d_{s_{2}}=1\;\times \;924$. The full vector of parameters of random effects $\text{u}={\left({\text{u}}_{s_{1}},{\text{u}}_{s_{2}}\right)}^{T}$ has dimension $1\;\times \left(d_{s_{1}}+d_{s_{2}}\right)=1\;\times 945$. The random effects $u_{1}\sim i.i.d.\;N\left(0,\sigma_{u_{1}}^{2}\right)$ whereas $u_{2}\sim i.i.d.\;N\left(0,\sigma_{u_{2}}^{2}\right)$.

The full model specified by Eqs (7), (8) and (9) can therefore be described as a multivariate, semiparametric generalized linear mixed model. The model is semiparametric because it consists of parametric and non-parametric components. The fixed part of the model is parametric and consists of the species main effect, representing species-specific overall densities, and species-by-time trend interaction, representing species-specific trends. The random part of the model, specifying the non-parametric smooth functions, consists of two continuous random spline effects, each specified by a penalized spline variance-covariance structure. The first random spline effect fits a penalized cubic (*d* = 3) *B*-spline [67] with a third-order difference penalty (*r* = 3) to the random spline coefficients common to all the species and thus models the time trend common to all the species. The second random spline effect similarly fits a penalized cubic B-spline with random coefficients specific to each species and hence models the time trend unique to each species. Each of the two random spline effects had *m* = 20 equally spaced interior knots placed on the running date of censuses (March 1996, …, May 2015) plus 3 evenly spaced exterior knots placed both at the start date and end date of the censuses, for a total of 26 knots. The model yields estimates of three variance components, corresponding to the random spline time trend common to all species $\sigma_{u_{1}}^{2}$, random spline effect for the time trend specific to each species $\sigma_{u_{2}}^{2}$ and the scale parameter for the negative binomial distribution *k*.

Fitting the model exploits the idea that spline smoothing and mixed modeling address equivalent minimization problems and produce the same solutions. However, the solutions for the spline coefficients within the mixed modeling framework are solutions of random effects, not fixed effects, as is the case for solutions of spline coefficients in the classical framework. Standard errors of the predicted counts thus account for the variation in spline coefficients associated with treating the coefficients as random effects in mixed models [68]. A distinct advantage of formulating spline smoothing as a mixed model is that the smoothing parameter is selected ‘automatically’ because it is a function of the covariance parameter estimates produced by the mixed model. The trend model was fitted via maximization of residual penalized quasi-likelihood, called pseudo-likelihood by [69], in the SAS GLIMMIX procedure. The SAS codes (SAS Version 9.4, SAS /STAT version 14.1) used to fit the rainfall and wildlife trend models are provided in Supplementary Materials SM1.

To establish if population density for each species changed significantly between 1996 and 2015 we used constructed spline effects [64] to compare the expected population size for 1996 to that for 2015. For species whose numbers initially increased to a peak and then declined, the expected peak population size was additionally compared with the expected population sizes in March 1996 and May 2015.

#### Relationship between rainfall and animal biomass and density

We calculated the total biomass (kg/km^2^) of all the 32 herbivore species (S1 Table) for each year and related the biomass to 1- to 10-year moving averages of the annual, seasonal and quarterly rainfall components. The 10-year cut-off window was selected to coincide with the period of the longest cycle established for the annual and wet season rainfall components. The cumulative past rainfall component most strongly correlated with the aggregate herbivore biomass or the densities of each of the 30 most abundant species, chosen to minimize potential influences of stochastic noise due to small population sizes of some species, was selected using Pearson correlation coefficients and the corrected Akaike Information Criterion [70].

## Results

### Temporal rainfall patterns

Monthly rainfall distribution is trimodal, with a major peak in April, and two minor peaks in August and November (Fig 2). The total annual (January-December) rainfall averaged 751 ± 156 mm (mean ±1 SD, range 414–1107 mm) during 1961–2011. The wet season (March-August) rainfall averaged 444.3 ±116.6 mm (range 183–721 mm) whereas the dry season (September-February) rainfall averaged 308.7 ± 120.5 mm (range 160–714 mm) in the same period. Based on the annual rainfall, extreme droughts occurred in 1965, 1976, 1984, 1999, 2000 and 2009 whereas severe droughts were experienced in 1971, 1972, 1973, 1980, 1991, 1993 and 2008. Extremely wet years were 1968 and 1998 while 1978, 2010 and 2011 were very wet years. The wet season was extremely dry in 1972, 1984, 1993, 2000, 2008 and 2009 but extremely wet in 1968 and 2011 and very wet in 1967, 1988 and 2003. The dry seasons of 1980, 1981, 1986, 1994 and 2000 were extremely dry but the dry seasons of 1962 and 1998 were extremely wet (Fig 2).

Rainfall variation was quasi-periodic with the oscillation in the annual component having a period of 9.5 years, that in the wet season component having cycle periods of 2.2, 3.5 and 10.9 years, while that in the dry season component having a period of 2.8 years (Tables 1 and 2, S1 Fig). The estimated cycle periods were all highly significantly greater than zero (Table 1). As well, the estimated damping factors were nearly equal to 1, except that for the largest cycle for the wet season rainfall component, which was 0.788. The disturbance (error) variances were also very close to zero for all the three rainfall components (Table 1). These properties jointly imply that the identified rainfall cycles are both persistent and deterministic. However, the estimates for the disturbance variances for the irregular components for the annual and dry season rainfall components are significant, meaning that these irregular components are stochastic (Table 1). All the cyclical components had statistically insignificant disturbance variances (Table 1). However, significance analysis of the disturbance variances of the cyclical components in the model at the end of the estimation span suggests otherwise. If a component is deterministic, then this analysis (Table 2) is equivalent to establishing whether the corresponding regression effect in Table 1 is significant. But if a component is stochastic, then this analysis applies only to the part of the time series of rainfall near the end of the estimation span. Table 2 therefore shows that the cycle in the annual rainfall, the two higher frequency cycles in the wet season rainfall and the single cycle in the dry season rainfall, are significant whereas the low frequency (largest) cycle in the wet season rainfall is not. Since the disturbance (error) variances of all the cyclic components are not significant in Table 1 but are significant in Table 2, except for the largest cycle in the wet season rainfall component, all the identified rainfall cycles are persistent and deterministic. The largest cycle in the wet season rainfall component is somewhat transient because its damping factor of 0.788 is less than 1. The disturbance term corresponding to the level (trend) component is not significant for either the annual, wet or dry season rainfall, suggesting that the trend in none of these rainfall components systematically increased or decreased through time in NWC (Table 2). The smoothed cycles for the cycles with statistically significant periods based on the structural time series analysis for the annual, wet season and dry season rainfall components are plotted in S2 Fig.

**Table 1:** The estimated variances of the disturbance terms, the variances of the irregular component, damping factor and period of the cycles in annual, wet and dry season rainfall components.

Rainfall Component	Model Component	Parameter	Estimate	SE	T	P>|T|
Annual	Irregular	Error Variance $\left(\sigma_{\epsilon }^{2}\right)$	0.8796	0.2019	4.4	1.32 × 10^−5^
	Cycle_1	Damping Factor (*ρ*)	1.0000	0.0004	2846.4	0
	Cycle_1	Period (*p*)	9.4749	0.4885	19.4	8.43 × 10^−84^
	Cycle_1	Error Variance $\left(\sigma_{\vartheta }^{2}\right)$	0.0000	0.0000	0.8	0.45057
Wet Season	Irregular	Error Variance $\left(\sigma_{\epsilon }^{2}\right)$	0.2053	0.2359	0.9	0.384214
	Cycle_1	Damping Factor (*ρ*)	0.7880	0.2656	3.0	0.003007
	Cycle_1	Period (*p*)	10.8666	2.8486	3.8	0.000136
	Cycle_1	Error Variance $\left(\sigma_{\vartheta }^{2}\right)$	0.1614	0.1154	1.4	0.161968
	Cycle_2	Damping Factor (*ρ*)	1.0000	0.0002	4408.4	0
	Cycle_2	Period (*p*)	3.4623	0.0323	107.0	0
	Cycle_2	Error Variance $\left(\sigma_{\vartheta }^{2}\right)$	0.0000	0.0000	0.9	0.357737
	Cycle_3	Damping Factor	1.0000	0.0001	9392.0	0
	Cycle_3	Period (*p*)	2.1633	0.0126	172.3	0
	Cycle_3	Error Variance $\left(\sigma_{\vartheta }^{2}\right)$	0.0000	0.0000	0.9	0.361499
Dry Season	Irregular	Error Variance $\left(\sigma_{\epsilon }^{2}\right)$	0.9814	0.2281	4.3	1.69 × 10^−5^
	Cycle_1	Damping Factor (*ρ*)	1.0000	0.0004	2396.8	0
	Cycle_1	Period (*p*)	2.8077	0.0347	80.9	0
	Cycle_1	Error Variance $\left(\sigma_{\vartheta }^{2}\right)$	0.0000	0.0000	0.8	0.44056

**Table 2:** Significance analysis of components (based on the final state).

Rain	Model Component	DF	*χ*^2^	P > *χ*^2^
Annual	Irregular $\left(\sigma_{\epsilon }^{2}\right)$	1	5.3572	0.0206
Annual	Level $\left(\sigma_{\eta }^{2}\right)$	1	0.0533	0.8174
Annual	Cycle $\left(\sigma_{\vartheta }^{2}\right)$	2	6.2333	0.0443
Wet	Irregular $\left(\sigma_{\epsilon }^{2}\right)$	1	0.1876	0.6649
Wet	Level $\left(\sigma_{\eta }^{2}\right)$	1	0.0397	0.8420
Wet	Cycle_1 $\left(\sigma_{\vartheta }^{2}\right)$	2	2.0159	0.3650
Wet	Cycle_2 $\left(\sigma_{\vartheta }^{2}\right)$	2	23.3386	8.55 × 10^−6^
Wet	Cycle_3 $\left(\sigma_{\vartheta }^{2}\right)$	2	21.6129	2.03 × 10^−5^
Dry	Irregular $\left(\sigma_{\epsilon }^{2}\right)$	1	2.1298	0.1445
Dry	Level $\left(\sigma_{\eta }^{2}\right)$	1	9.75 × 10^−5^	0.9921
Dry	Cycle $\left(\sigma_{\vartheta }^{2}\right)$	2	6.7315	0.0345

The total rainfall recorded at seven rain gauges located within or near the NWC during 1964–2011 is provided in S1 Data. The total monthly rainfall recorded at nine gauges situated within or near Lake Nakuru National Park are likewise provided in S2 Data.

### Wildlife population trends

**Fig 3:**
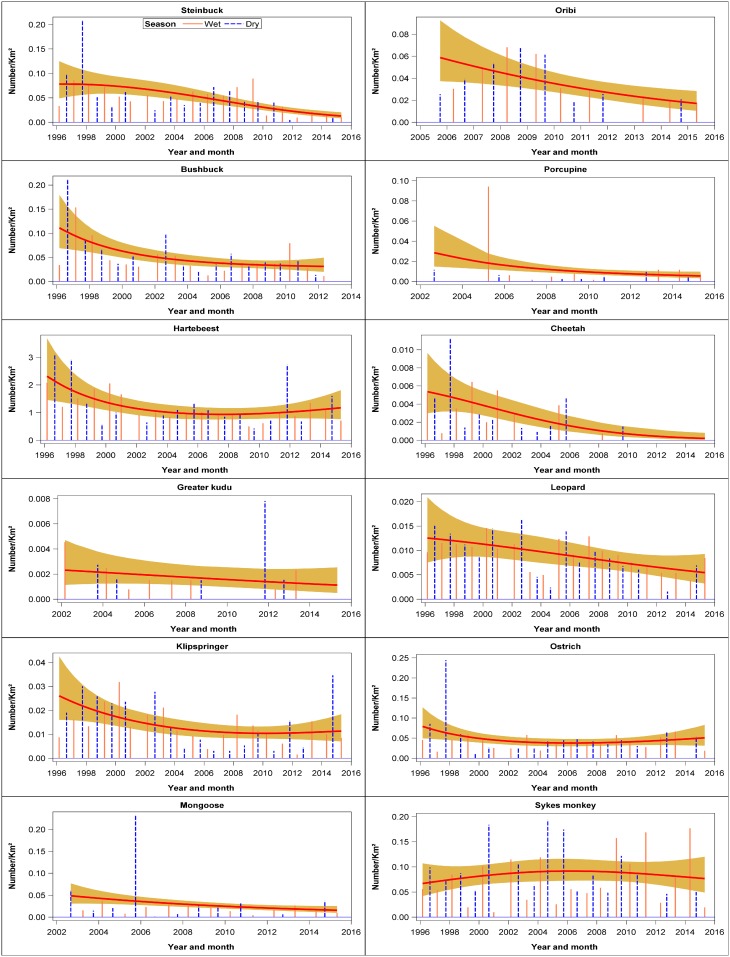
Trends in the density (Number /km^2^) of steinbuck, bushbuck, hartebeest, Greater kudu, klipspringer, mongoose, oribi, porcupine, cheetah, leopard, ostrich and Sykes monkey for the entire Nakuru Wildlife Conservancy during 1996–2015.

**Fig 4:**
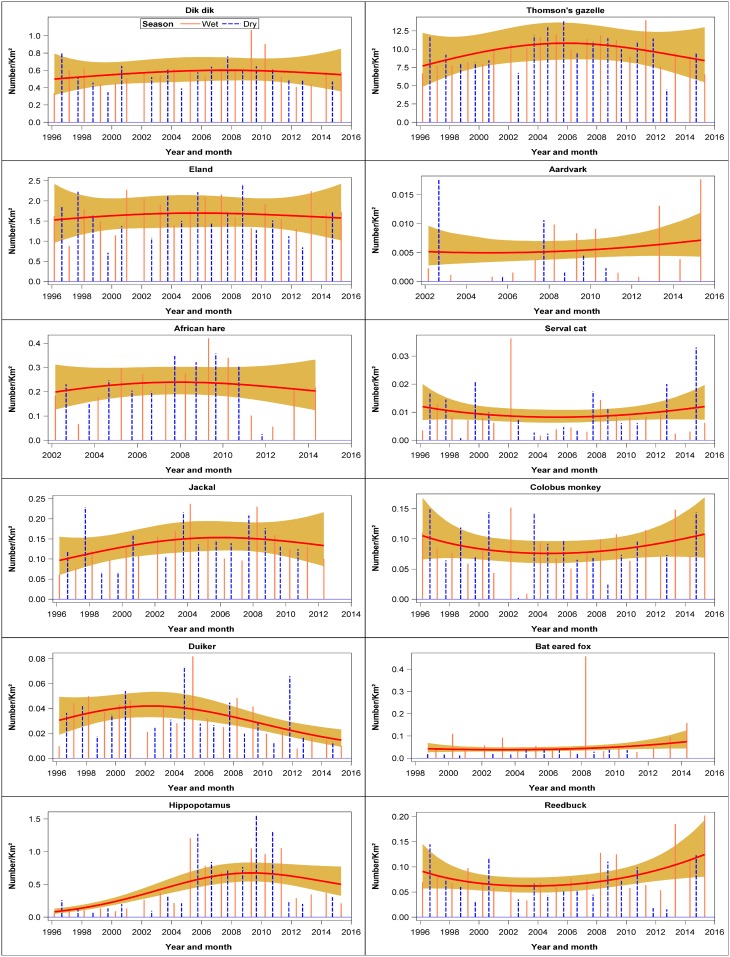
Trends in the density (Number /km^2^) of dik dik, eland, African hare, jackal, duiker, hippopotamus, Thomson’s gazelle, aardvark, serval cat, colobus monkey, bat-eared fox and reedbuck for the entire Nakuru Wildlife Conservancy during 1996–2015.

**Fig 5:**
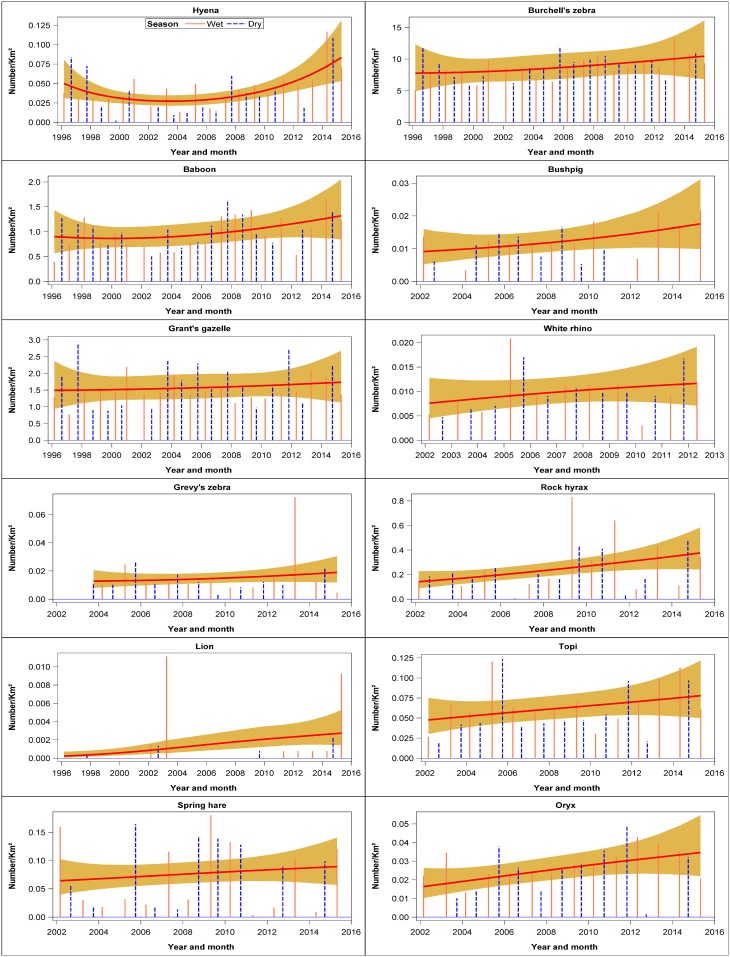
Trends in the density (Number /km^2^) of hyena, baboon, Grant’s gazelle, Grevy’s zebra, lion, spring hare, Burchell’s zebra, bushpig, white rhino, rock hyrax, topi and oryx for the entire Nakuru Wildlife Conservancy during 1996–2015.

**Fig 6:**
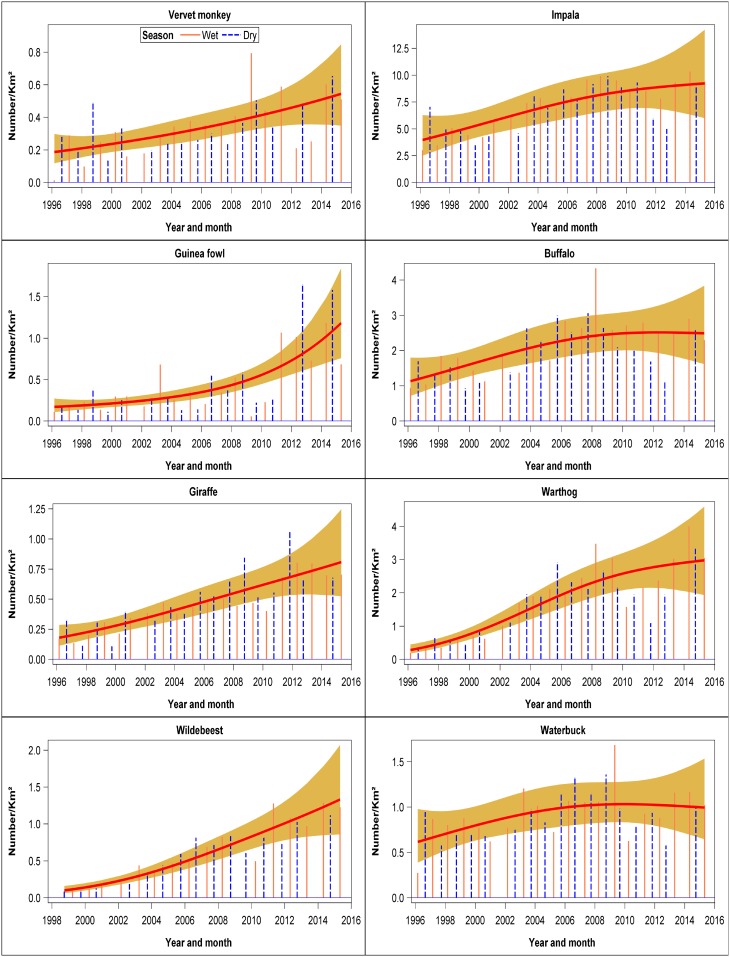
Trends in the density (Number /km^2^) of vervet monkey, guinea fowl, giraffe, wildebeest, impala, buffalo, warthog and waterbuck for the entire Nakuru Wildlife Conservancy during 1996–2015.

There were six evident patterns in the temporal trends shown by the different species. The first pattern was shown by four species that declined persistently throughout 1996–2015. These species consisted of steinbuck, bushbuck, hartebeest and greater kudu (Fig 3, S3 Table). The second pattern characterized 8 species that declined markedly but not significantly during 1996–2015 comprising klipspringer, mongoose, oribi, porcupine, cheetah, leopard, ostrich and Sykes monkey (Fig 3, S3 Table). The third pattern was shown by seven species that initially increased and then declined during 1996–2015. These species comprised dik dik, eland, African hare, jackal, duiker, hippo and Thomson’s gazelle. However, the patterns were only significant for duiker and hippopotamus (Fig 4, S3 Table). The fourth pattern was shown by seven species that first declined and then increased during 1996–2015. The species showing this pattern were aardvark, serval cat, colobus monkey, bat-eared fox, reedbuck, hyena and baboon (Figs 4 and 5, S3 Table). This pattern was significant only for reedbuck and baboon numbers. The fifth pattern was shown by 14 species that increased but not significantly between 1996 and 2015. The species falling in this category are Grant’s gazelle, Grevy’s zebra, lion, spring hare, Burchell’s zebra, bushpig, white rhino, rock hyrax, topi, oryx, vervet monkey, guinea fowl, giraffe, and wildebeest (Figs 5 and 6, S3 Table). This pattern was significant only for rock hyrax, topi, vervet monkey, guinea fowl, giraffe and wildebeest. The sixth and last pattern was shown by impala, buffalo, warthog, topi, and waterbuck whose numbers increased significantly and then seemed to level off between 1996 and 2015 (Fig 6, S3 Table).

The total number of individuals of each species counted throughout the NWC from 1996 to 2015 and the total area covered by the counts are summarized in S3 Data. Corresponding counts for Lake Nakuru National Park covering 1970–2015 and analyzed in detail by [27] for the period 1970–2012 can be found in S4 Data.

**Fig 7:**
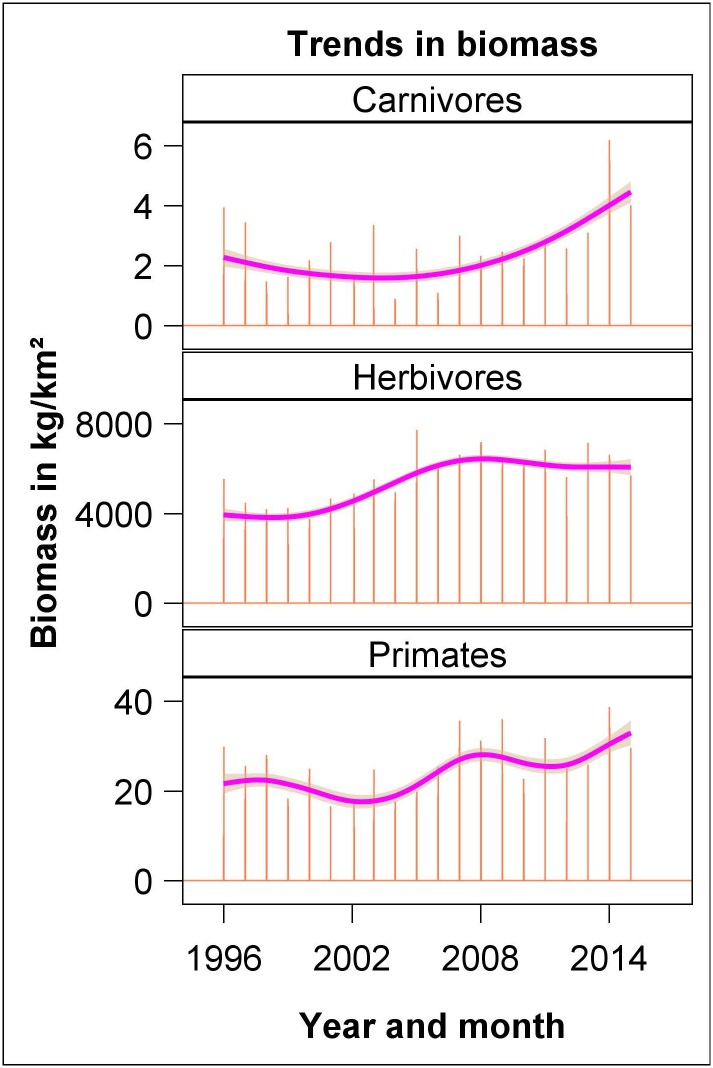
Temporal trends in the aggregate biomass of the 32 herbivore species, 4 primate species and 8 carnivore species counted in the Nakuru Wildlife Conservancy from 1996 to 2015. The species and their unit weights [39] are listed in S2 Table in the supplementary materials. The vertical needles are the biomass estimates, the thick solid line is the trend line and the band around the line is the 95% confidence band fitted by penalized cubic basis spline regression.

The combined carnivore biomass (*n* = 8 species) first decreased and then increased during 1996–2015 whereas the aggregate biomass of all the herbivore species (*n* = 32 species) first increased and then levelled off during the monitoring period. The aggregate biomass of primates (*n* = 4 species) decreased between 1996 and 2000–2001 and then increased before decreasing again from 2008–2009 to 2012 and increasing thereafter (Fig 7).

### Relationship between rainfall and herbivore biomass and density

**Fig 8:**
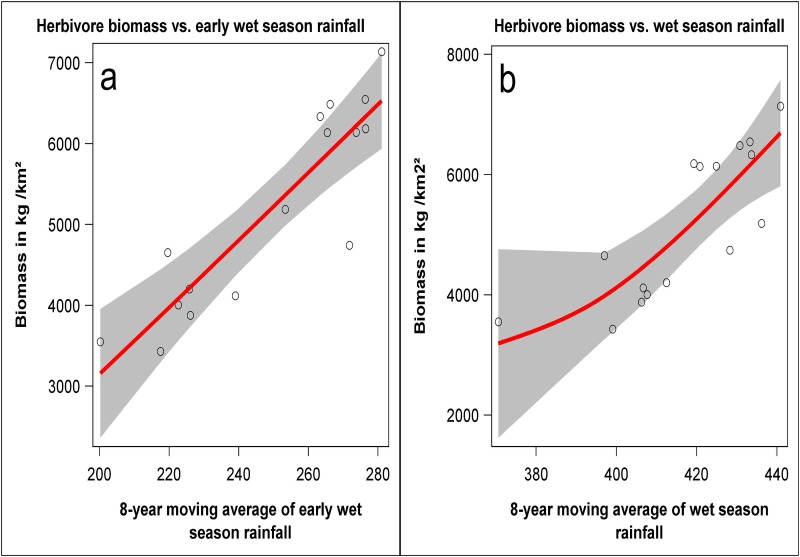
The relationship between the a) aggregate herbivore biomass versus 8-year moving average of the early wet season (March-May) rainfall, *r* = 0.89593, 95% CI: 0.705286–0.961465, *P* = 1.6734 × 10^−7^, *n* = 16 years) and b) aggregate herbivore biomass versus 8-year moving average of the wet season (March-August) rainfall, *r* = 0.79912, 95% CI: 0.482269–0.923621, *P* = 7.7404 × 10^−5^, *n* = 16 years). The 32 herbivore species and the unit weights (in kg) used to calculate aggregate biomass are listed under herbivores in S2 Table in the supplementary materials.

The aggregate herbivore biomass density was most strongly correlated with the 8-year moving average of the early wet season (March-May) rainfall (*r* = 0.8959, 95% confidence limits (CI): 0.7053–0.96145, *P* = 1.6734 × 10^−7^, *n* = 16 years, Fig 8a) and the 8-year moving average of the wet season (March-August) rainfall (*r* = 0.79912, 95% CI: 0.4822–0.9236, *P* = 7.7404 × 10^−5^, *n* = 16 years, Fig 8b). The density of the 30 most abundant species showed three contrasting patterns of correlation with rainfall (S4 Table). The densities of 15 out of the 30 most common species were strongly and positively correlated with rainfall in both the wet and dry seasons. The 15 species showing this pattern were guinea fowl, vervet monkey, Thomson’s and Grant’s gazelles, duiker, impala, warthog, topi, ostrich, waterbuck, wildebeest, Burchell’s zebra, buffalo, hippo and giraffe (S4 Table). In contrast, the densities of seven species, namely African (brown) hare, rock hyrax, Sykes monkey, reedbuck, bushbuck and hyena were strongly negatively correlated with rainfall in both seasons. For the remaining eight species, density was strongly negatively correlated with rainfall in one season but positively correlated with rainfall in the other season. The species displaying this pattern were spring hare, bat-eared fox, dik dik, jackal, colobus monkey, baboon, hartebeest and eland (S4 Table).

## Discussion

### Rainfall

Rainfall in the NWC region is quasi-cyclic with approximate cycle periods varying in length depending on the rainfall component. The cycles in the annual and dry season components can each be adequately described by a single cycle with a period of 9.5 and 2.8 years, respectively. However, the oscillation in the wet season rainfall component is more complex and can best be described by three cycles of periods: 2.2, 3.5 and 10.9 years. The rainfall oscillations are persistent and deterministic and are associated with frequent severe droughts and hence food shortages for herbivores in the region. Although droughts are a recurrent and persistent feature of this region, there was no evidence of systematic increase or decrease in rainfall over time. The aggregate herbivore biomass increased with increasing rainfall, particularly the wet season component, as expected by the herbivore biomass-rainfall relationship characteristic of African savannas [39–41]. The decline in numbers of many wildlife species cannot be due primarily to the rainfall fluctuations because rainfall showed no evident temporal trend nor increasing variability. The declines are therefore most likely caused by the anthropogenic activities undertaken in the NWC.

### Wildlife population trends

The trend analyses showed that numbers of 12 species declined whilst numbers of all the other 32 species were either stable or increased during 1996–2015. The persistently declining species that merit the greatest conservation attention comprise steinbuck, bushbuck, hartebeest and greater kudu. The other eight species that also merit close conservation attention because of their declining numbers in the NWC are klipspringer, mongoose, oribi, porcupine, cheetah, leopard, ostrich and duiker. The persistent decline in cheetah numbers has previously been documented for the NWC [11]. The declining trends are not unique to NWC but are consistent with severe and widespread declines in wildlife numbers recorded for most of the Kenya rangelands [1,2, 28–31, 71–74] and other parts of Africa [75,76]. The increase in numbers of most of the wildlife species in the Nakuru Wildlife Conservancy is noteworthy and similar to the patterns shown by several wildlife species within the adjoining but fully fenced Lake Nakuru National Park [26,27]. Such an increase in numbers of many wildlife species is extremely rare in the human and livestock dominated rangelands of Kenya [77].

Establishing the primary cause (s) of the declines is difficult because environmental and land use changes have not been monitored alongside wildlife numbers. Nevertheless the declines most likely emanate from the manifold anthropogenic activities, including the major land use changes, poaching, fencing, fires, and proximity to the three major towns. Our initial expectation that the numbers of all the wildlife species should be declining in the NWC due to the large-scale and dramatic land transformations (H_1_) was only partially supported because the numbers of many species increased significantly between 1996 and 2015. The land use changes and perimeter fencing degrade and fragment the human-dominated landscape, reducing spatial connectivity necessary for wildlife to move between the south of the Nakuru-Naivasha area and the rest of the conservancy as well as between the different properties within the NWC. Maintenance of spatial connectivity is thus crucial because species such as cheetahs have historically occurred in small numbers in the Nakuru-Naivasha area, and likely relied on NWC as a corridor for moving between the southern part of the country to the central highlands and the Laikipia Plateau [11]. The densely settled areas around Lake Naivasha now make it increasingly difficult for wildlife to engage in such movements. Ongoing intensification of land conversion and subdivision of larger properties surrounding the conservancy present additional and mounting barriers to wildlife movements that increasingly confine wildlife to within isolated habitats. Hence, it is imperative that spatial connectivity is maintained so that wildlife can move freely between the large mosaics of habitats presented by the different connected properties within the NWC. This will enable the conservancy to support wide-ranging wildlife species that the individual properties cannot support in isolation. Thus, even if landholders fence off parts of their properties for exclusive use by livestock or for cultivation, as currently happens, they should always provide pathways for free movement of wildlife within the conservancy landscape.

The other cause of population declines, poaching for bushmeat and skins, is a persistent problem in the Nakuru-Naivasha region [11]. Poaching occurs mainly through large-scale snaring of wildlife for meat and skins and its effects on wildlife populations is compounded with those of major and escalating threats of human-wildlife and land use conflicts [21]. Consequently, there is a pressing need to effectively deter poaching of wildlife. Due to the conflicts, large carnivores have either been extirpated or their population size severely suppressed in the Nakuru-Naivasha area [11], consistent with our findings. The extreme decline in the cheetah population in NWC may thus be due, at least in part, to harassment or killings by livestock farmers, especially since our results also show that lions and spotted hyenas that often outcompete cheetah when occurring in large numbers, were also rare in NWC. This is not surprising because large carnivores kill livestock, injure or kill people [11,78]. Many smaller landowners who are not members of the conservancy are therefore understandably unable and unwilling to absorb the cost of livestock losses, and will harass, poison or even kill carnivores to protect their stock [78]. For their population to recover it is important therefore that wildlife are not harassed or killed if they colonize new properties in the NWC. This will help alleviate a continuing conservation concern in NWC, namely that, whilst members of the NWF are tolerant of wildlife on their properties and even encourage their presence, other landowners in the area are not as favorably disposed.

The population trends also do not support the prediction of the hypothesis (H_2_) that large-bodied herbivores should decline more than their small-bodied counterparts since the large-bodied herbivore numbers either did not decrease (eland) or increased (both zebras, white rhino, hippo, giraffe and buffalo). Our results do not provide evidence of accelerated population increase following the prohibition of population culling in 2002, as anticipated by H_3_, suggesting that culling was not the only primary factor limiting population growth in the NWC.

The aggregate biomass analysis showed that primates, herbivores and carnivores, considered as groups, had increased considerably between 1996 and 2015, implying that the contributions of the declining species to the aggregate biomass was more than compensated for by the increasing species. The strong and significant correlation between the aggregate herbivore biomass and the wet season rainfall implies that a reduction in rainfall or increased frequency and severity of droughts, as may occur as a consequence of climate change linked to global warming, would lead to a reduction in the population abundance of herbivores in the region and hence also the abundance of the large carnivores that depend on them.

The aggregate herbivore biomass increased significantly with increasing cumulative past rainfall, as predicted by H_4_. However, the densities of the 30 most abundant species showed either positive or negative correlations with cumulative past rainfall, implying that increasing rainfall and the associated changes in food availability and quality and habitat suitability would have positive effects on some species but negative effects on others. High rainfall can negatively affect wildlife abundance through flooding of the shoreline grasslands used by herbivores in the dry season, denning and other resting sites of wildlife.

In addition to the anthropogenic activities and rainfall, the other potential causes of the wildlife population declines are trophic interactions, including competition and predation. It is unlikely that predation by large carnivores was responsible for the declines because the large carnivores themselves were few and their numbers were also declining. Interspecific competition between the wild herbivores and livestock could have contributed to the declines if livestock are kept at high densities. However, without data on livestock numbers we could not quantitatively explore this potential cause of wildlife population decline. Even so, it is important that livestock stocking rate be regulated in the conservancy to minimize the risk of habitat degradation through overstocking and over grazing. The competitive relations between species and susceptibility of species to predation can be modified by changes in habitat suitability associated with rainfall variability.

#### The role of conservancies in community-based wildlife conservation and management

The conservation efforts in the NWC spearheaded by various agencies under the auspices of the Nakuru Wildlife Forum had evidently positive effects on the 32 wildlife species whose numbers were either stable or increasing but not on the 12 species whose numbers were declining. It is unlikely that the population increases were due solely to the two small national parks within the NWC, which made relatively minor contributions to the total wildlife population in the NWC throughout 1996–2015. In fact, the number of individuals of each species counted outside the protected National Parks as a percent of the total number counted both inside and outside the parks from 1996 to 2015 averaged 92.8% ±13.2%, emphasizing the importance of the private and communal lands to the success of conservation efforts. This success reinforces the view that private and communal lands play a pivotal role in wildlife conservation in the NWC. The private and communal lands are not only important to conservation but are becoming increasingly more so, not only in NWC but throughout most of Kenya’s pastoral lands that cover about 88% of the terrestrial land surface of Kenya and support between 65 and 70% of all wildlife in Kenya [1,2]. By comparison, officially protected wildlife reserves and parks cover a mere 10–12% of Kenya [77,79].

Conservation in the NWC is due to the collective action of the landholders working in partnerships with governmental, international and non-governmental organizations under the umbrella of the Nakuru Wildlife Forum [21,24]. Similar collaborative arrangements are also promoting wildlife conservation on private and communal lands in Laikipia, Masai Mara, Amboseli and other parts of Kenya. Given the increase in numbers of many wildlife species in the NWC, it is tempting to conclude that collaborative conservation had a generally positive impact on wildlife population performance in the NWC. However, the decline in some wildlife species and other growing rafts of challenges call for carefully re-examining and improving the conservation and management strategies used by members of the NWF to minimize further wildlife losses.

A fundamental question is the extent to which such collaborative management protects and promotes healthy wildlife populations and ecosystems. The involvement of local landowner associations bringing together private, communal and public landowners in the conservation and management of biodiversity outside protected areas is critical because most biodiversity lies outside the protected areas in East Africa. The community associations are gaining prominence as government resources available for conservation become increasingly inadequate in the wake of a growing raft of challenges. The land owner associations are well placed to drive collaborative and locally-based conservation initiatives given their detailed knowledge, skills and strong vested interests in the success of the conservancies. The associations enable individual landowners to integrate their land parcels into broader landscape and regional biodiversity conservation frameworks. By choosing to conserve and protect wildlife, landowners greatly expand the area available for conservation and critical ecosystem services in rural landscapes, buffer protected areas from surrounding human impacts and complement the limited capacity and skills of government agencies [79].

Conservancies and landowner associations that conserve and manage them are emerging as the centerpiece of conservation practice, policies and strategies in pastoral lands of Kenya and should be encouraged and supported as much as possible. The varied circumstances of the different conservancies and the landowners allow for pluralistic and locally-adaptive solutions to conservation challenges prevailing in specific localities. The future success of the conservancies will most strongly hinge on whether and how well wildlife conservation evolves to become a major component of sustainable livelihoods in the rangelands. This is likely to happen if landowners are able to derive meaningful income from wildlife conservation. Currently, land owners in the NWC, like elsewhere in Kenya, are faced with limited variety in sources of income from wildlife after KWS withdrew, in 2002, the wildlife use rights granted to the Nakuru-Naivasha region in 1992. Yet, landowners or landholders incur considerable costs by allowing wildlife to compete with their livestock without expecting any compensation from the government that owns wildlife, thereby enabling populations of many wildlife species to flourish outside the small protected areas. Moreover, most properties in NWC lack natural water sources in the dry season and pump water from rivers, boreholes and the lake for both wildlife and livestock. This is another substantial cost borne entirely by the landholders. Additionally, the landowners provide security for wildlife on their properties. Currently, a number of properties in NWC derive some benefits from wildlife by operating various ecotourism facilities.

Kenya’s Parliament passed a new Wildlife Conservation and Management Act in 2013 that provides new legislative, administrative and policy frameworks and principles for devolving rights and responsibilities for wildlife conservation, management, utilization, habitat recovery and restoration efforts and compensation for losses caused by wildlife in Kenya [80]. The Act advocates principles, policies and practices that promote co-existence between people and wildlife and expand the range of benefits of wildlife conservation and offset wildlife-related losses to those living with wildlife. By aiming to turn wildlife from a liability to an asset for individuals or communities living with wildlife and giving legal backing to organizations such as NWF, the new Act is stimulating more effective engagement in, and support for, collaborative wildlife conservation and management in human-dominated and modified landscapes of Kenya by private and communal land owners.

## Conclusions

It is important that regular monitoring, as carried out by NWF, is continued. The monitoring data should be analyzed to regularly audit the status and performance of the wildlife populations and establish if the policies and goals of the new wildlife Act are being met. This will allow early detection of changes of conservation concern and timely interventions. It is noteworthy that even though some species (n = 12) are declining, many (n = 32) are also doing well in the NWC landscape. This success highlights the importance of 1) community organizations, such as the NWF, in coordinating conservation activities in and between conservancies, 2) landowners being able to benefit directly economically from conserving wildlife on their lands (e.g., through income and employment) so that they are able to meet the costs of conserving wildlife and 3) devolution of wildlife conservation and management responsibilities and opportunities to landholders, with the national Kenya Wildlife Service playing a regulatory and supporting role to ensure adoption of best practices in management, governance, sustainability and community engagement in private and community conservancies. The promise of success of this general conservation strategy is replicated in the pastoral lands of Masai Mara and Laikipia regions of Kenya. This success is catalyzing widespread acceptance, rapid expansion of land under wildlife conservancies in Kenya and a general feeling of renewed hope and interest in wildlife conservation in private and communal pastoral lands. Thus, according to the Kenya Wildlife Conservancies Association (KWCA), 177 communal and private wildlife conservancies covering 62,281 km^2^ or 10.71% of Kenya’s land surface had been formed by 2016 and benefit about 700,000 people nationally. But several hurdles and challenges remain. Several innovate refinements of this general strategy that enable landholders to benefit from and offset the cost of conserving wildlife in areas with relatively limited tourism potential are thus being tried and tested. For example, the Northern Rangeland Trust (NRT), is supporting conservancies to experiment with various options to enhance economic sustainability of conservancies, such as by enhancing benefits that landholders can get from livestock in conservancies with both livestock and wildlife and by investing in various conservation friendly enterprises. It is imperative that governments and wildlife conservation funding organizations seriously consider instituting sustainable financing mechanisms to supplement the efforts of private investors in tourism enterprises who pay fees to landowners to lease land for conservancies, especially when there is a slump in tourism. The success of conservancies is too crucial to the future of conservation outside parks and reserves in Africa to be borne only by a few private investors in tourism because national parks and game reserves are typically too small for many large wildlife, which must access key habitats outside the protected areas either seasonally or year-round. The conservancies are thus playing key roles in providing access to more functional landscapes for wildlife and pastoral livestock and in supporting rural livelihoods.

## Supporting Information

S1 Data Total monthly rainfall in mm at seven recording stations within or neighbouring Nakuru Wildlife Conservancy covering 1961–2011. The seven recording stations within the Lake Naivasha catchment basin comprised Nakuru Meteorological Station (1964–2011), Naivasha Water Bailiff (1961–2011), National Animal Husbandry Research Centre-Naivasha (1970–2011), Naivasha W.D.D. (1970–2008), Gilgil Railway Station (1970–2001), Marula Estate—Naivasha (1970–2000), Soysambu Estate—Elementaita (1970–2010).
(XLSX)

S2 Data Total monthly rainfall recorded at nine stations located within or near Lake Nakuru National Park from 1967 to 2015. For further details see [27].
(XLSX)

S3 Data Total number of individuals of each wildlife species counted throughout the Nakuru Wildlife Conservancy from 1996 to 2015. The month and year in which the census was conducted and the total area covered by each survey in hectares and square kilometers.
(XLS)

S4 Data The total number of the common wildlife species counted in Lake Nakuru National Park from 1970 to 2015. Zebra refers to plains zebra and giraffe to Rothschild giraffe. For further details see Ogutu et al. (2012).
(XLSX)

S1 Fig Pictures of 17 of the 18 common wildlife species in the study region (Flamingoes, Secretary bird, Rock hyrax, Thomson’s gazelle, Grant’s gazelle, impala, warthog, topi, ostrich, wildebeest, Coke’s hartebeest, oryx, waterbuck, Burchell’s zebra, eland, white rhino, Grevy’s zebra, black rhino, buffalo, hippos, Masai giraffe, banded moongose, bat-eared fox, serval cat, silver backed jackal, cheetah, leopard, stripped hyena, spotted hyena, lion, monkey and olive baboon. Photo Credit: Reto Buehler took all the photos except the photo of Thomson’s gazelle that was taken by Niels Mogensen.
(PDF)

S2 Fig The smoothed cycles for the cycles with statistically significant periods based on the structural time series analysis for the annual, wet season and dry season rainfall component. The shaded bands around the smoothed solid curves are the 95% confidence bands.
(PDF)

S1 Table The common names, scientific names, grouping, unit weights, mean density (number / km^2^) and its standard deviation for the 44 common wildlife species counted in the Nakuru Wildlife Conservancy (NWC) from 1996 to 2015. Under dietary guild, O = Omnivore, M = mixed grazer/browser, G = pure grazer and B = pure browser.
(DOCX)

S2 Table The names of the national parks, private conservancies or game ranches in which wildlife were counted in the Nakuru Wildlife Conservancy from 1996 to 2015. (DOCX)

S3 Table The expected density of each of the 44 wildlife species in 1996 and 2015 and the difference between the two estimates and test of significance of their difference based on constructed penalized cubic B-splines. For species that first increased and then declined, the expected density at the time of peak density and its differences from the estimates for 1996 and 2015 were similarly computed and tested for significance.
(XLSX)

S4 Table The Pearson correlations between the density of each of the 30 most abundant wildlife species and cumulative moving averages of past rainfall for the annual, seasonal (dry and wet season) and quarterly (early wet and late wet) components. Each rainfall component was lagged by 0 to 10 years. Only the strongest correlations for each species are provided. For each species separate correlations were computed for the wet and the dry season counts.
(XLSX)

S1 File SAS (Version 9.4) program code used to modeling trend and cycles in the wet season rainfall data using the Unobserved Components Model (UCM) UCM procedure (version 14.1) and to simultaneously model the population trends for the 44 wildlife species using the GLIMMIX procedure (version 14.1). (DOCX)

## References

[1] Ottichilo WK, Grunblatt J, Said MY, Wargute PW (2000) Wildlife and livestock population trends in the Kenya rangeland In: Prins HHT, Grootenhuis JG, Dolan TT, editors. Wildlife conservation by sustainable use.Netherlands: Springer pp. 203–218.

[2] Western D, Russell S, Cuthill I (2009) The status of wildlife in protected areas compared to non-protected areas of Kenya. PLoS One, 4(7), e6140 10.1371/journal.pone.0006140 19584912 PMC2702096

[3] Meinertzhagen R Kenya Diary (1902–1906) (1957, p. 82). Edinburgh: Oliver and Boyd.

[4] Percival AB (1928) A game Ranger on Safari. London: Nesbit & Co Ltd.

[5] Chapman A. (1908) On Safari, Edward Arnold, London.

[6] Simon N (1962) Between the sunlight and the Thunder: The Wild Life of Kenya. London: Collins.

[7] Eliot C (1905) The East African protectorate. Edward Arnold, London.

[8] Percival AB (1924) A game Ranger’s Note Book. London: Nesbit & Co Ltd.

[9] Gosling LM (1969) The last Nakuru hartebeest. Oryx 10: 173–174.

[10] Janis MW, Clark JD (2002) Responses of Florida panthers to recreational deerand hug hunting. Journal of Wildlife Management 66: 839–848.

[11] Evans MM (2004) Land use and prey density changes in the Nakuru WildlifeConservancy, Kenya: Implications for cheetah conservation. M.Sc. Thesis, University of Florida, USA. http://www.carnivoreconservation.org/files/thesis/evans_2004_msc.pdf

[12] Brown JR, Archer S (1989) Woody plant invasion of grasslands: establishment of honey mesquite (*Prosopis glandulosa* var. *glandulosa*) onsites differing in herbaceous biomass and grazing history. Oecologia 80: 19–26 10.1007/BF00789926 23494340

[13] Hudak AT (1999) Rangeland mismanagement in South Africa: failure to applyecological knowledge. Human Ecology 27: 55–78.

[14] Milton SJ, Dean WRJ (1995) South Africa’s arid and semiarid rangelands:Why are they changing and can they be restored? Environmental Monitoring andAssessment 37: 245–264. 10.1007/BF00546893 24197853

[15] Kellner K, Bosch OJH (1992) Influence of patch formation in determining the stocking rate for southern African grasslands. Journal of Arid Environments 22: 99–105.

[16] Everard M, Harper D (2002) Towards the sustainability of the LakeNaivasha Ramsar site and its catchment. Hydrobiologia 488: 191–203.

[17] Myers N (1974) The ecologic/socio-economic interface of wildlife conservationin emergent Africa: Lakes Nakuru and Naivasha in Kenya. Journal ofEnvironmental Economics and Management 1: 319–334.

[18] Harper DM, Harper MM, Virani MA, Smart A, Childress PB (2002).Population fluctuations and their causes in the African Fish Eagle, (*Haliaeetus vocifer* (Daudin)) at Lake Naivasha, Kenya. Hydrobiologia 488: 171–180.

[19] Owino AO, Bennun LA, Nasirwa O, Oyugi JO (2002) Trends in waterbirdnumbers in the Southern Rift Valley of Kenya, 1991–2000. Waterbirds 25 191–202.

[20] IUCN-EARP (2003) Management of invasive species in waterbird habitat inLake Naivasha, Kenya: Report on the restoration of wetlands that aremigratory bird habitats, and that have been damaged by invasive weeds. Unpublished Report.

[21] Odada E, Becht R, Higgins S (2003) Experiences and lessons learned brieffor lake Naivasha. http://www.worldlakes.org/uploads/Naivasha_draft_10.28.03.pdf

[22] Abiya I (1996) Towards sustainable utilization of Lake Naivasha, Kenya. Lakesand Reservoirs: Research and Management 2: 231–242.

[23] Mailu S, Bernard K, Ruto E, Nyangena W (2010) Effect of croppingpolicy on landowner reactions towards wildlife: a case of Naivasha area, Kenya. http://mpra.ub.uni-muenchen.de/21308/1/MPRA_paper_21308.pdf.

[24] SPARVS Agency Ltd (2008) A final report on threat reduction assessment. November 2008. http://http.itc.nl/pub/naivasha/PolicyNGO/SPARVS2008.pdf

[25] Kutilek MJ (1974) The density and biomass of large mammals in Lake NakuruNational Park. African Journal of Ecology 12: 201–212.

[26] Mwangi EM (1998) Large herbivore dynamics in the face of insularization: thecase of Lake Nakuru National Park, Kenya. African Journal of Ecology,36: 276–279.

[27] Ogutu JO, Owen-Smith N, Piepho H-P, Kuloba B, Endebe J (2012) Dynamics ofungulates in relation to climatic and land use changes in an insularizedAfrican savanna ecosystem. Biodiversity and Conservation 21:1033–1053.

[28] Ogutu JO, Piepho H-P, Dublin HT, Bhola N, Reid RS (2009) Dynamics of Mara-Serengeti ungulates in relation to land use changes. Journal of Zoology 278: 1–14.

[29] Ogutu JO, Owen-Smith N, Piepho HP, Said MY (2011) Continuing wildlife population declines and range contraction in the Mara region of Kenya during 1977–2009. Journal of Zoology 285: 99–109.

[30] Ogutu JO, Owen-Smith N, Piepho H-P, Said MY, Kifugo, Reid RS (2013)Changing wildlife populations in Nairobi National Park and adjoiningAthi-Kaputiei Plains: collapse of the migratory wildebeest. The OpenConservation Biology Journal. 7: 11–26.

[31] Ogutu JO, Piepho H-P, Said MY, Kifugo SC (2014) Herbivore Dynamics and Range Contraction in Kajiado County Kenya: Climate and Land Use Changes, Population Pressures, Governance, Policy and Human-wildlife Conflicts. Open Ecology Journal 7: 9–31.

[32] Rutherford MC (1980) Annual plant production—precipitation relations in aridand semi-arid regions. South African Journal of Science 76: 53–56.

[33] Deshmukh IK (1984) A common relationship between precipitation and grasslandpeak biomass for east and southern Africa. African Journal of Ecology 22:181–6.

[34] Boutton TW, Tieszen LL, Imbamba SK (1988a) Seasonal changes inthe nutrient of East African grassland vegetation. African Journal of Ecology 26: 103–115.

[35] Boutton TW, Tieszen LL, Imbamba SK (1988b) Biomass dynamics ofgrassland vegetation in Kenya. African Journal of Ecology 26: 89–101.

[36] Georgiadis N, McNaughton SJ (1990) Elemental and fibre contents ofsavanna grasses: variation with grazing, soil type, season and species. Journalof Applied Ecology 27: 623–634.

[37] Davenport ML, Nicholson SE (1993) On the relation between rainfall and the Normalized Difference Vegetation Index for diverse vegetation types in East Africa. International Journal of Remote Sensing 14: 2369–2389.

[38] Ogutu JO, Piepho H-P, Dublin HT, Bhola N, Reid RS (2008) El Niño—Southern Oscillation, rainfall, temperature and normalised difference vegetation index fluctuations in the Mara—Serengeti ecosystem. African Journal of Ecology 46: 132–143.

[39] Coe MJ, Cumming DH, Phillipson J (1976) Biomass and production of largeAfrican herbivores in relation to rainfall and primary production. Oecologia 22:341–354 28308896 10.1007/BF00345312

[40] East R. (1984). Rainfall, soil nutrient status and biomass of large African savanna mammals. African Journal of Ecology 22: 245–70.

[41] Fritz H, Duncan P (1994) On the carrying capacity for large ungulates of African savanna ecosystems. Proceedings of the Royal Society B 256: 77–82. 10.1098/rspb.1994.0052 8008761

[42] Ogutu J O, Owen-Smith N (2006) Oscillations in large mammal populations: arethey related to predation or rainfall? African Journal of Ecology 43: 332–339.

[43] Owen-Smith N (1990) Demography of a large herbivore, the greater kudu, inrelation to rainfall. Journal of Animal Ecology 59: 893–913.

[44] Ogutu JO, Owen-Smith N (2003) ENSO, rainfall and temperature Influences onextreme population declines among African savanna ungulates. EcologyLetters 6: 412–419.

[45] Bennun L, Nasirwa O (2000) Trends in waterbird numbers in the southernRift Valley, Kenya. Ostrich 71: 220–226.

[46] Verschuren D, Laird KR, Cumming BF (2000) Rainfall and drought inequatorial east Africa during the past 1,100 years. Nature 403: 410–414. 10.1038/35000179 10667789

[47] Everard M, Vale JA, Harper DM, Tarra-Wahlberg H (2002) Thephysical attributes of the Lake Naivasha catchment rivers. Hydrobiologia, 488,13–25.

[48] Owino AO, Oyugi JO, Nasirwa OO, Bennun LA (2001) Patterns of variation inwaterbird numbers on four Rift Valley lakes in Kenya, 1991–1999. Hydrobiologia 458: 45–53.

[49] Sikes HL (1989) Notes of the hydrology of Lake Naivasha. Journal of EastAfrica and Uganda Natural History Society 13: 74–89.

[50] Agustina SR (2008) Land use/Farming system and livelihood changes in theupper catchment of Lake Naivasha, Kenya. M.Sc. Thesis, ITC, TheNetherlands.

[51] Were KO (2008) Monitoring spatio-temporal dynamics of land cover changesin Lake Naivasha drainage basin, Kenya. M.Sc. Thesis, ITC, The Netherlands.

[52] Kutilek MJ (1979) Forage-habitat relations of nonmigratory African ungulates inresponse to seasonal rainfall. Journal of Wildlife Management 43: 899–908.

[53] Foster JB, Kearney D. Nairobi National Park game census, 1966 (1967). EastAfrican Wildlife Journal 5: 112–120.

[54] Foster JB, McLaughlin R. Nairobi National Park game census, 1967 (1968). EastAfrican Wildlife Journal 6: 152–154

[55] Foster JB, Coe MJ. (1968) The biomass of game animals in Nairobi NationalPark, 1960–66. Journal of Zoology 155: 413–25.

[56] McLaughlin RT (1970) Nairobi National Park game census, 1968. East AfricanWildlife Journal 8: 203.

[57] Reid RS, Gichohi H, Said MY, Nkedianye D, Ogutu JO, Kshatriya M, et al (2008) Fragmentation of a peri-urban savanna, Athi-Kaputiei Plains, Kenya In: Galvin KA, Reid RS, Behnke RH, Hobbs NT Ed. Fragmentation in arid and semi-arid landscapes:Consequences for humans and natural systems. Dordrecht, Springer, pp: 195–224.

[58] Mills MGL, Biggs HC, Whyte IJ (1995) The relationship between rainfall, lionpredation and population trends in African herbivores. Wildlife Research 22:75–88.

[59] Owen-Smith N, Mills MGL (2006) Manifold interactive influences on thepopulation dynamics of a multispecies ungulate assemblage. EcologicalMonographs: 76: 73–92.

[60] Dublin HT, Ogutu JO (2015) Population regulation of African buffalo inthe Mara-Serengeti Ecosystem. Wildlife Research 42:382–393.

[61] Metzger KL, Sinclair ARE, Hilborn R, Hopcraft JGC, Mduma SAR (2010) Evaluating the protection of wildlife in parks: the case of African buffalo in Serengeti. Biodiversity and Conservation 19: 3431–3444.

[62] Kruger JM, Reilly BK, Whyte IJ (2008) Application of distance sampling toestimate population densities of large herbivores in Kruger National Park. Wildlife Research 35: 371–376.

[63] Durbin J, Koopman SJ (2012) Time Series Analysis by State SpaceMethods. Oxford, UK: Oxford University Press.

[64] SAS Institute (2016). SAS system for windows (Version 9.4, SAS/STAT version 14.1). SAS Institute Inc Carey, NC, USA.

[65] Hilbe JM (2007) Negative Binomial Regression. New York: CambridgeUniversity Press.

[66] De Boor C (2001) A Practical Guide to Splines, Rev. Edition, New York:Springer-Verlag.

[67] Eilers PHC, Marx BD (1996) Flexible Smoothing with B-Splines andPenalties, with discussion. Statistical Science 11: 89–121.

[68] Ruppert D, Wand MP, Carroll RJ (2003) Semiparametric regression. Cambridge: Cambridge University Press.

[69] Wolfinger R, O'Connell M (1993) Generalized linear mixed models apseudo-likelihood approach. Journal of statistical Computation andSimulation 48: 233–243.

[70] Burnham KP, Anderson DR (2002) Model selection and multimodelinference: a practical information-theoretic approach. Springer.

[71] Bhola N, Ogutu JO, Reid RS, Olff H, Piepho H-P, Hobbs NT (2012)Comparative changes in density and demography of large herbivores inMaasai Mara Reserve and the adjacent Koyiaki Pastoral Ranch, Kenya. Biodiversity Conservation 21: 1509–1530.

[72] Homewood K, Lambin EF, Coast E, Kariuki A, Kikula I, Kivelia J, et al (2001) Long-term changes in Mara-Serengeti wildlife and land cover: pastoralists, population or policies? Proceedings of the National Academy of Science 98: 12544–9. 10.1073/pnas.221053998 PMC60090 11675492

[73] Norton-Griffiths M (2000) Wildlife losses in Kenya: an analysis of conservation policy. Natural Resources Modeling 13:1–16.

[74] Ottichilo WK, de Leeuw J, Prins HHT (2001) Population trends of resident wildebeest (Connochaetes taurinus hecki) and factors influencing them in the Masai Mara ecosystem, Kenya. Biological Conservation 97: 271–82.

[75] Craigie ID, Baillie JEM, Balmford A, Carbone C, Collen E, Green RE, et al (2010). Large mammal population declines in Africa protected areas. Biological Conservation 143: 2221–8.

[76] Scholte P (2011). Towards understanding large mammal population declines inAfrica’s protected areas: A west-central African perspective. Tropical Conservation Science 1: 1–11.

[77] Ogutu JO, Piepho H-P, Said MY, Ojwang GO, Njino LW, Wargute PW(2016) Extreme wildlife declines and concurrent increase inlivestock numbers in Kenya: What are the causes?. PloS one. 11 (9): e0163249. 27676077 10.1371/journal.pone.0163249 PMC5039022

[78] Marker LL, Mills MGL, MacDonald DW (2003) Factors influencingperceptions of conflict and tolerance toward cheetahs on Namibian farmlands. Conservation Biology 17: 1290–1298.

[79] Western D, Waithaka J, Kamanga J (2015) Finding space for wildlife beyond national parks and reducing conflict through community-based conservation: the Kenya experience. Parks 21.

[80] Republic of Kenya (2013) The Wildlife Conservation and Management Act 2013. Kenya Gazette Supplement No. 181, Acts No. 47, Sixth Schedule. Nairobi. http://kenyalaw.org/kl/fileadmin/pdfdownloads/Acts/WildlifeConservationandManagement%20Act2013.pdf. Kenya Gazette Supplement No. 18/ (Acts No. 47). Republic of Kenya.

