# Supplementary figures and images for: Wildlife Population Dynamics in Human-Dominated Landscapes under Community-Based Conservation: The Example of Nakuru Wildlife Conservancy, Kenya
Source: PLoS One. 2017 Jan 19;12(1):e0169730. doi: 10.1371/journal.pone.0169730 (PMC5245813; doi:10.1371/journal.pone.0169730)

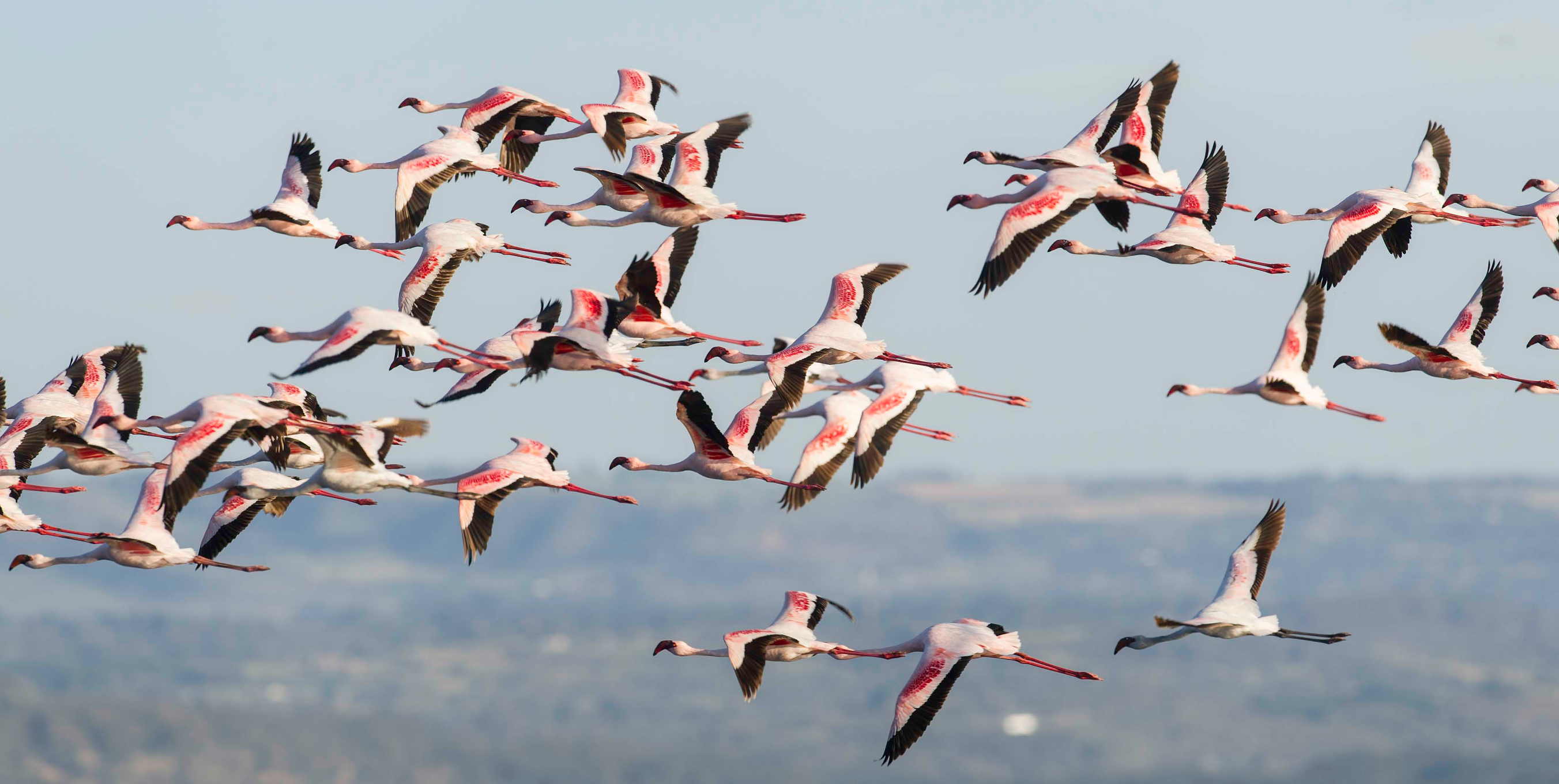

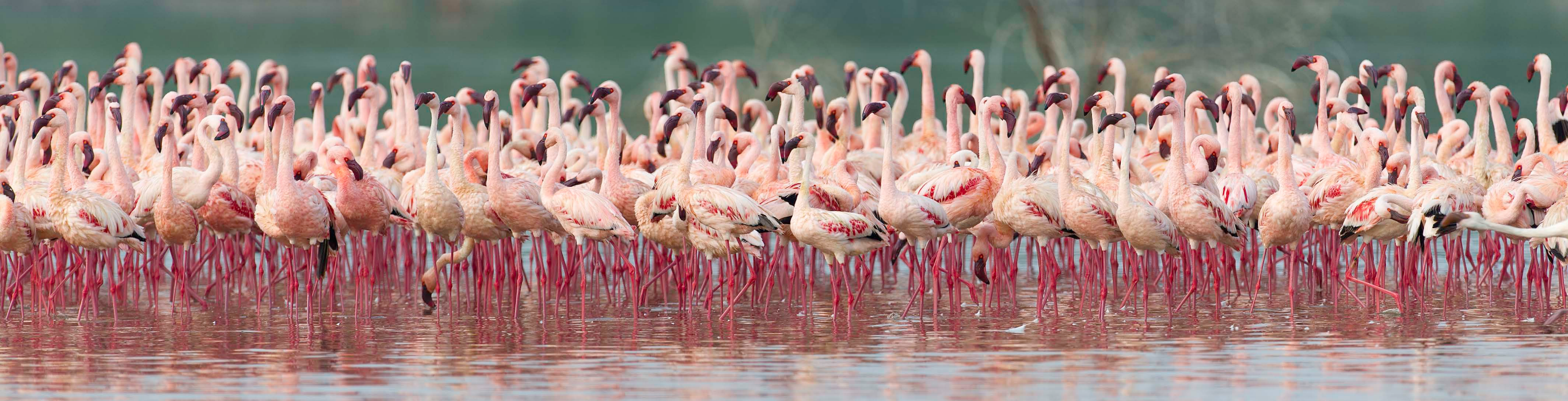

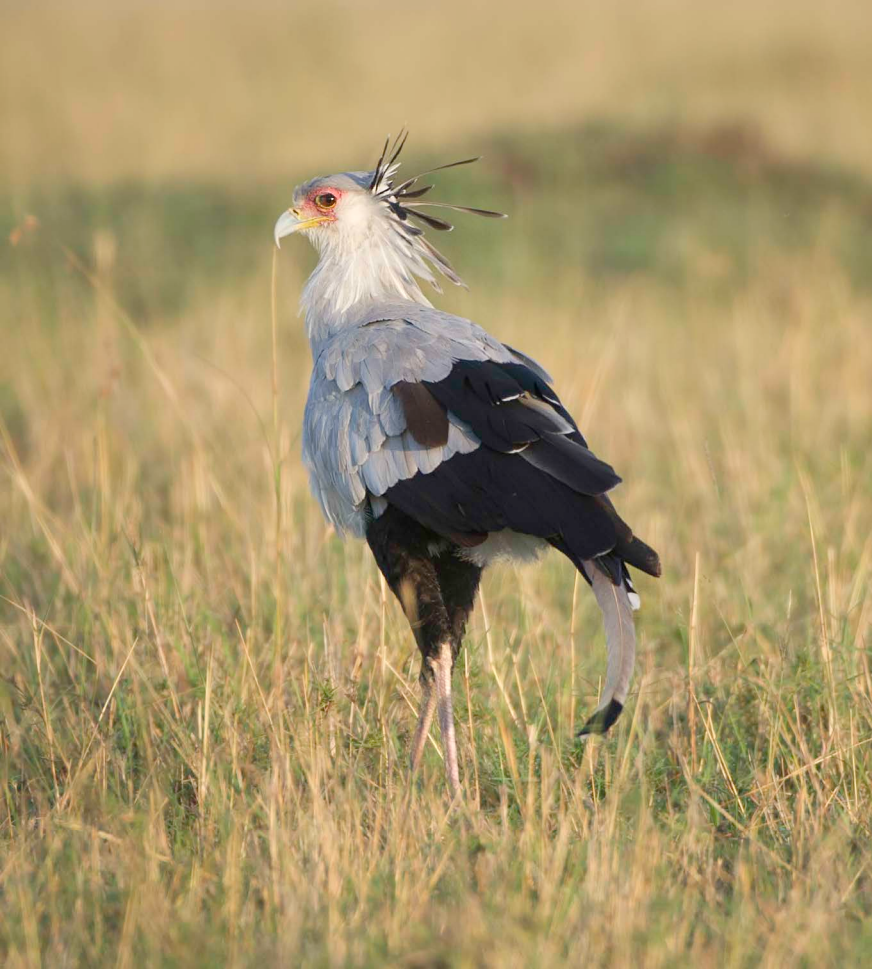

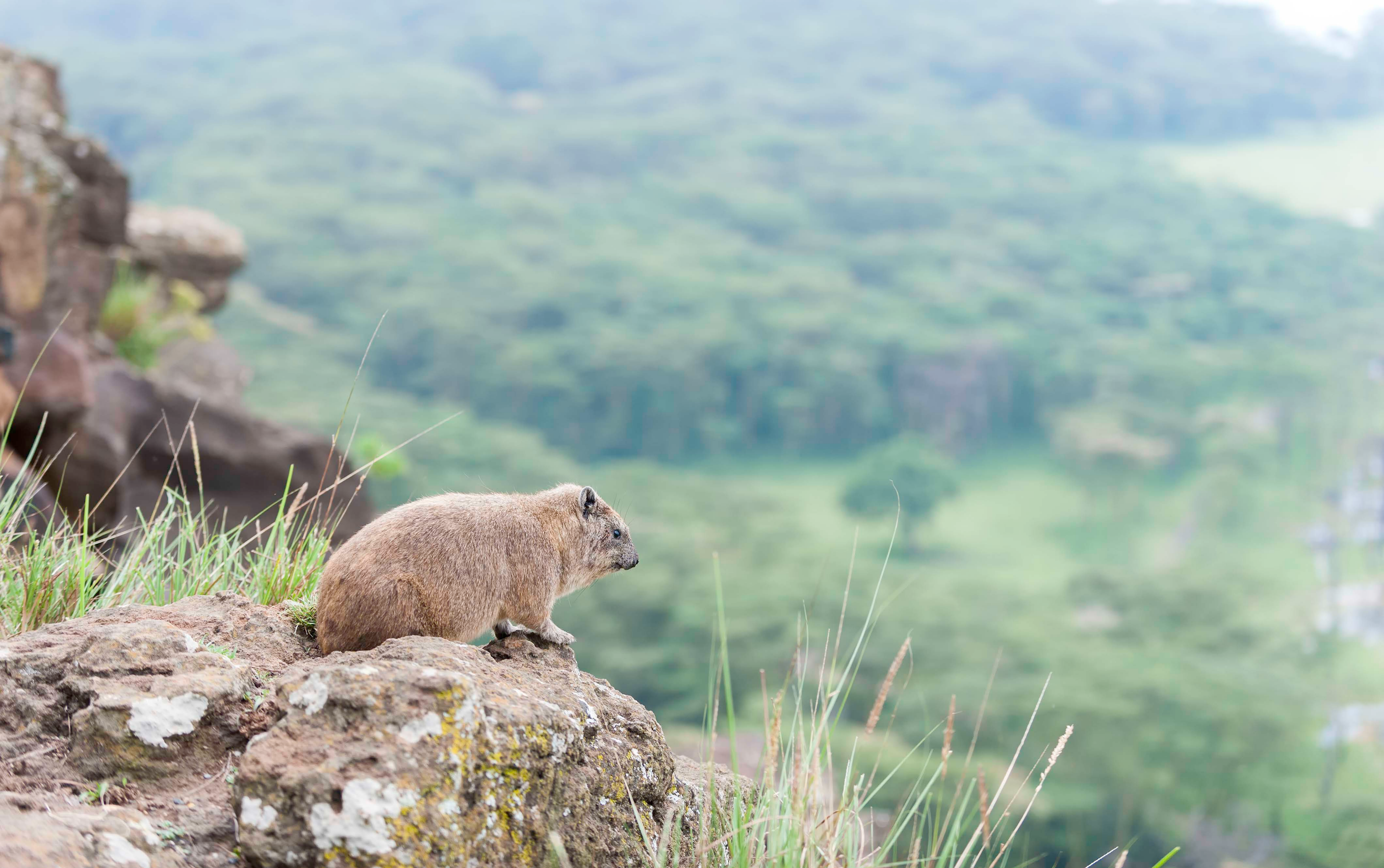

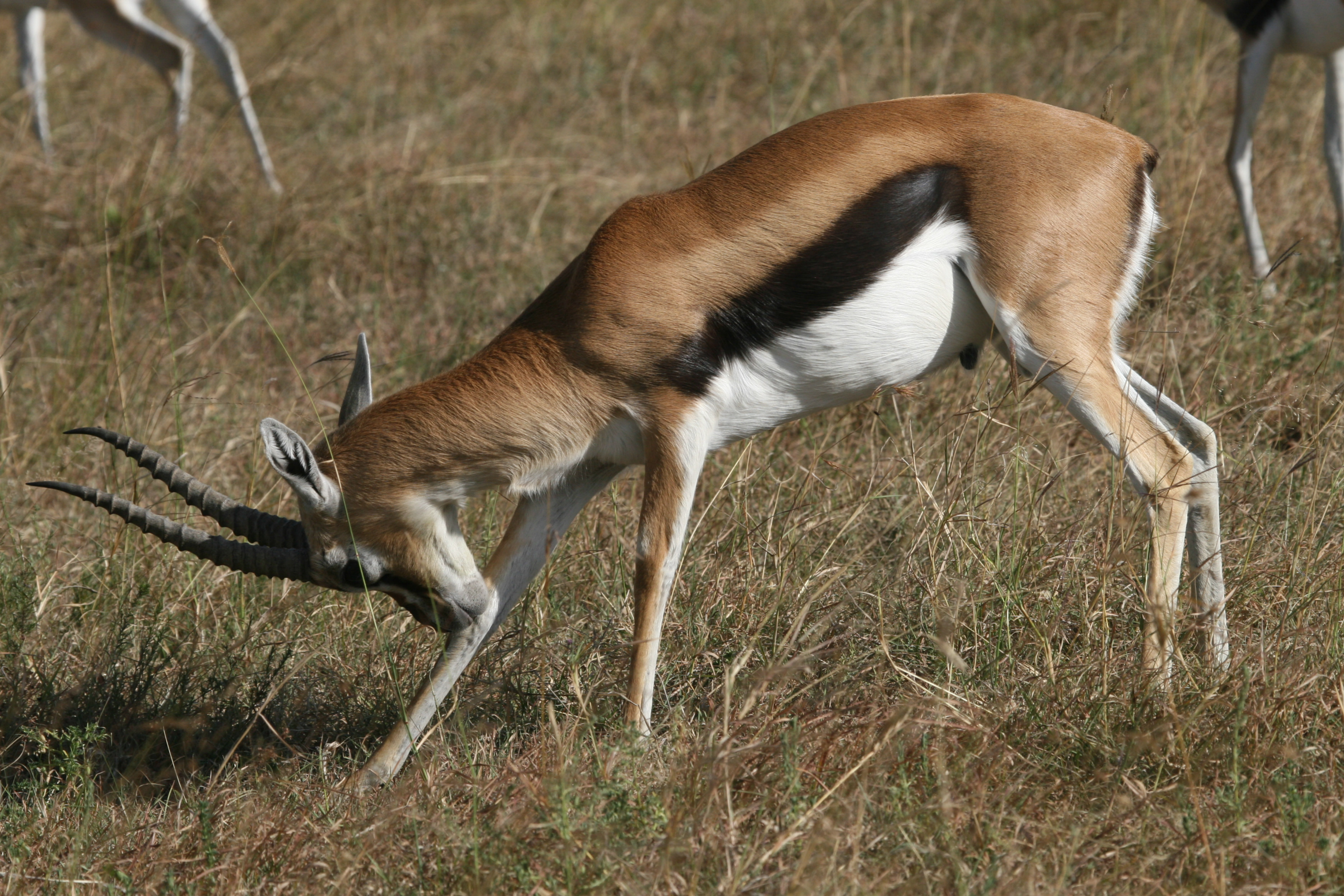

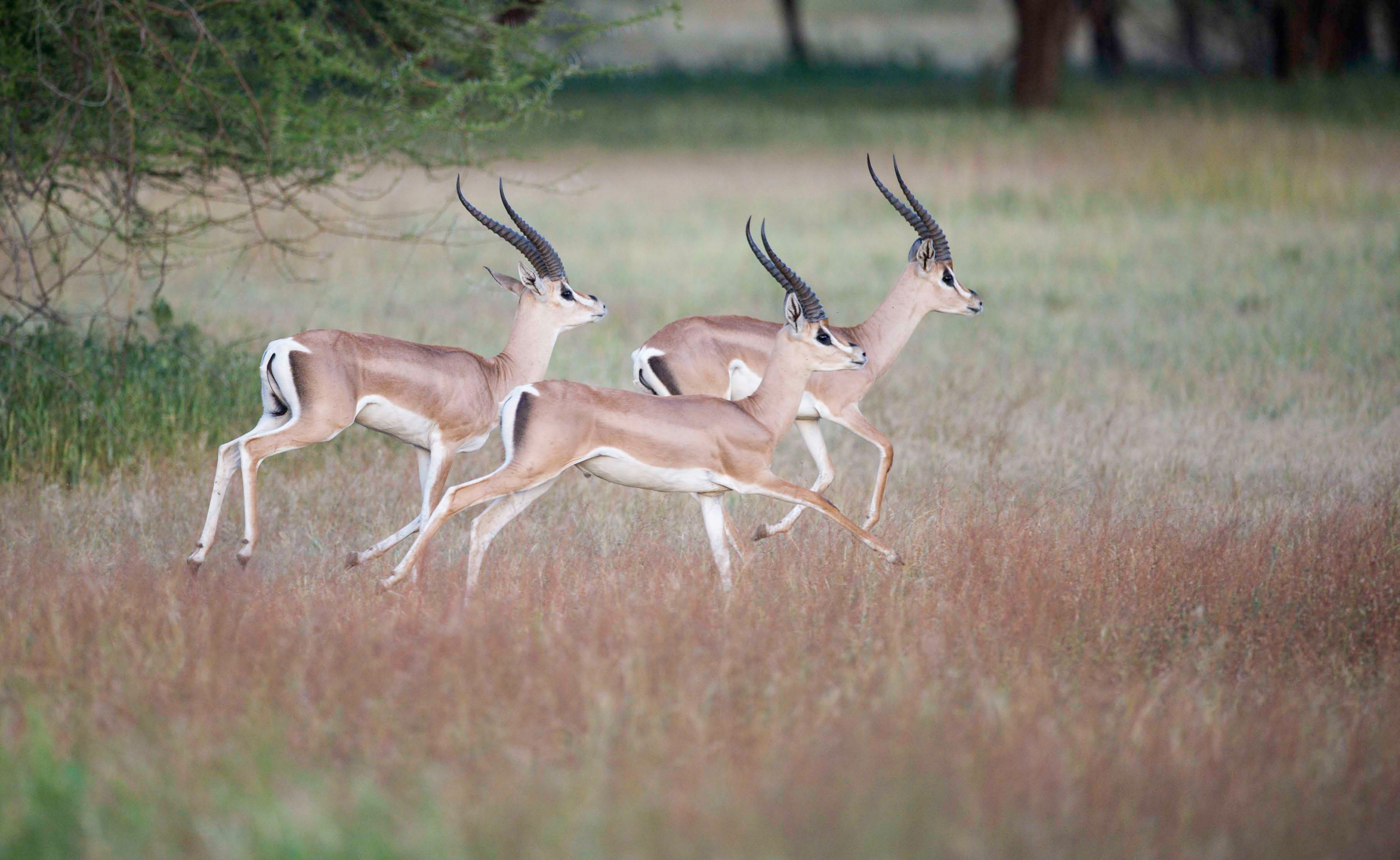

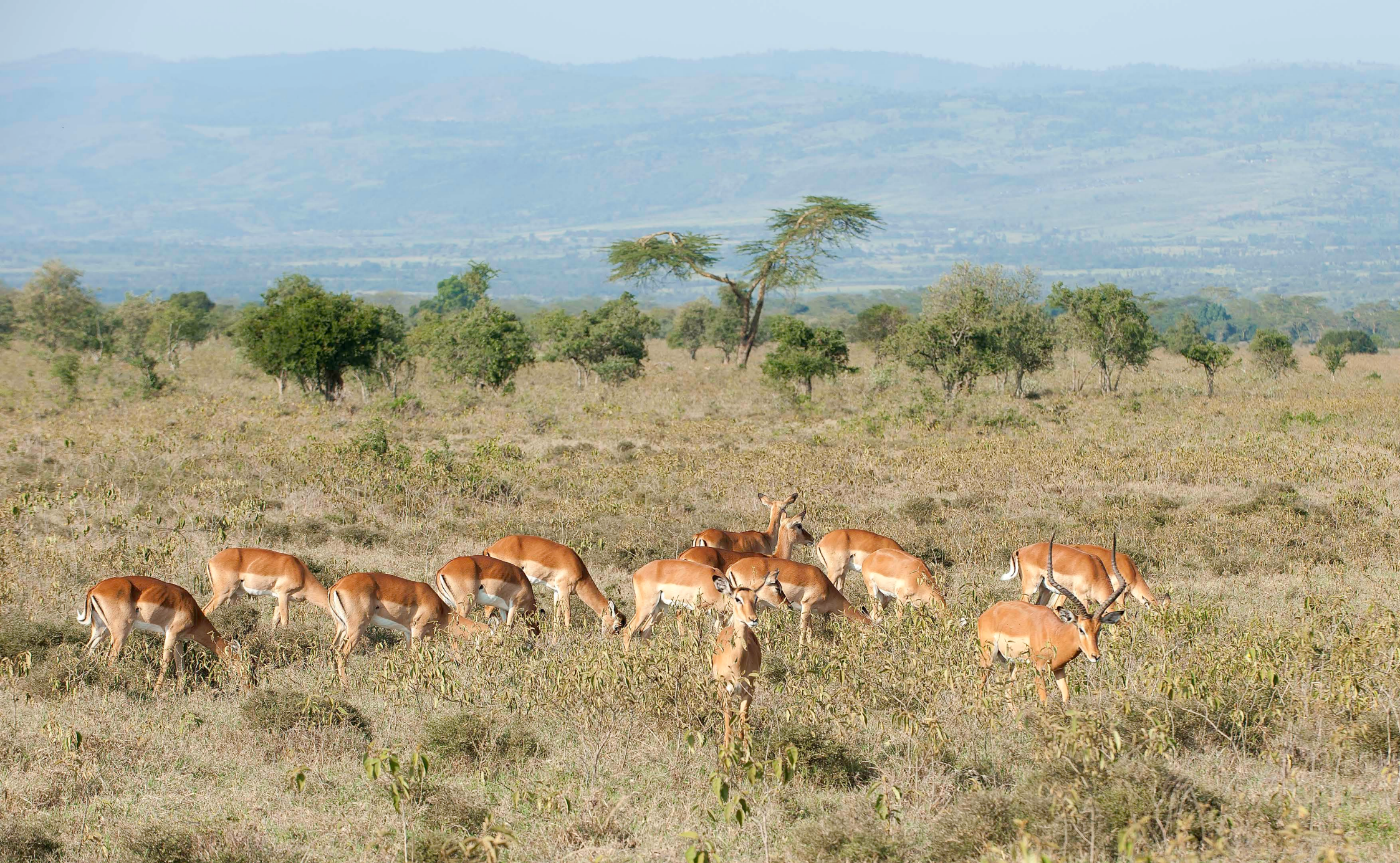

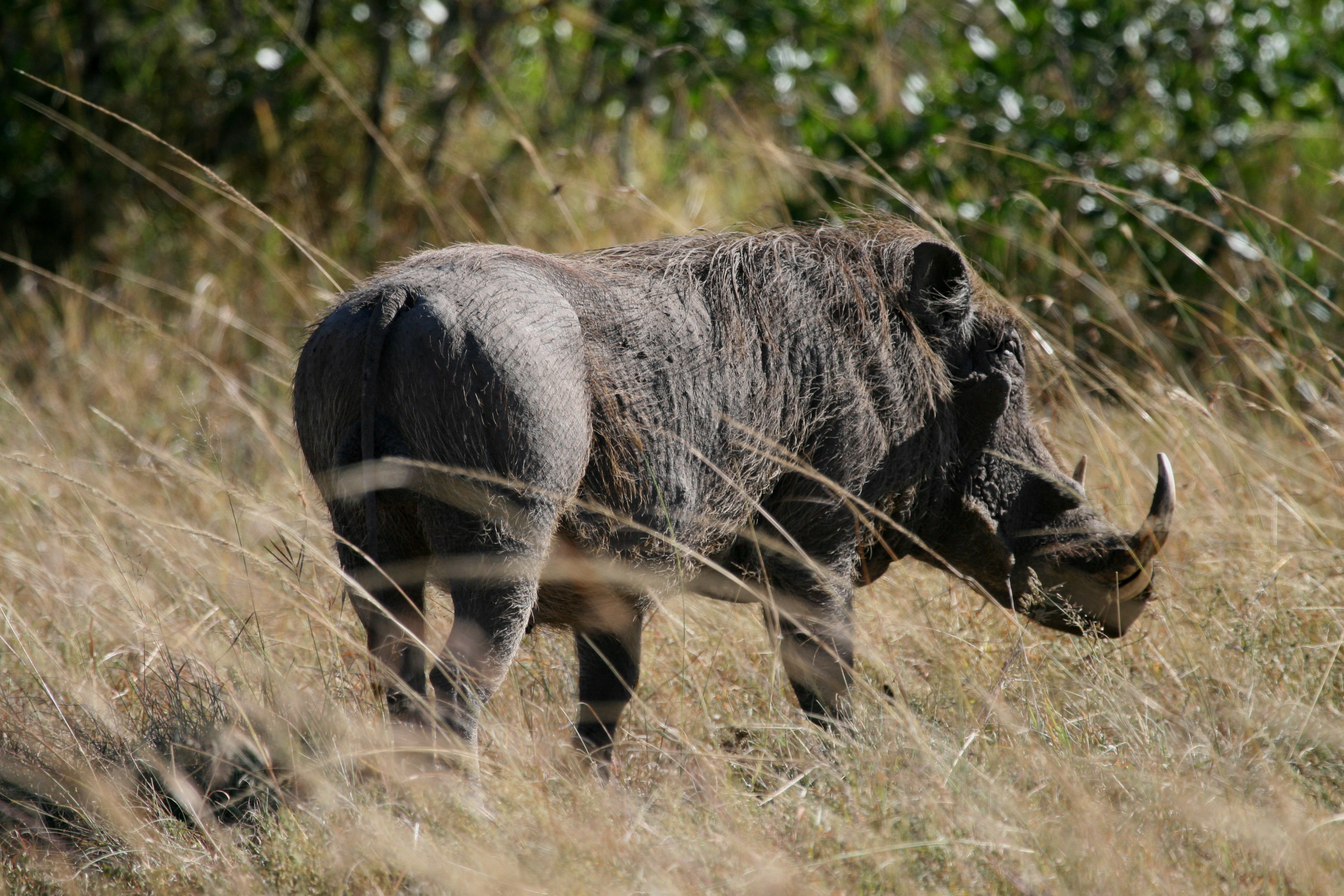

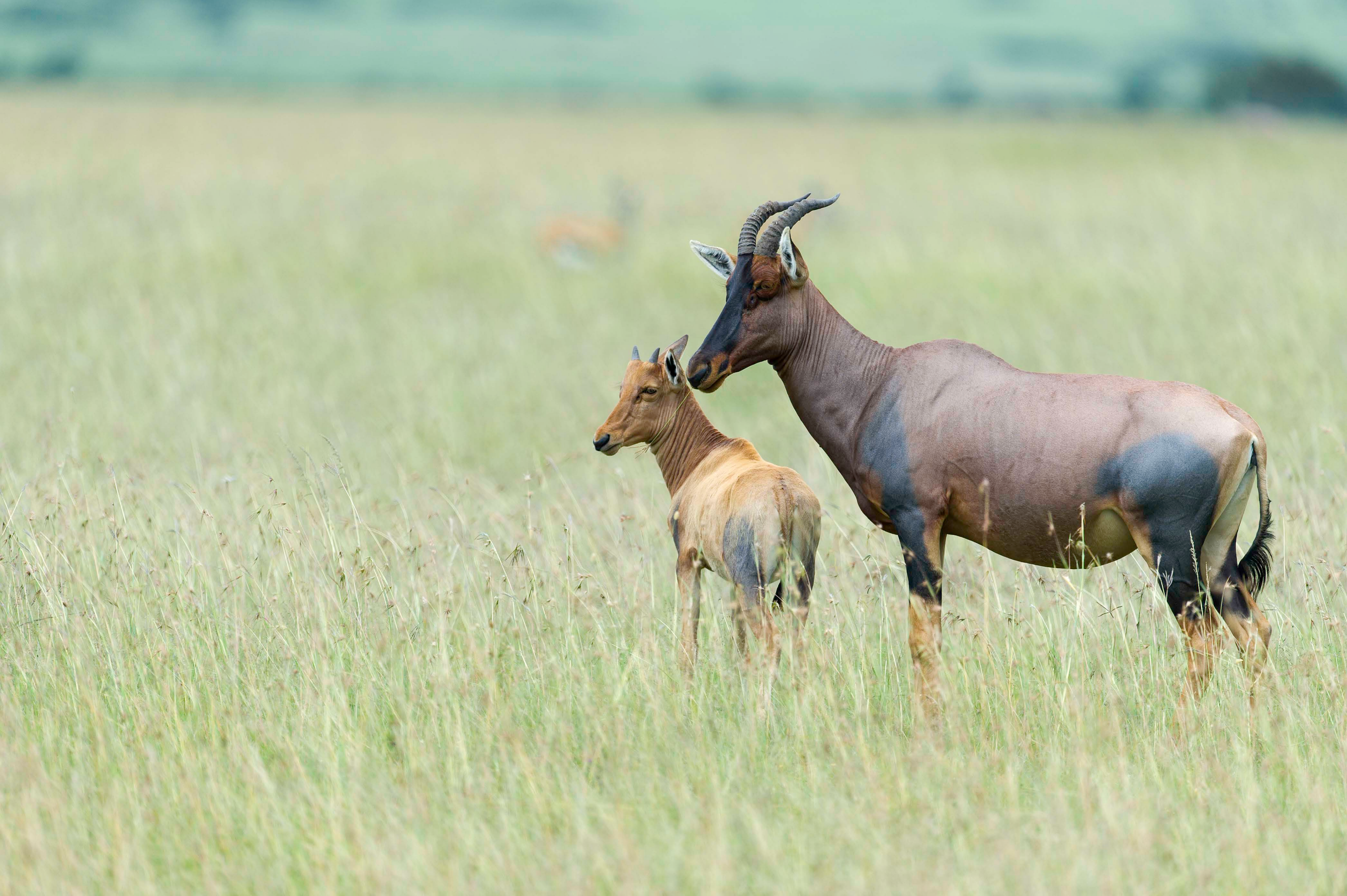

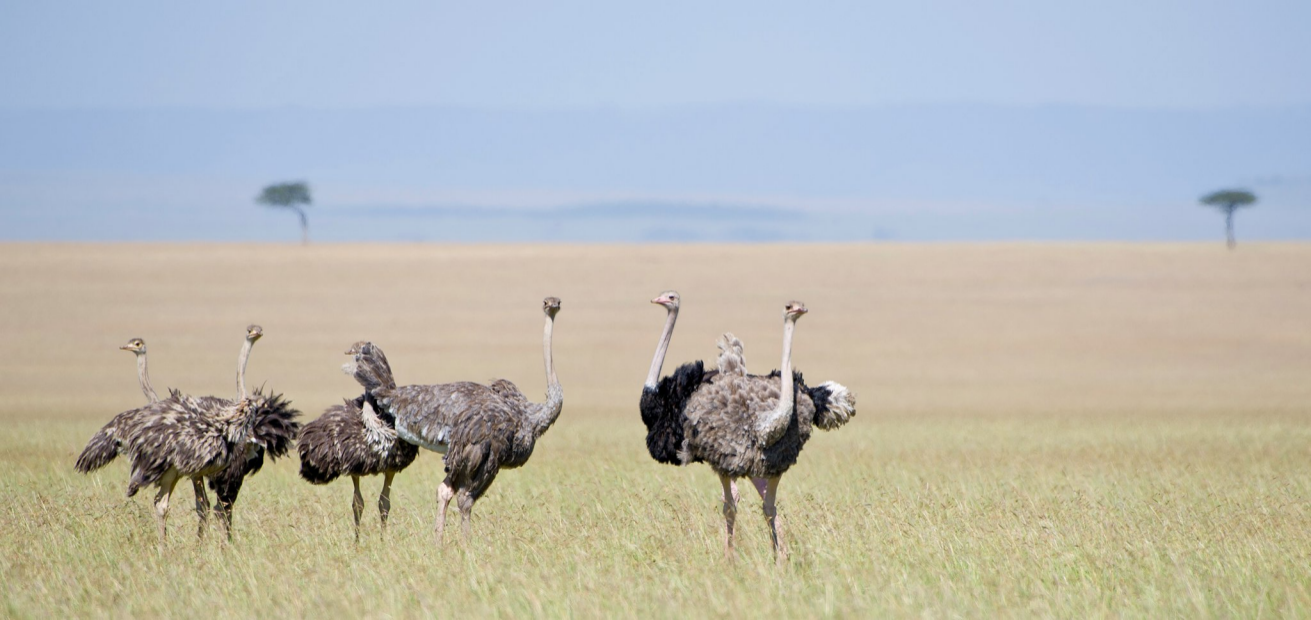

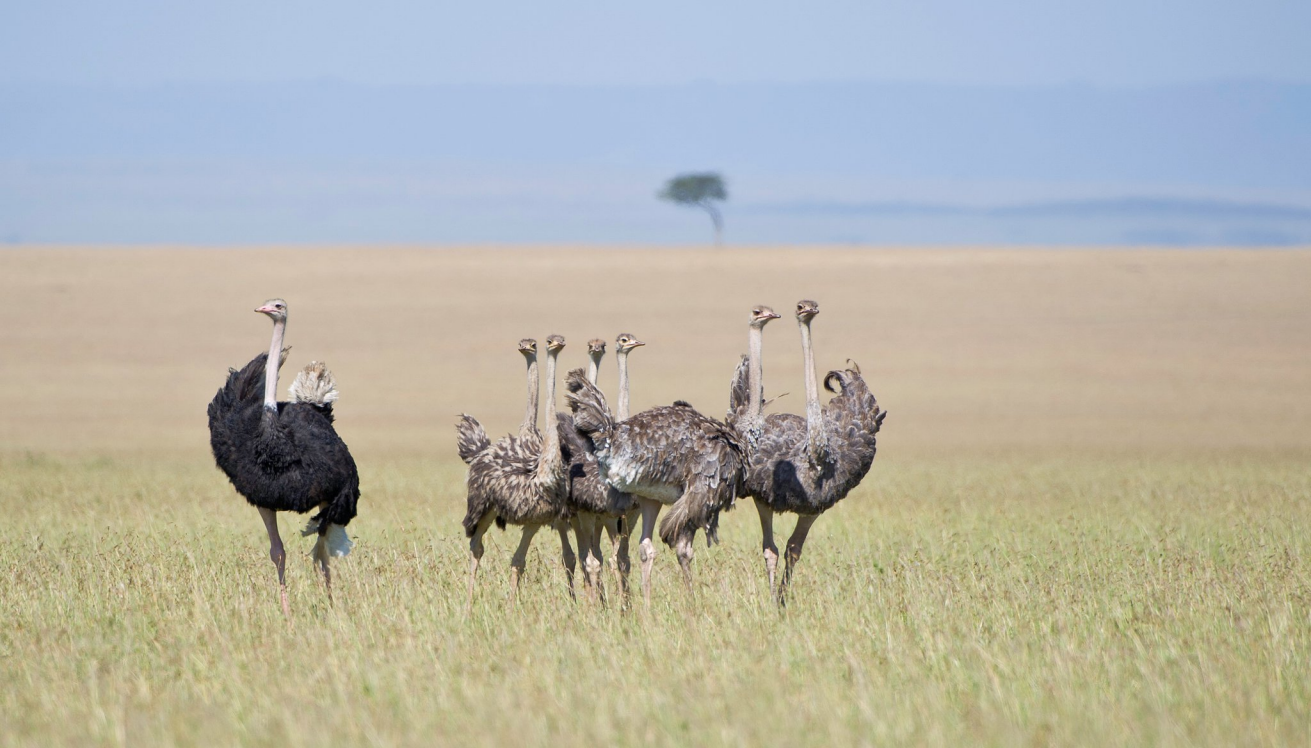

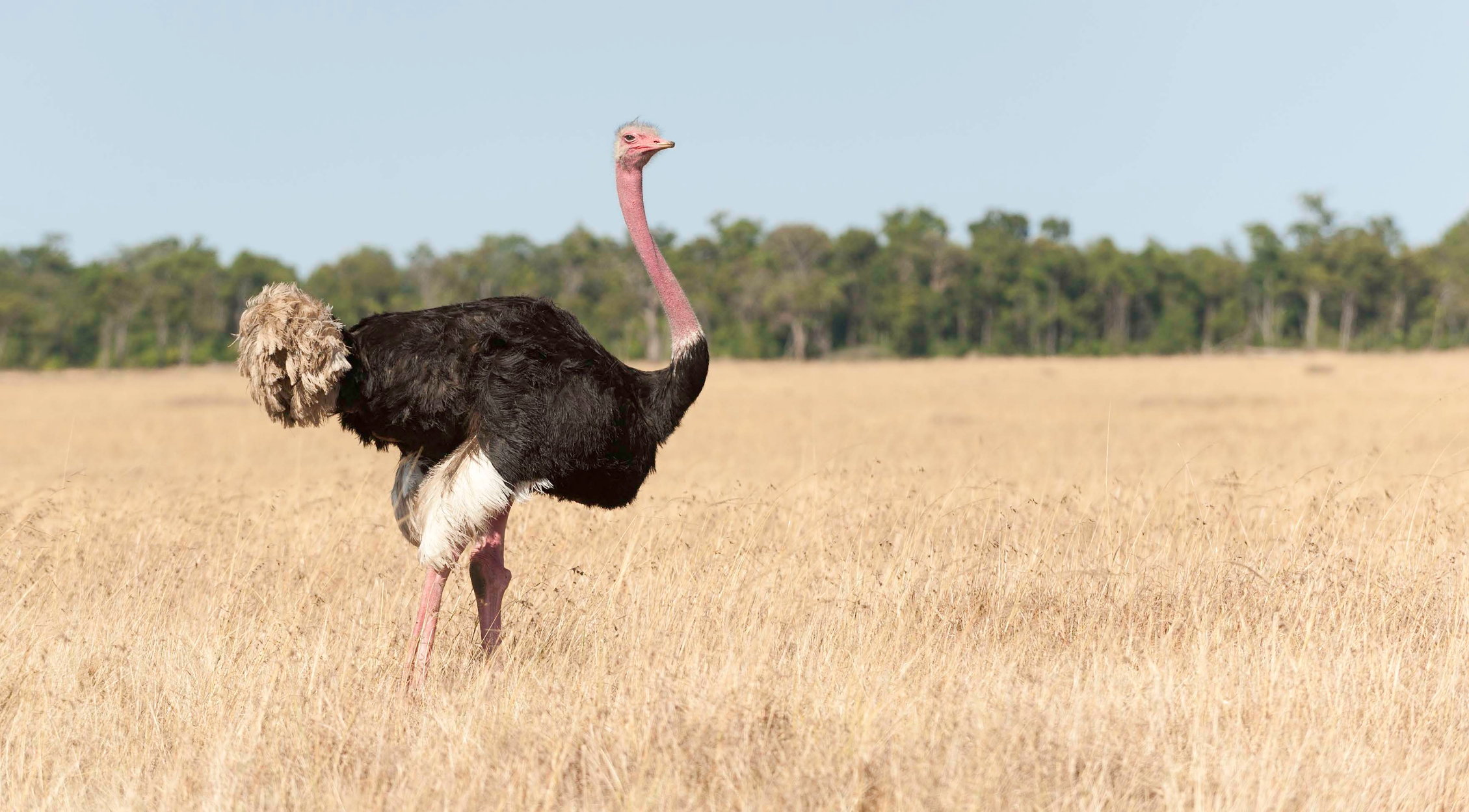

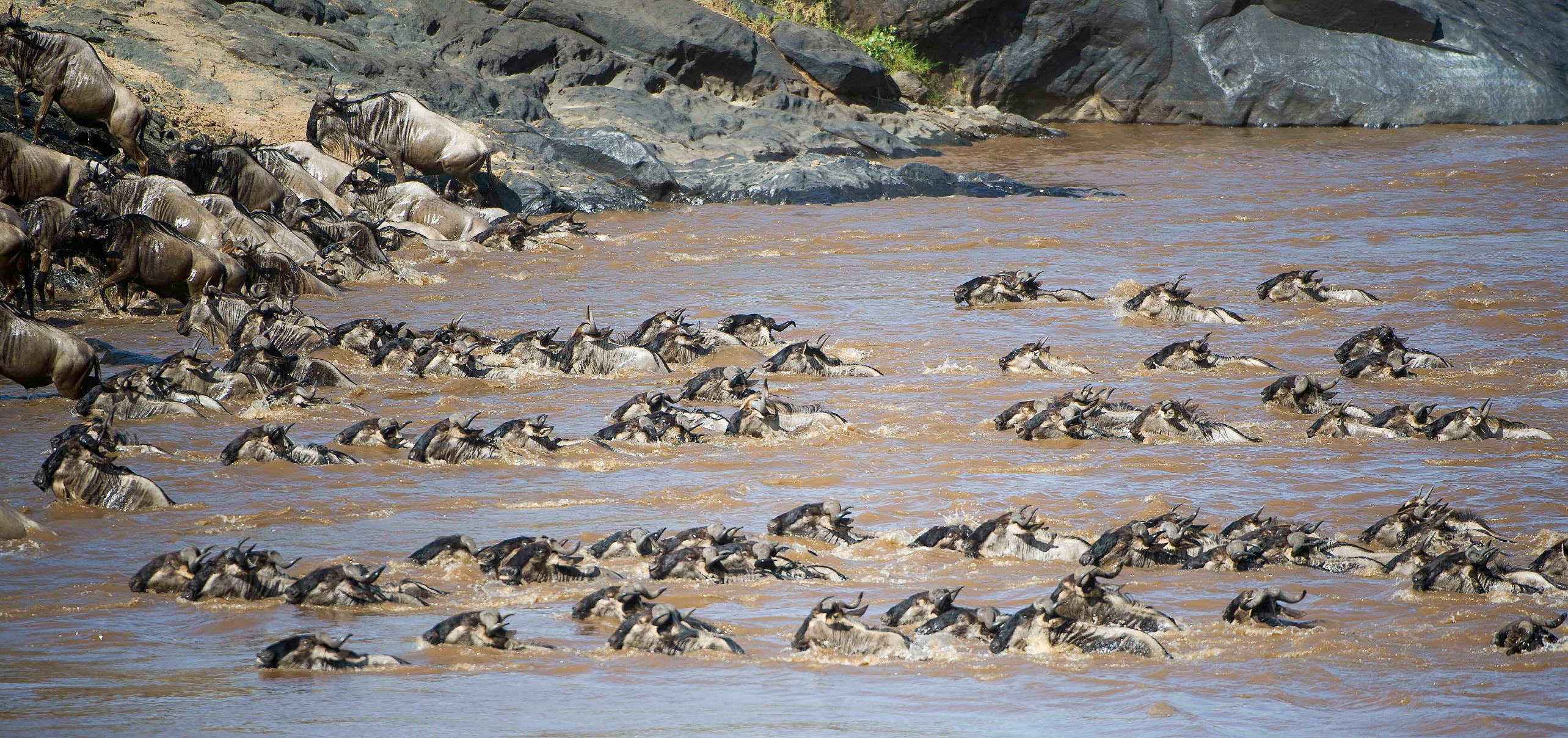

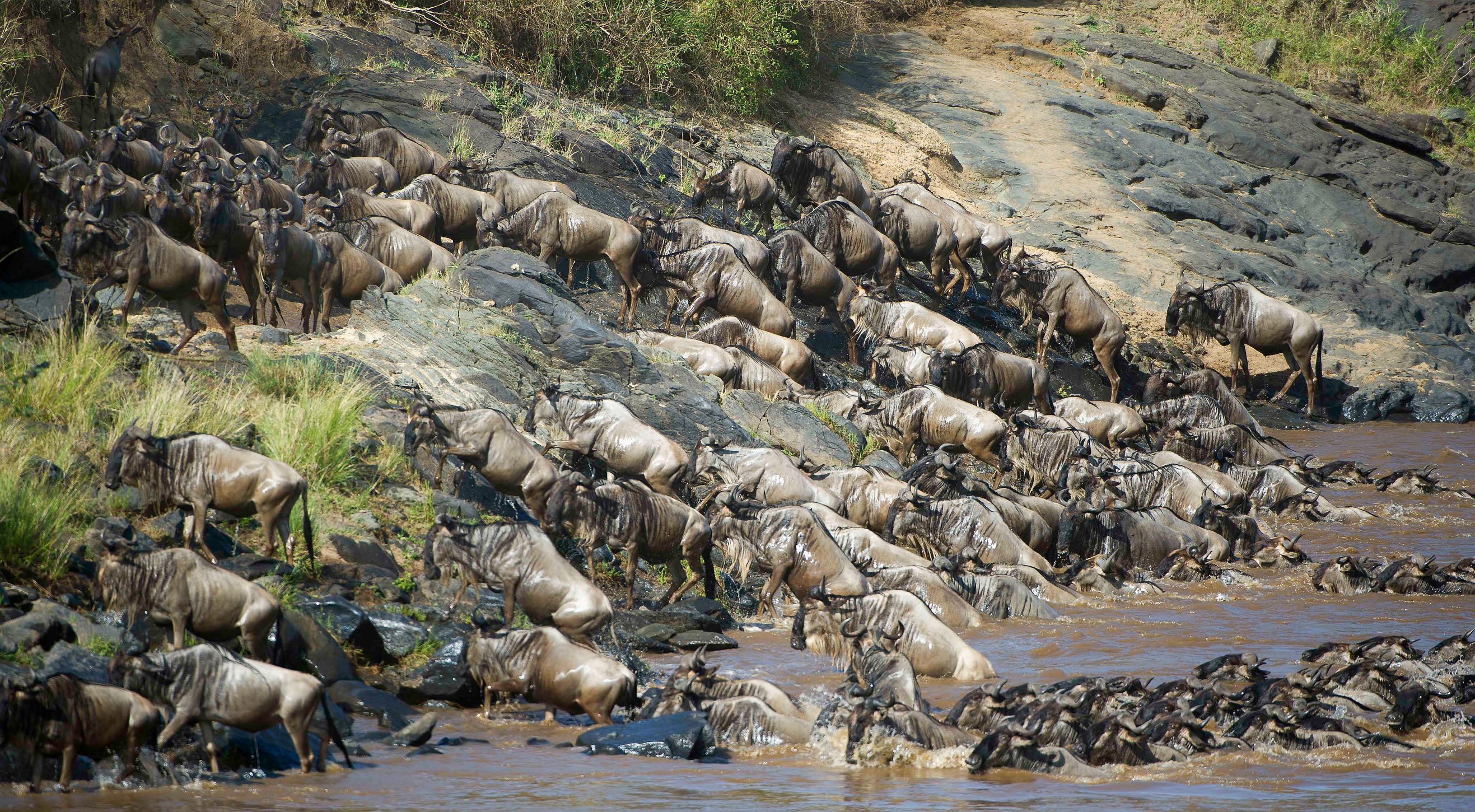

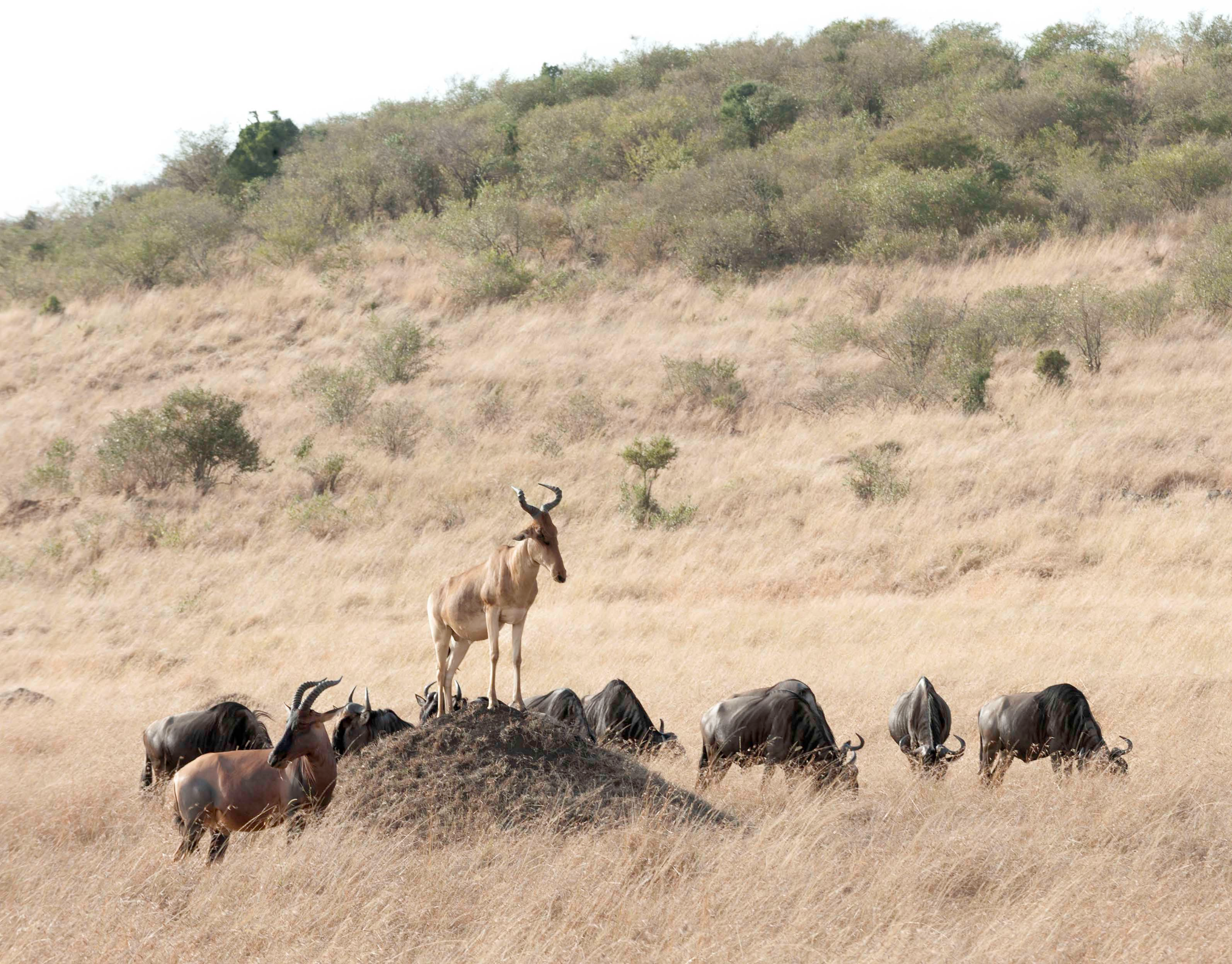

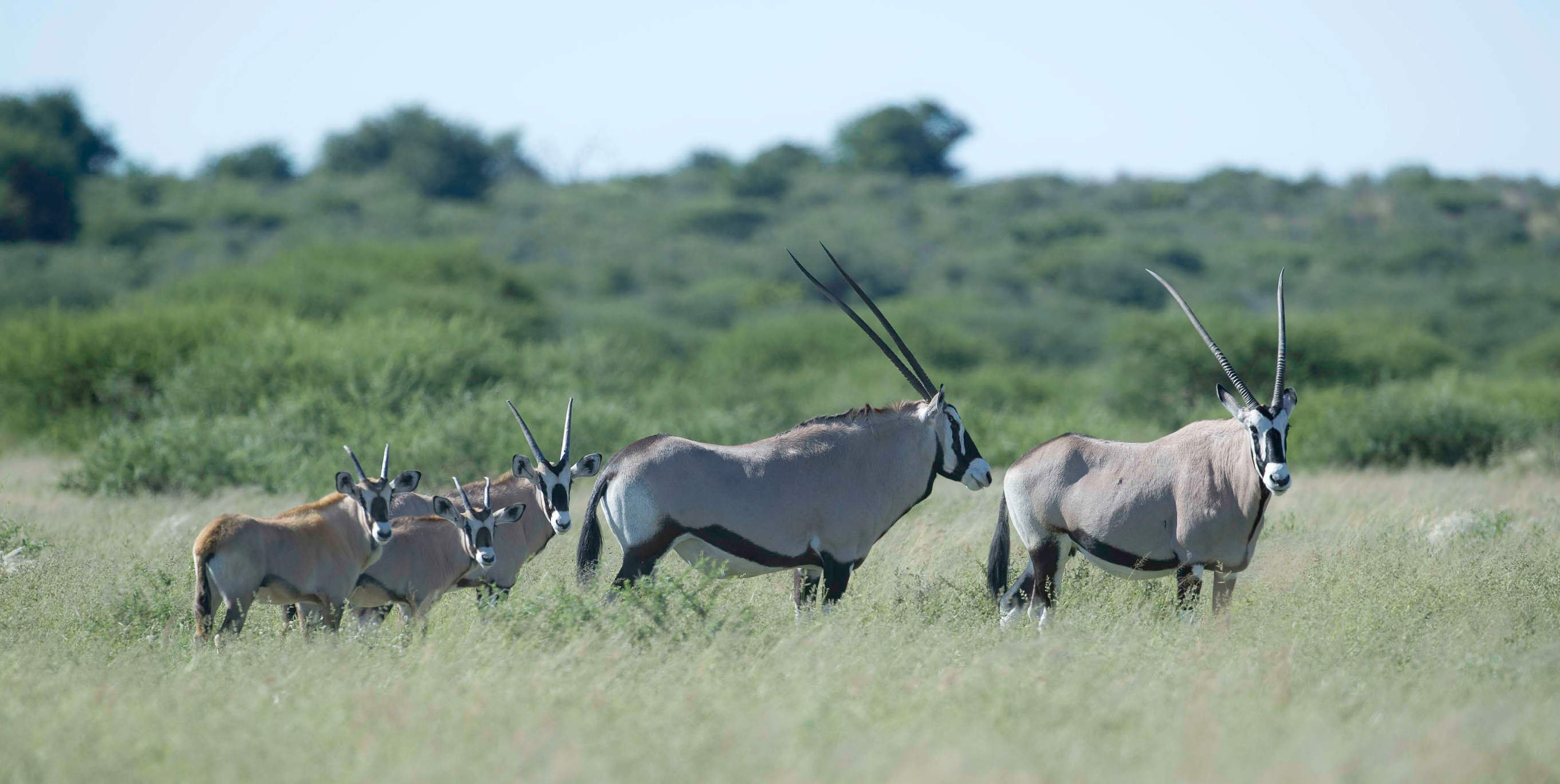

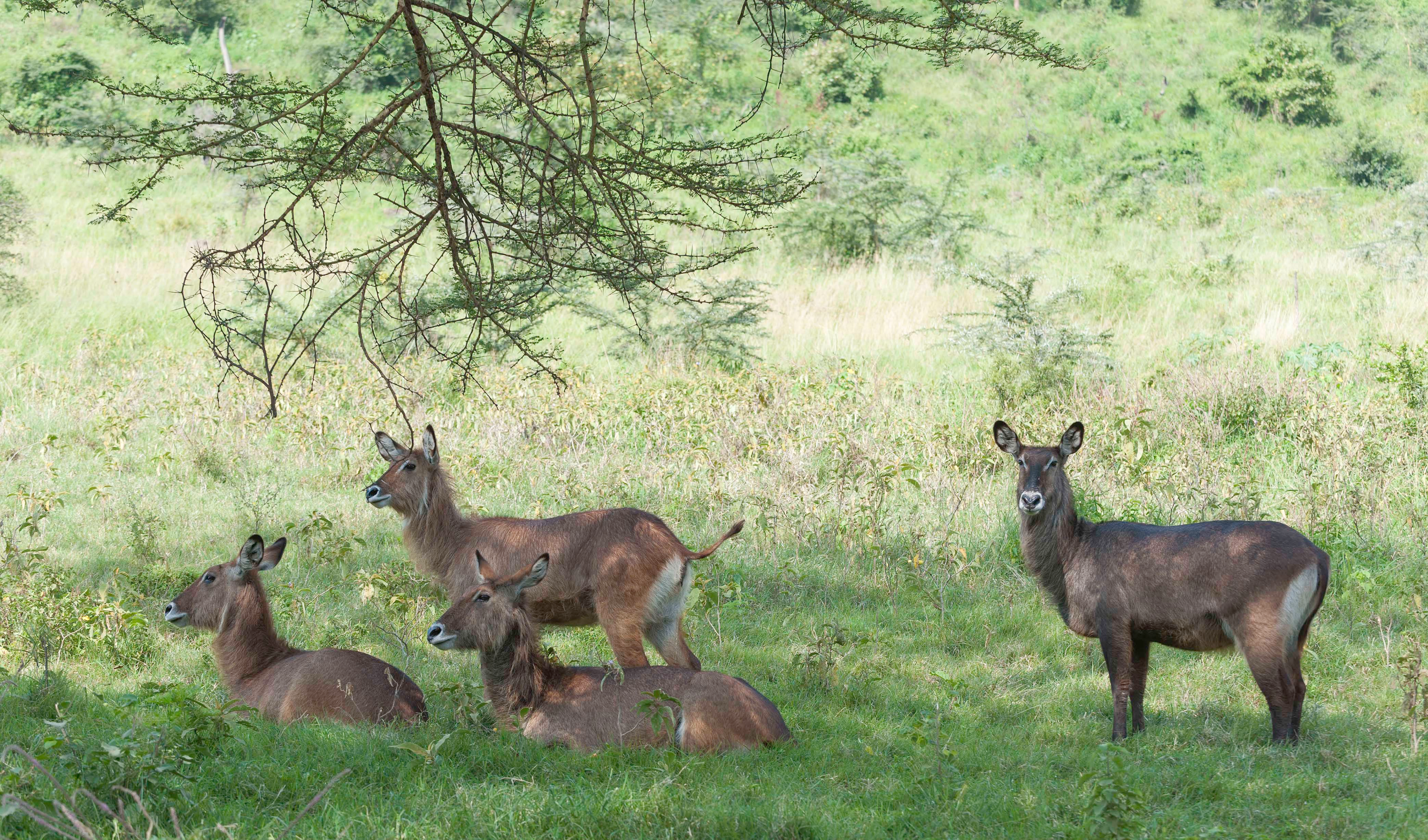

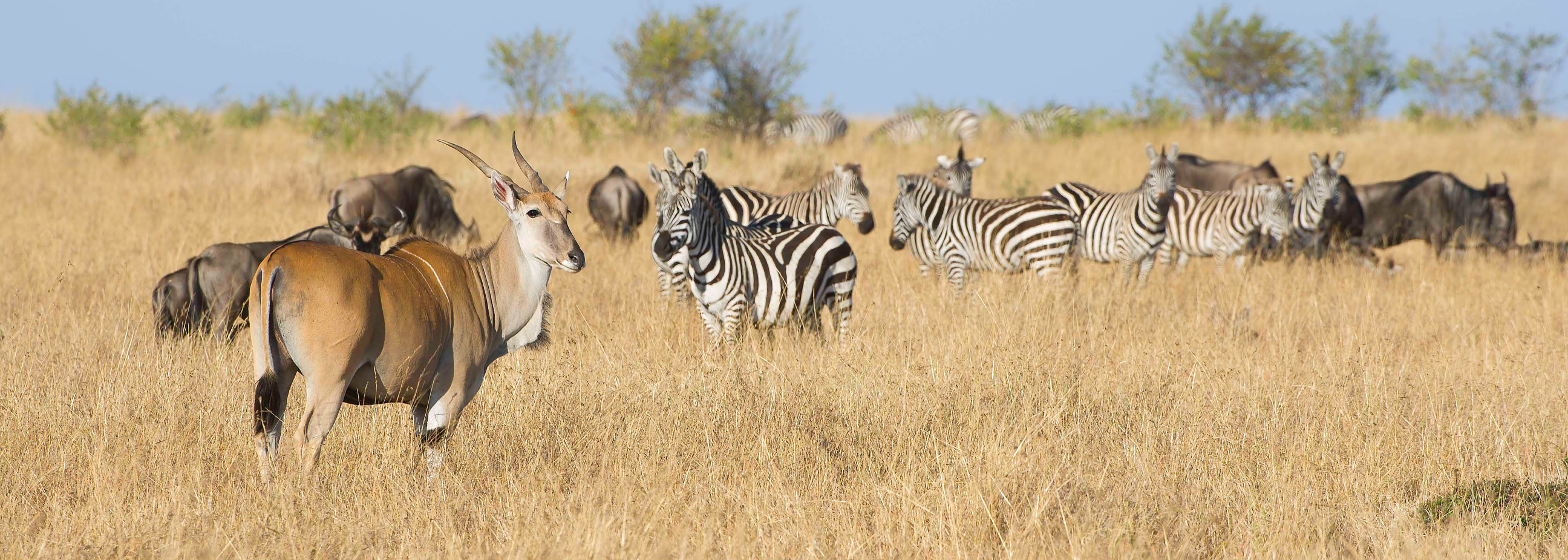

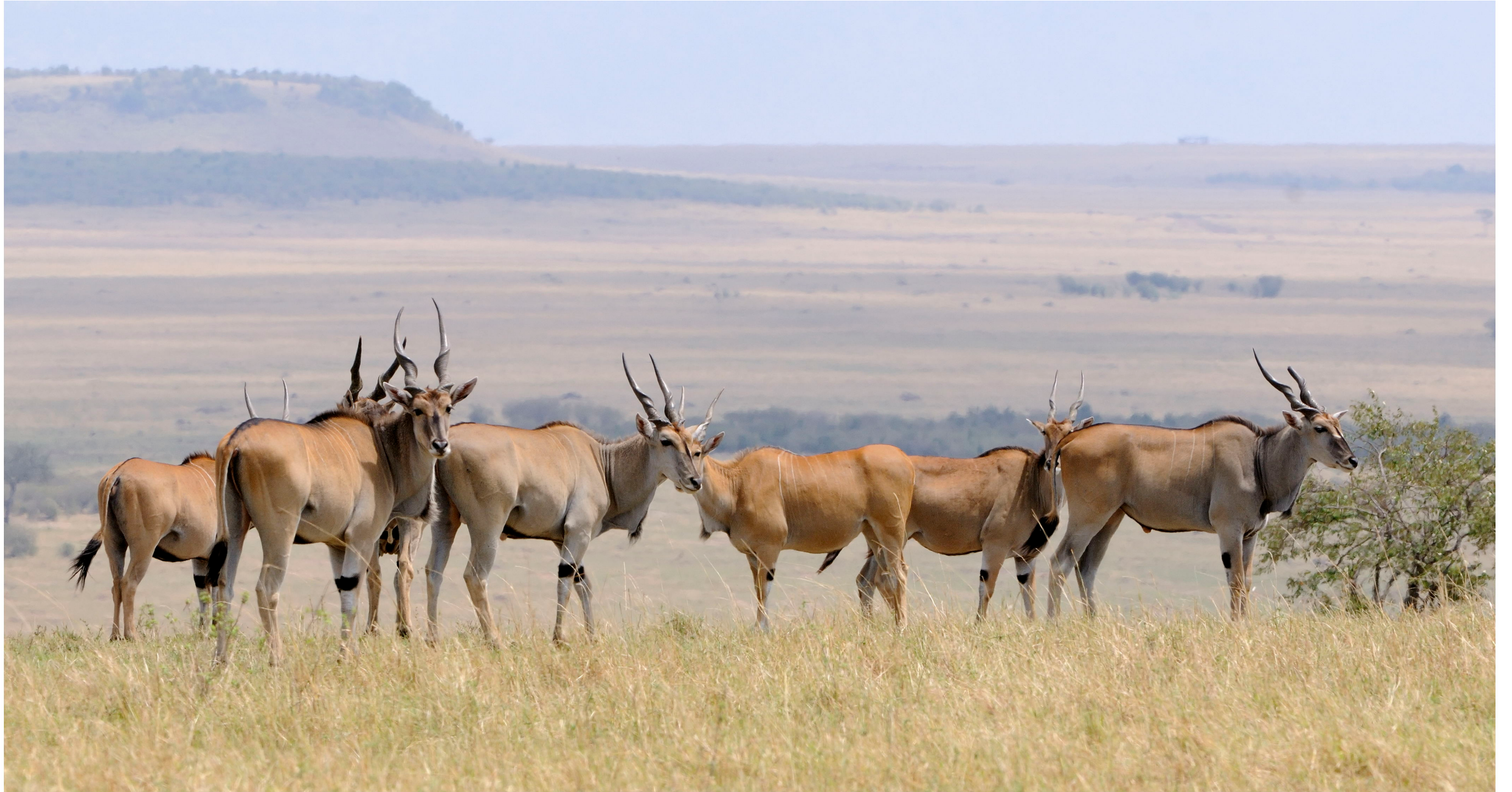

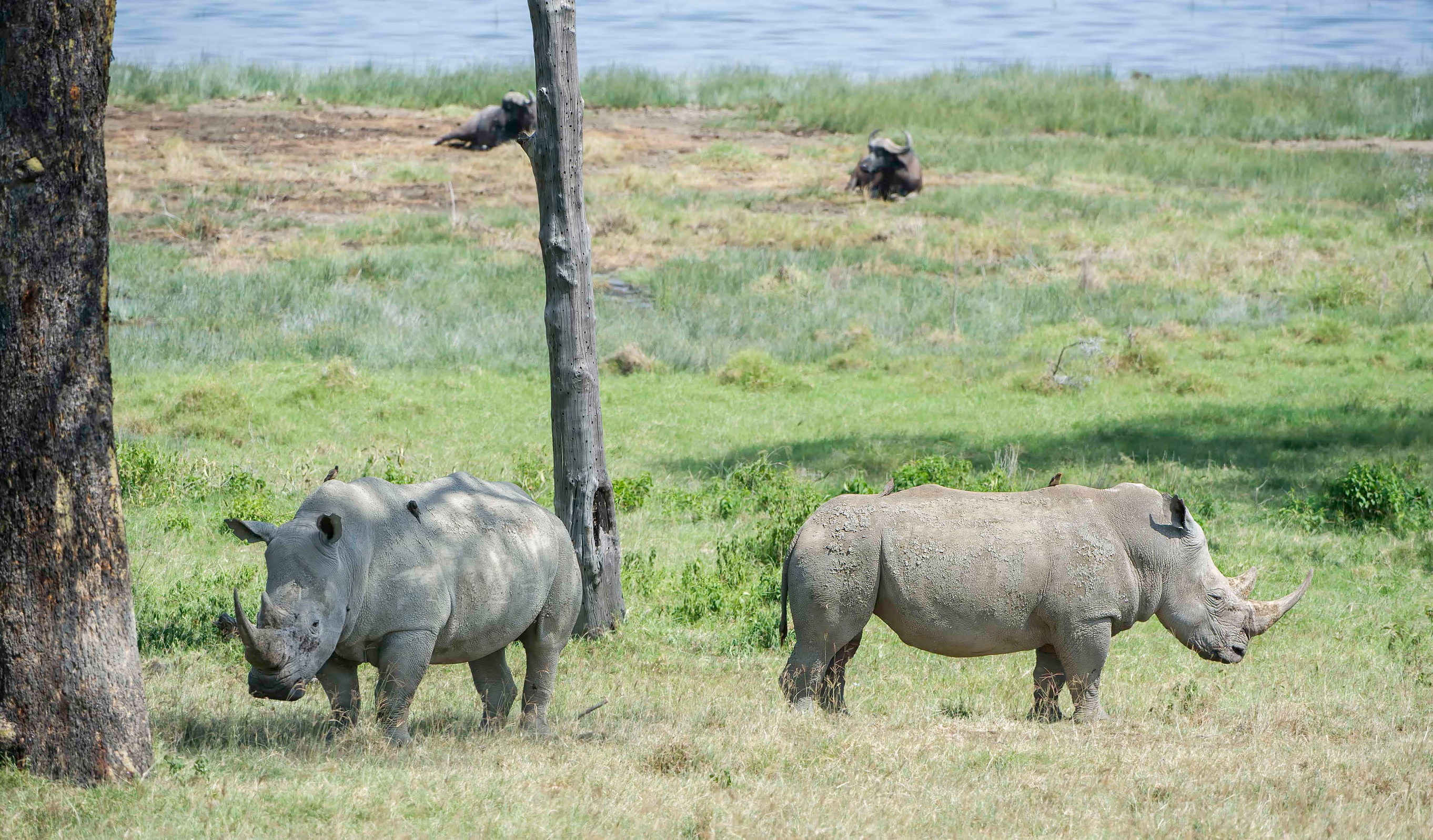

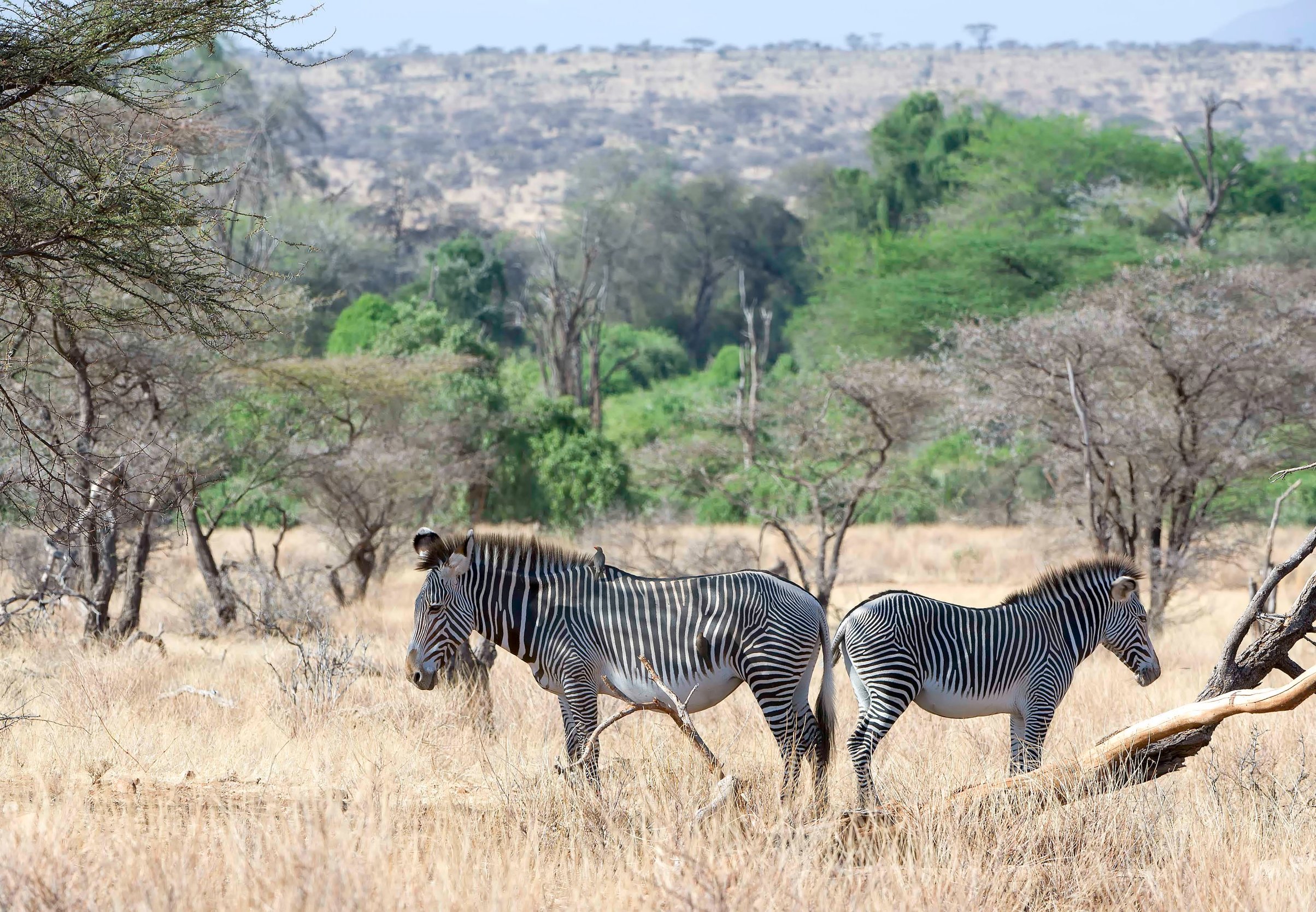

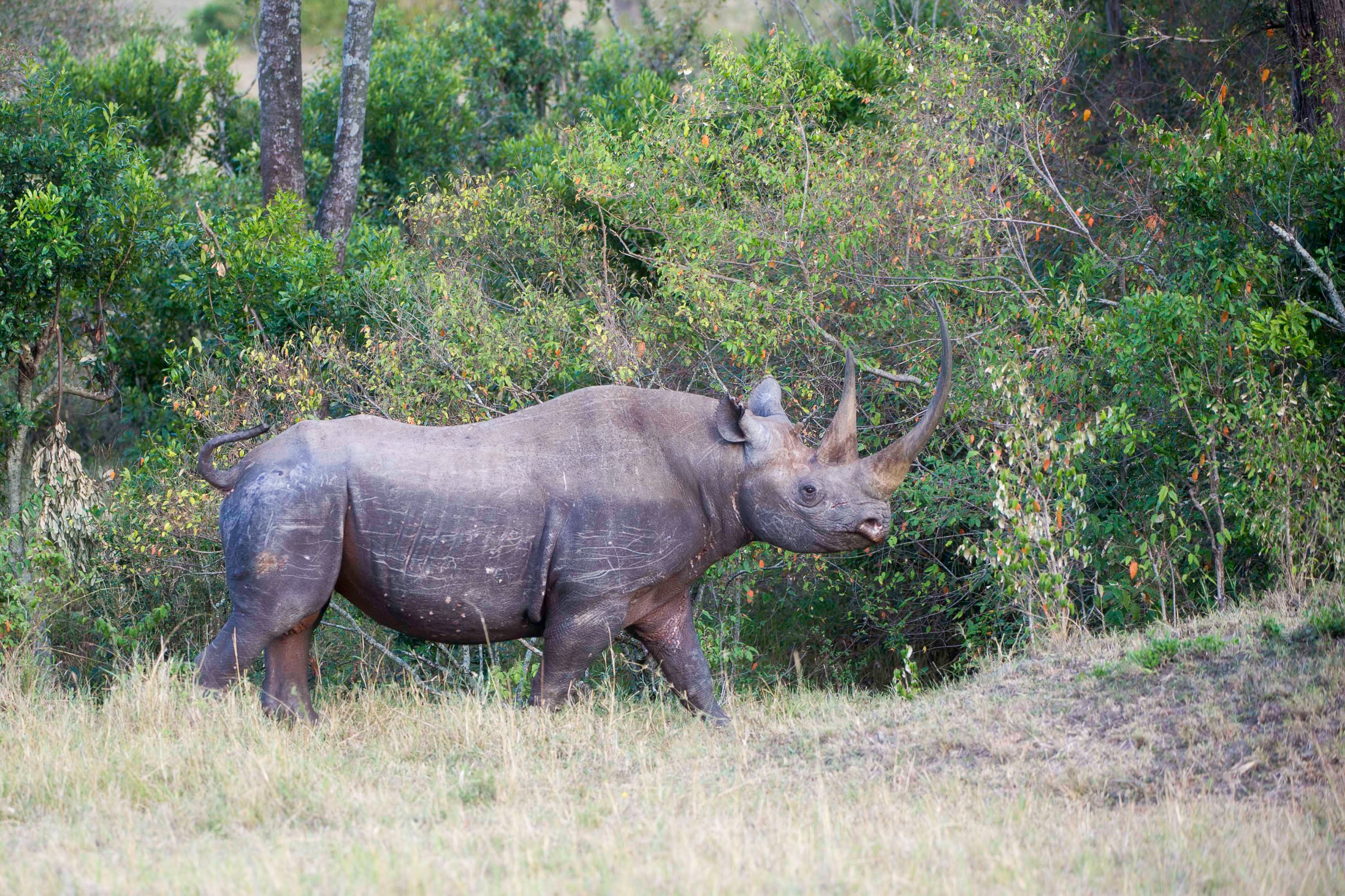

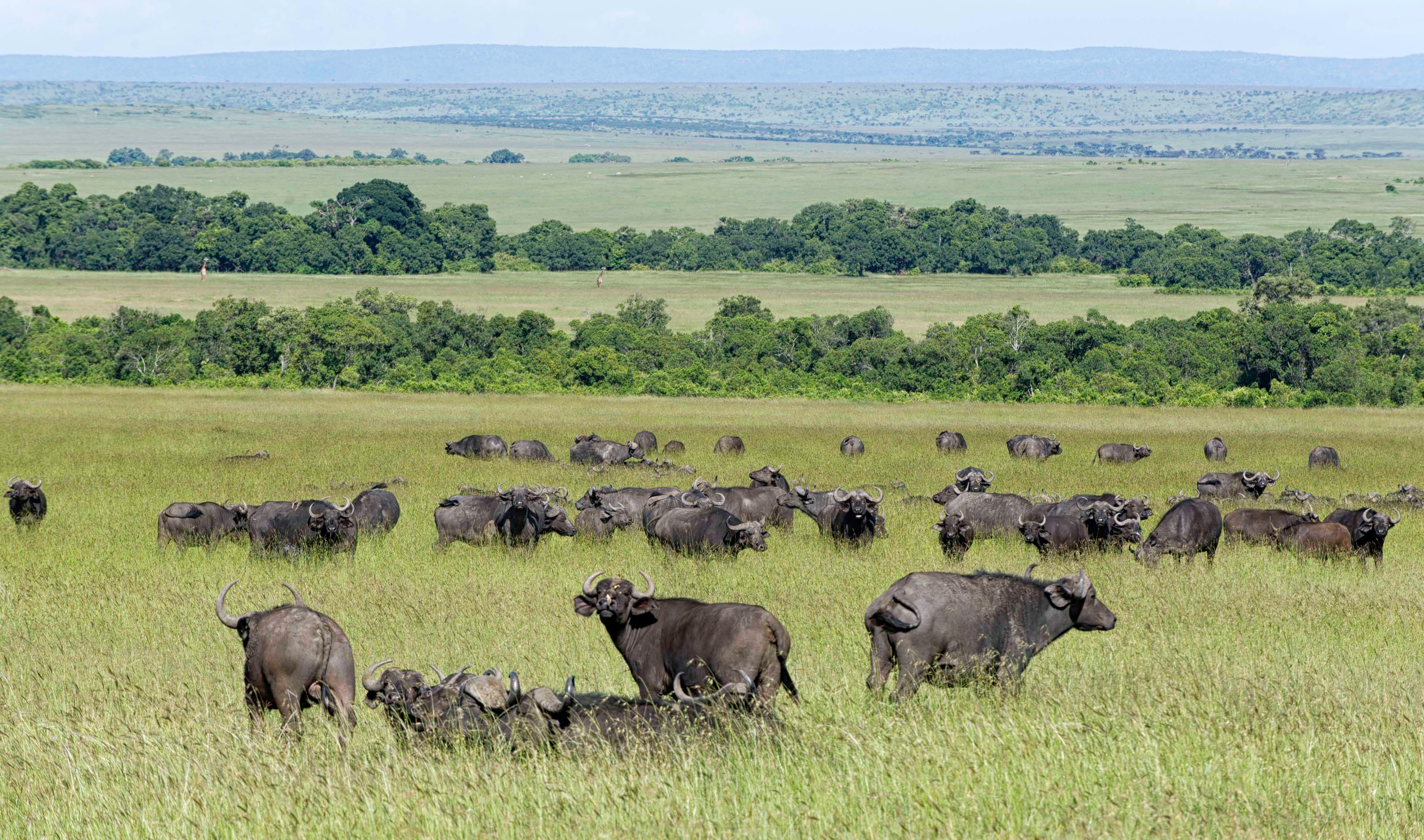

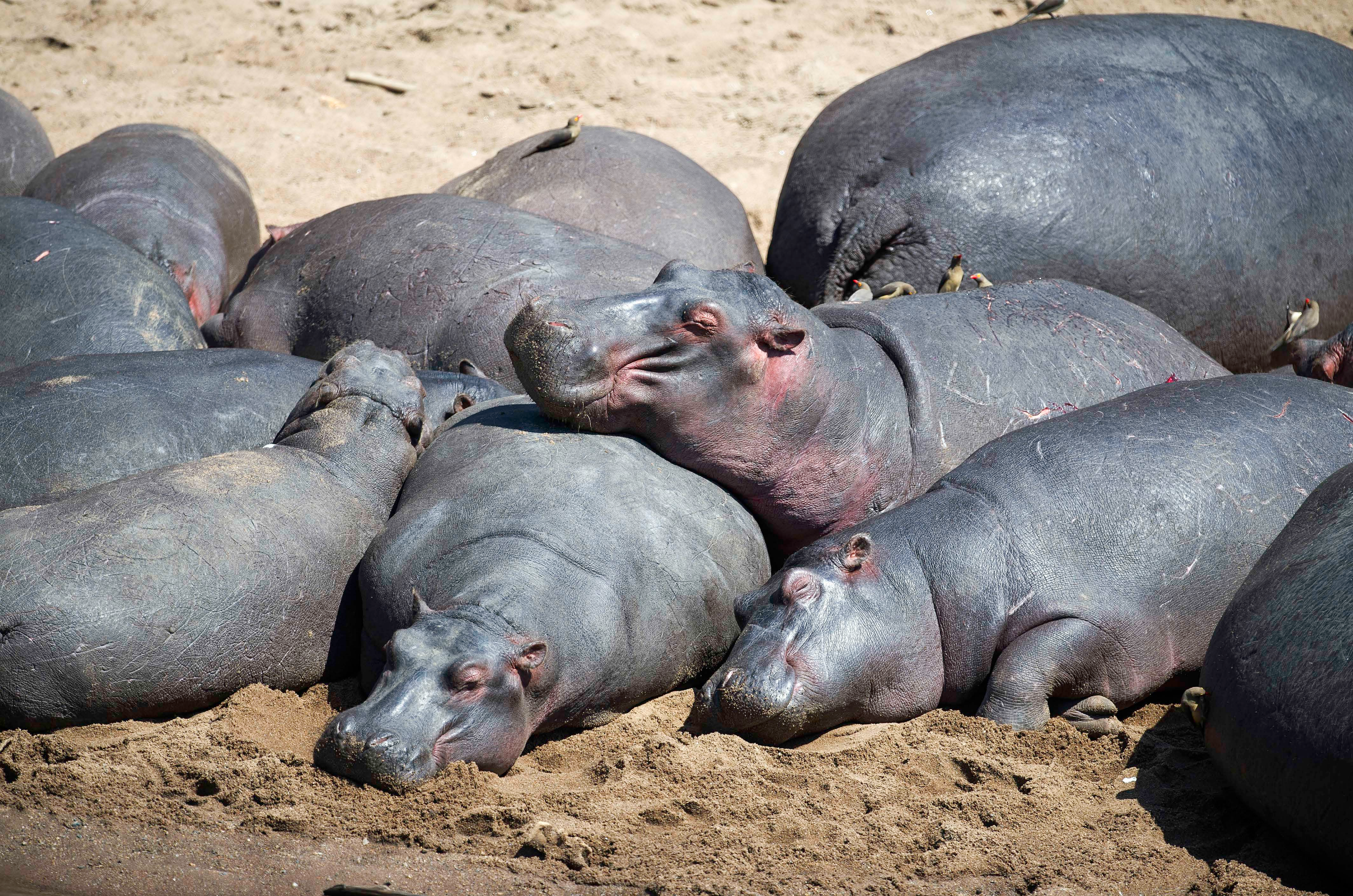

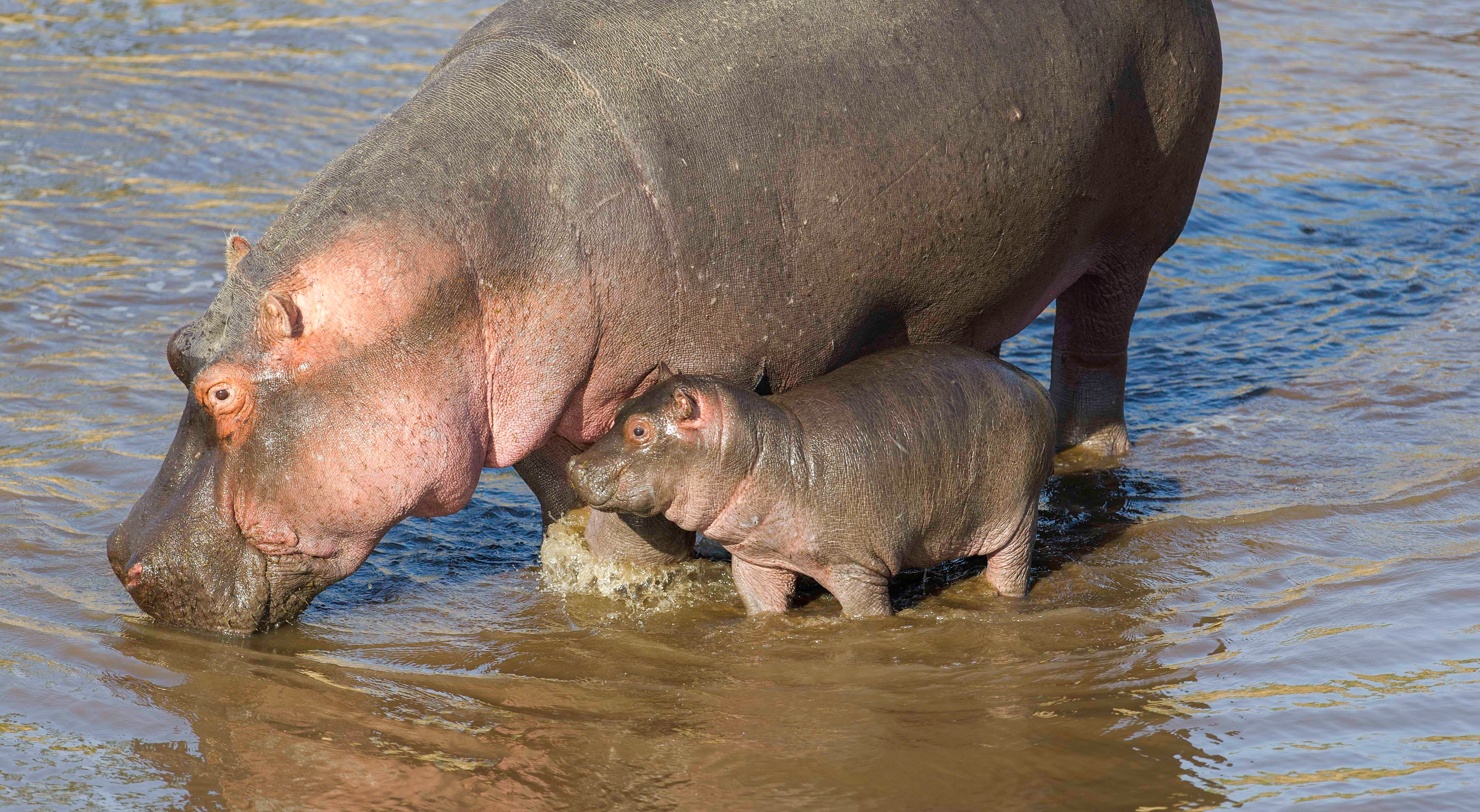

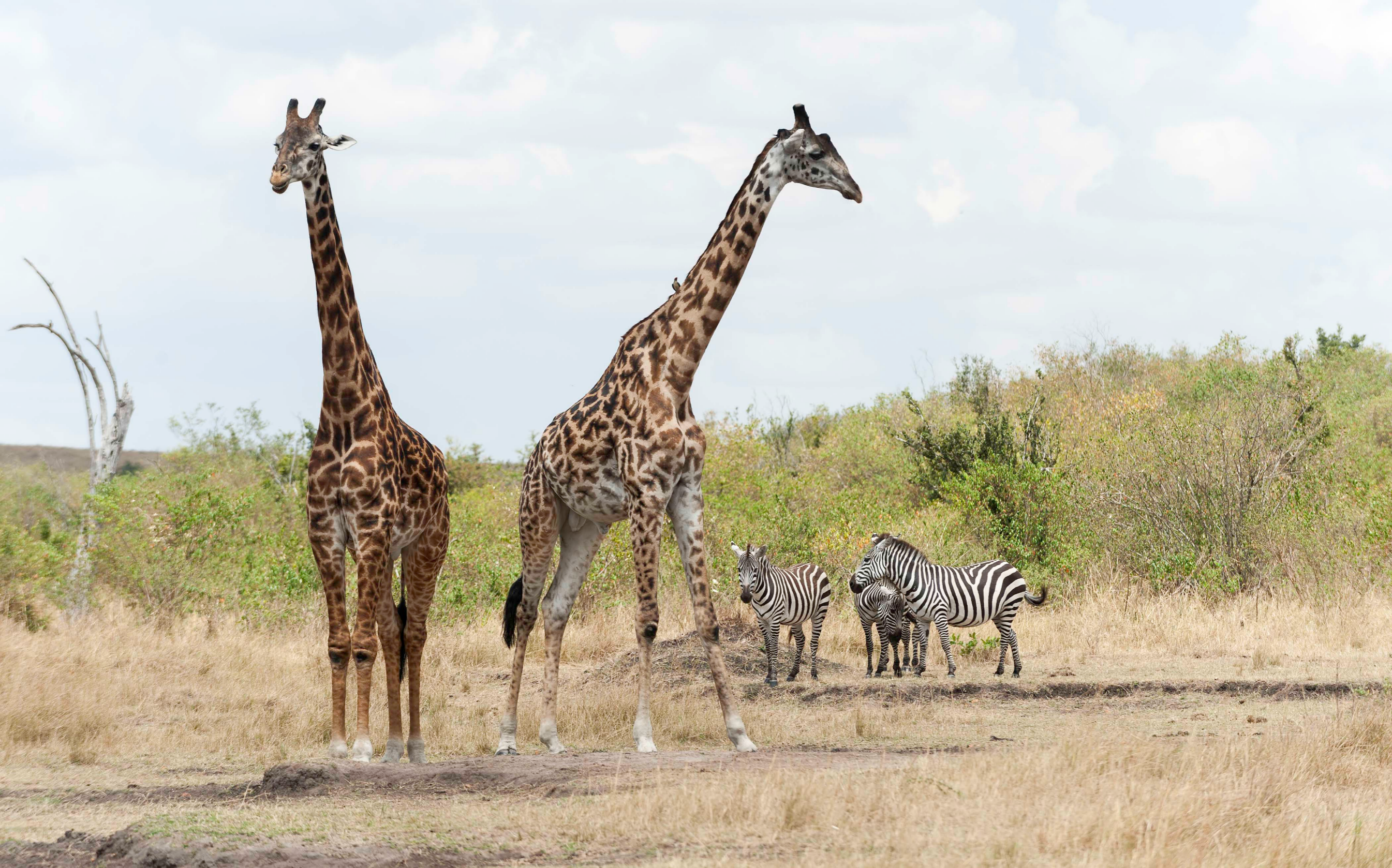

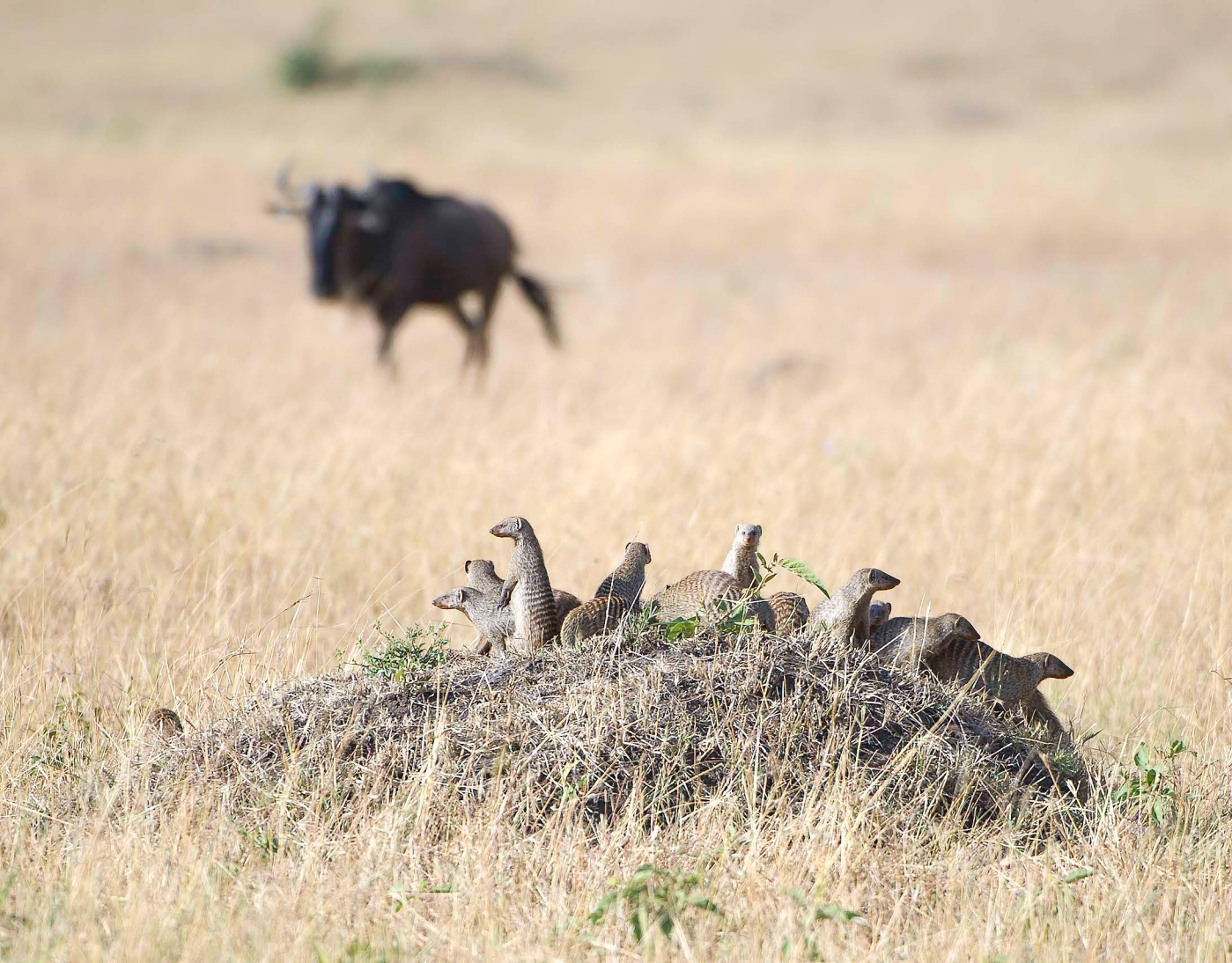

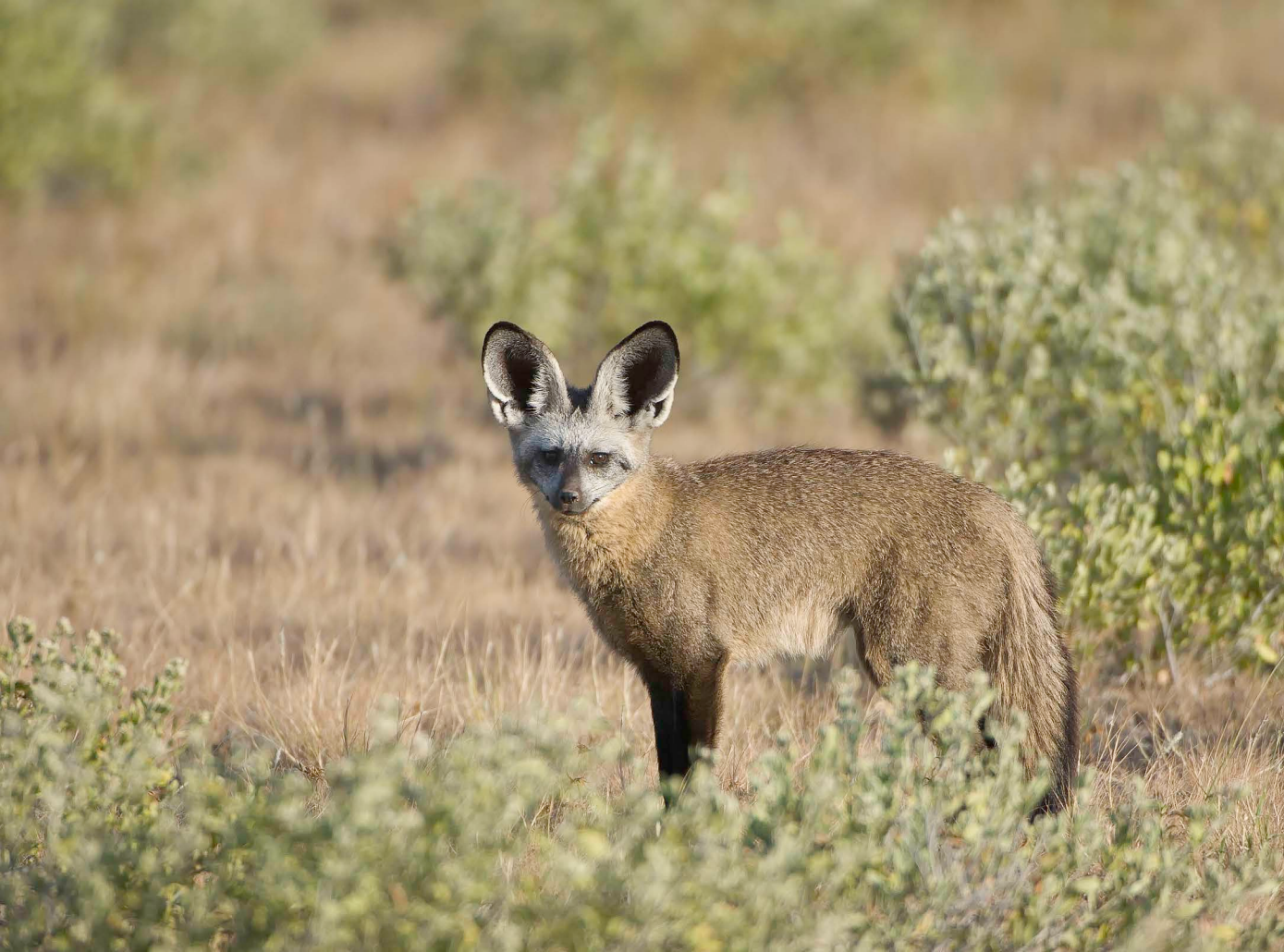

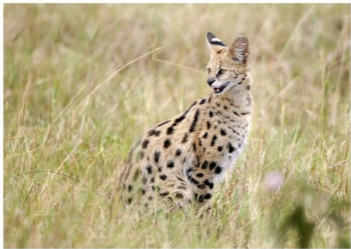

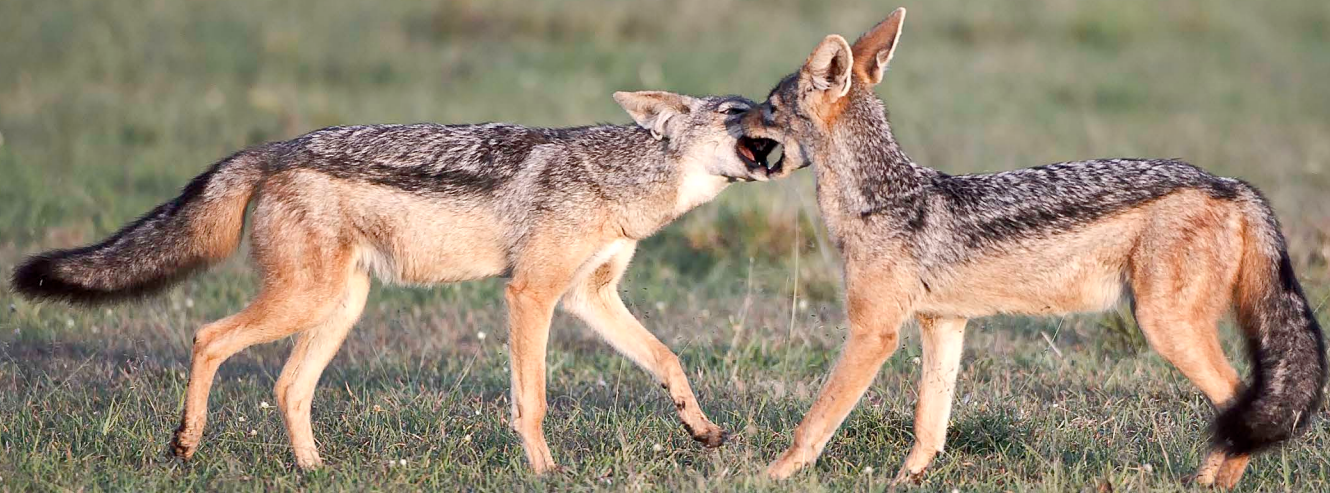

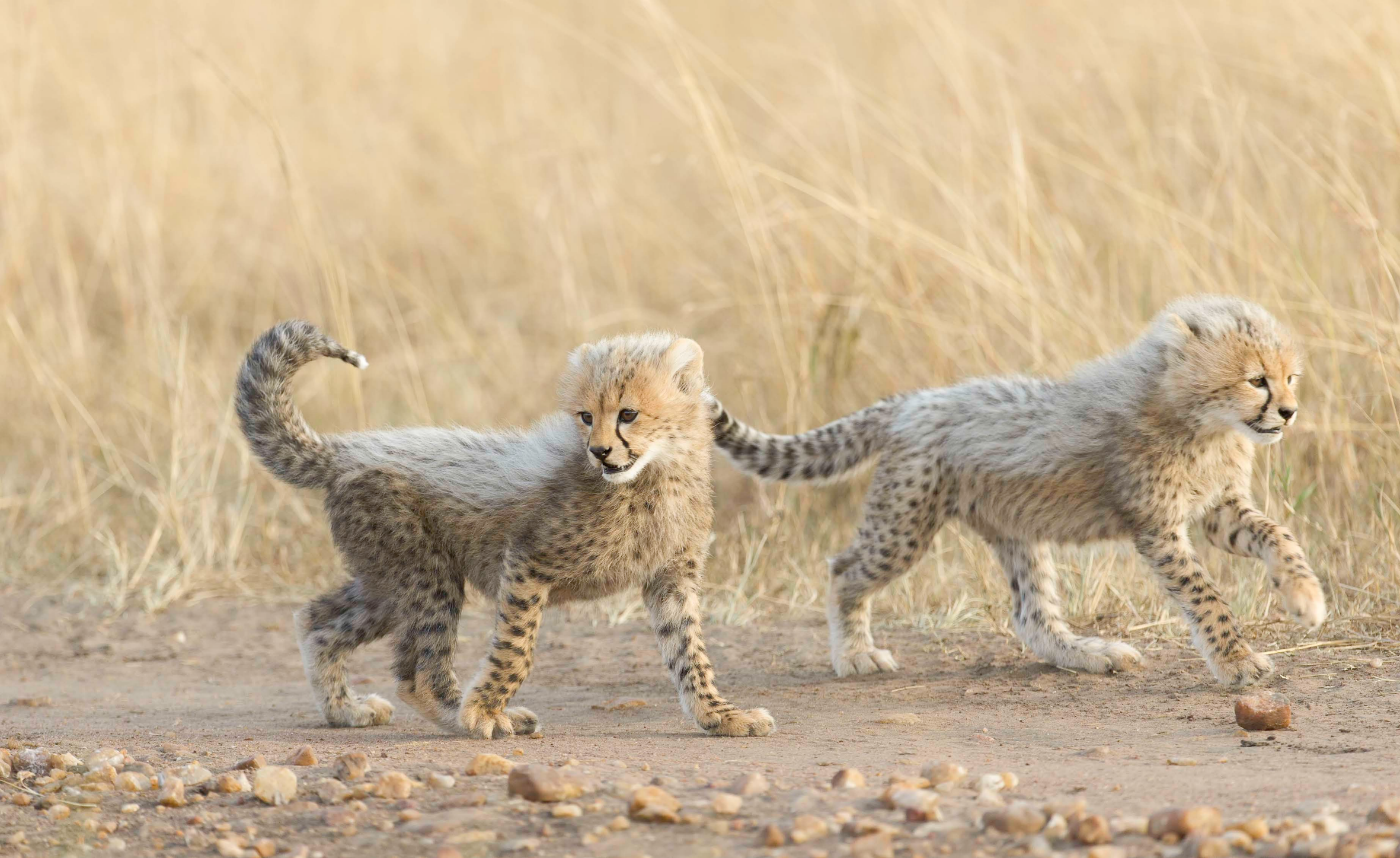

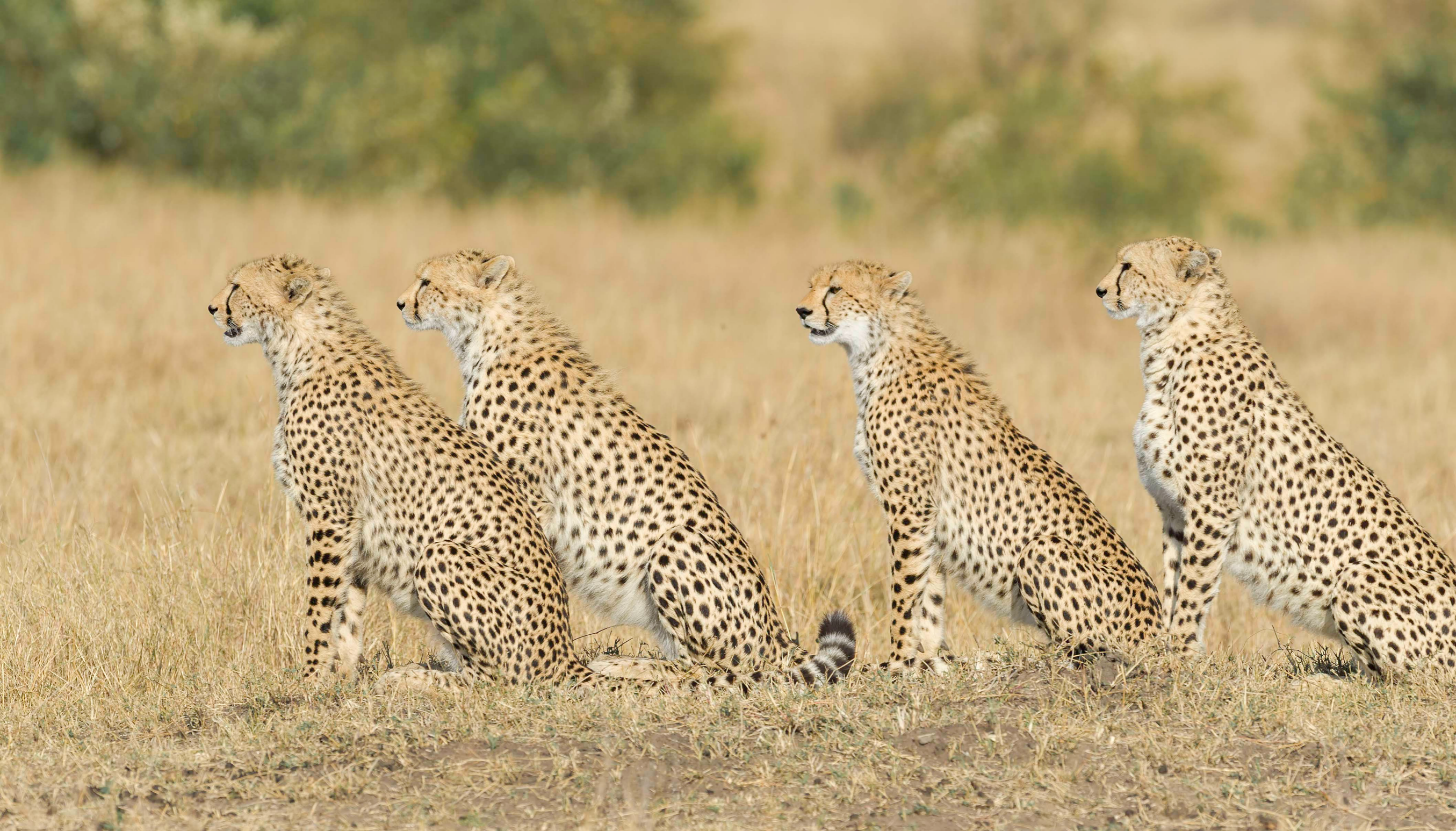

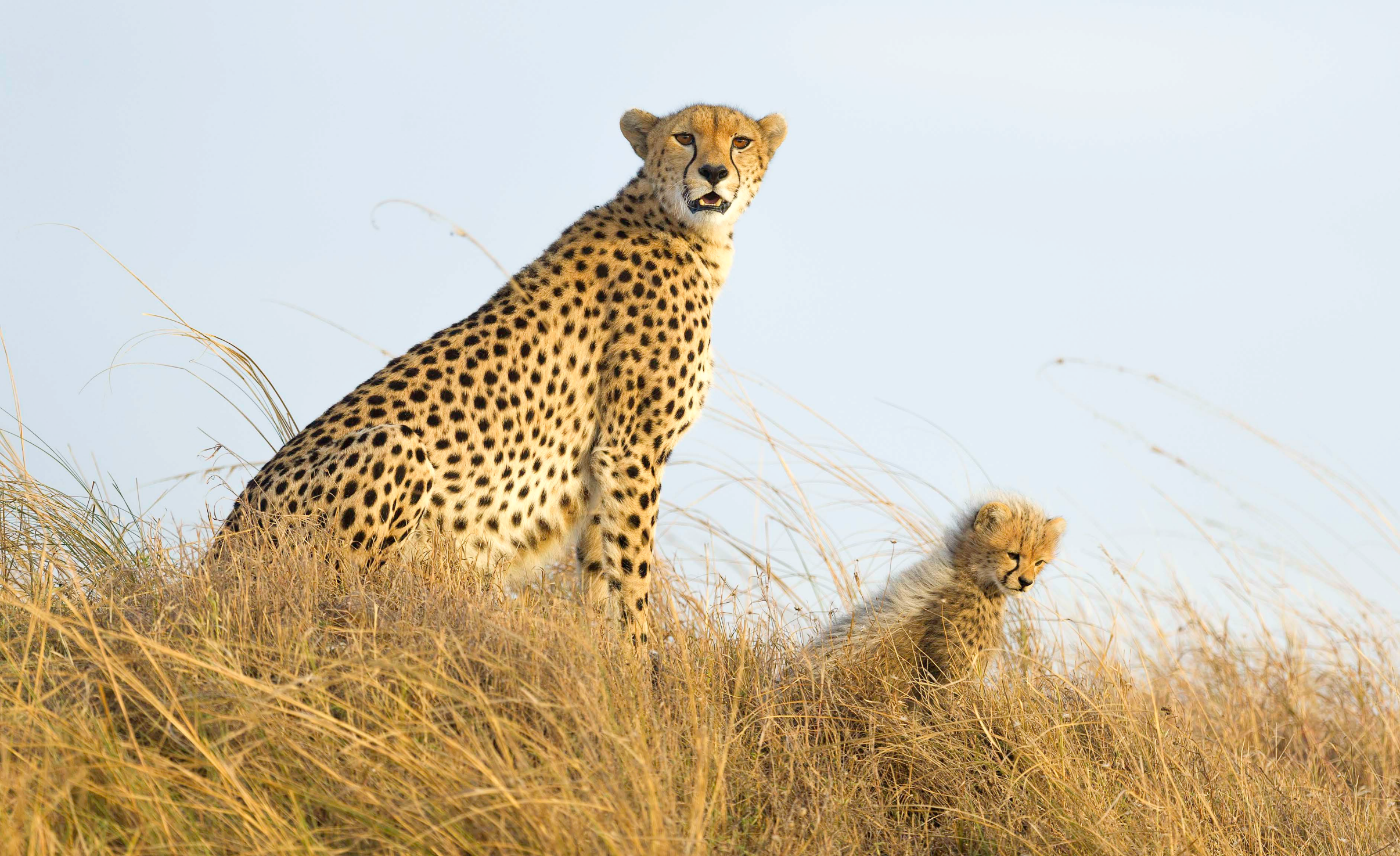

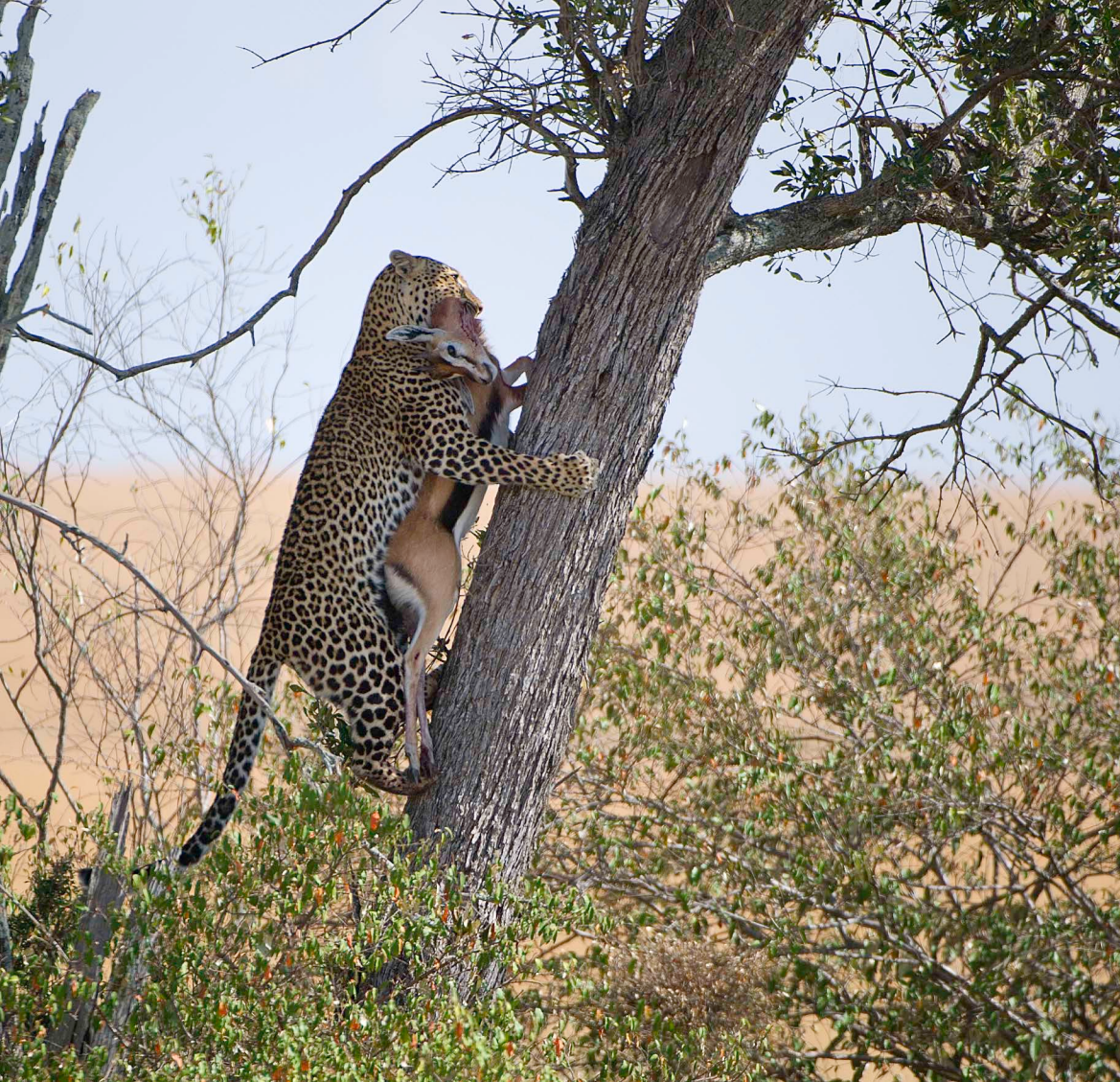

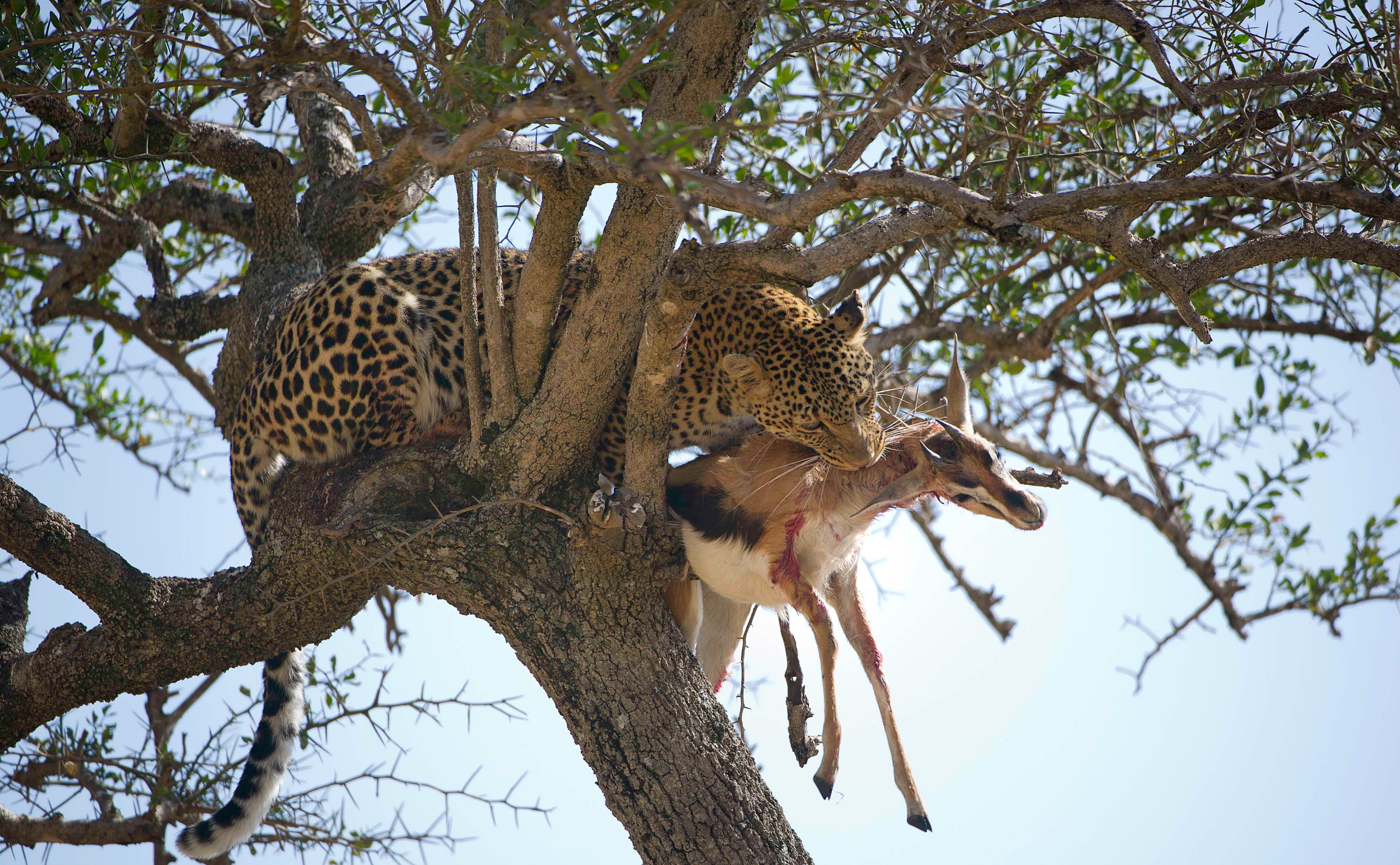

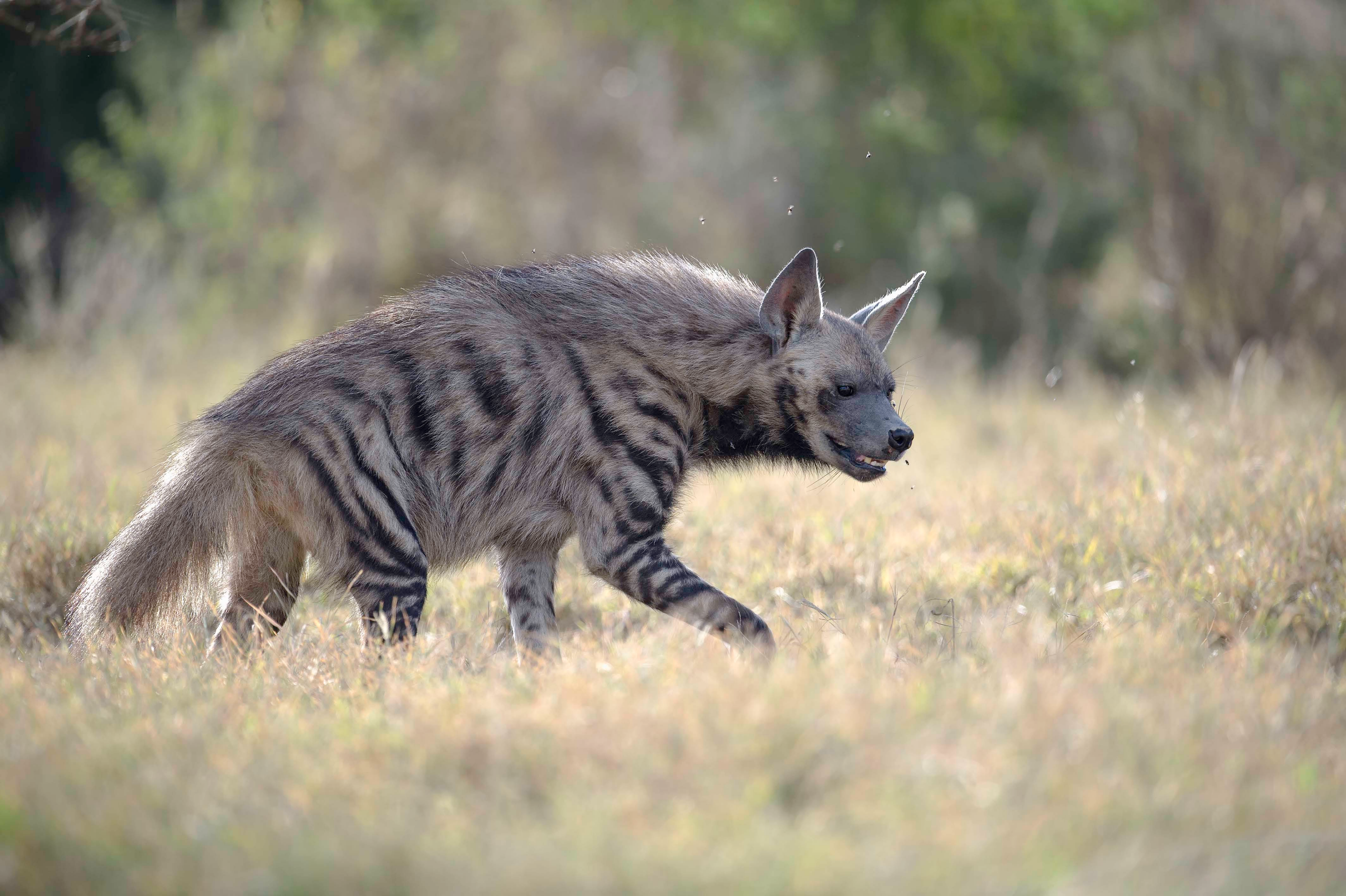

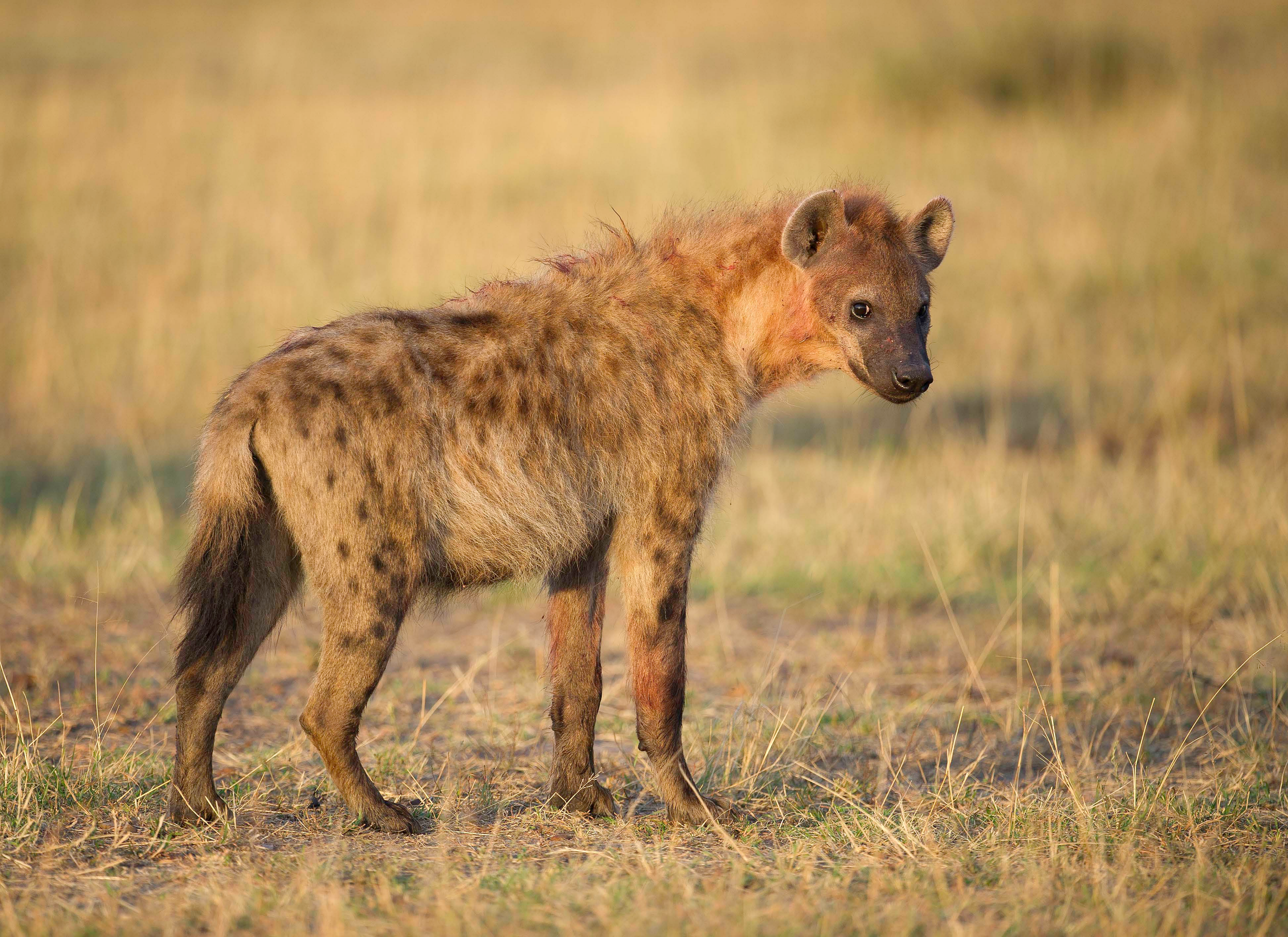

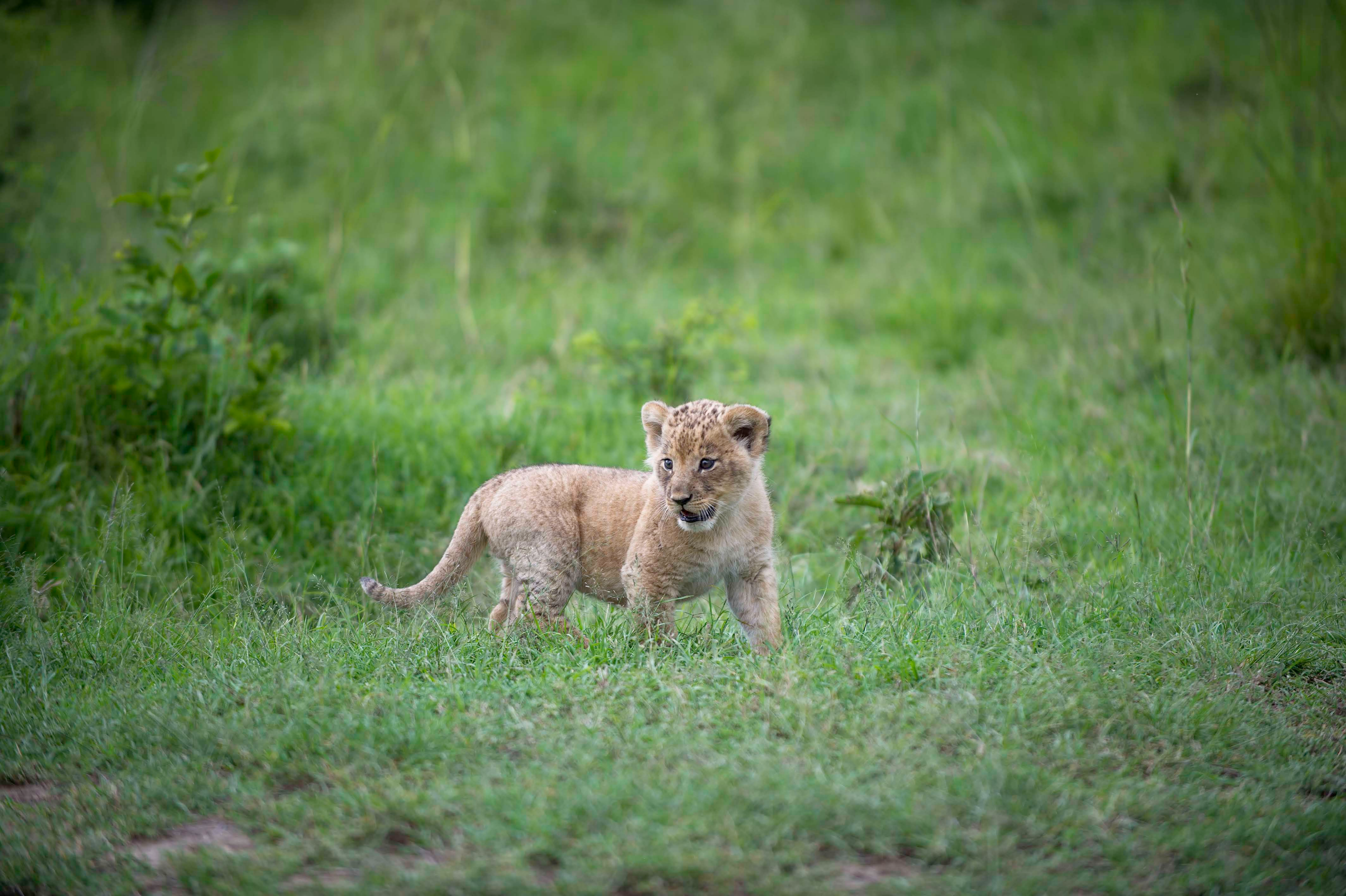

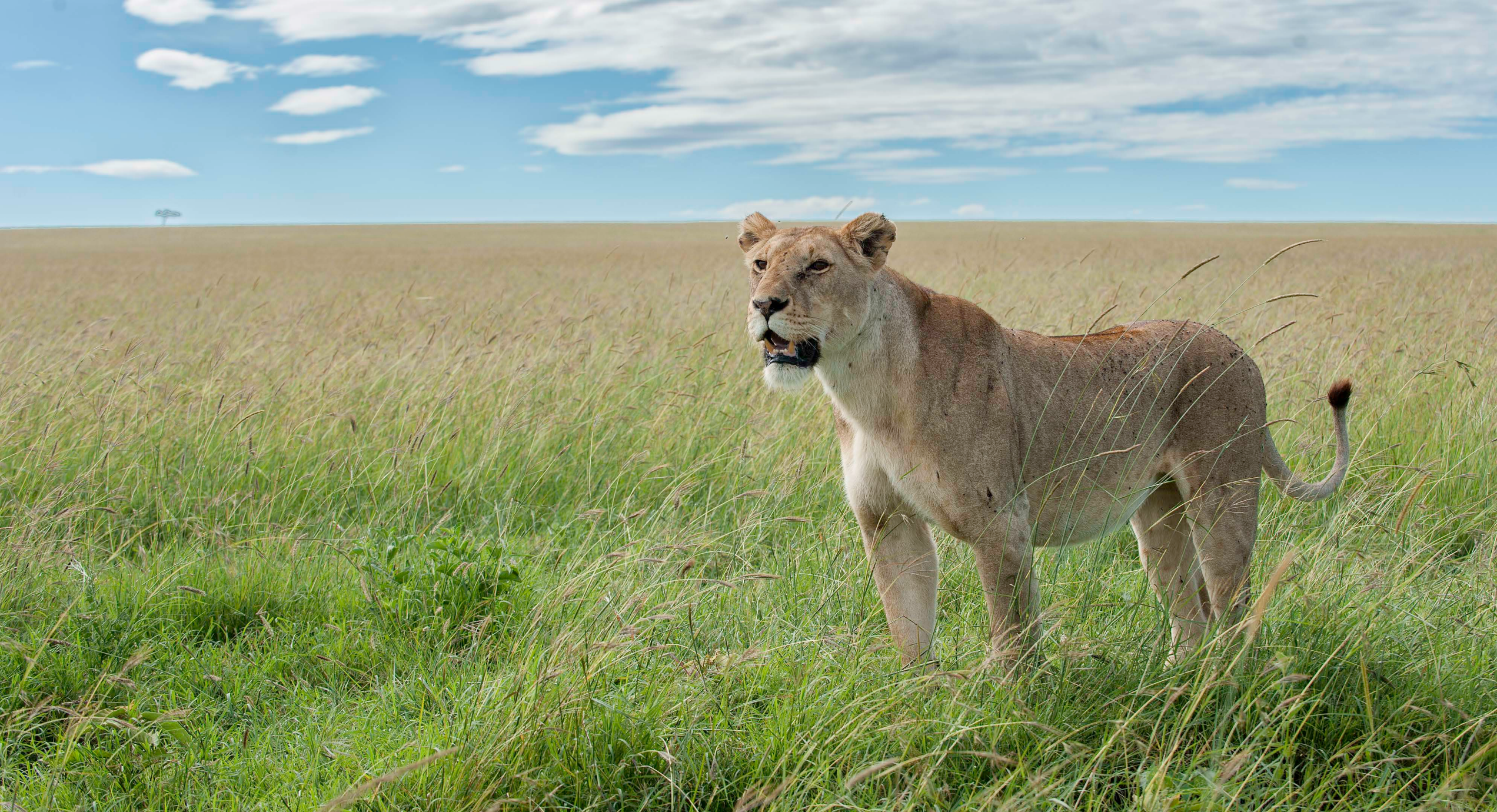

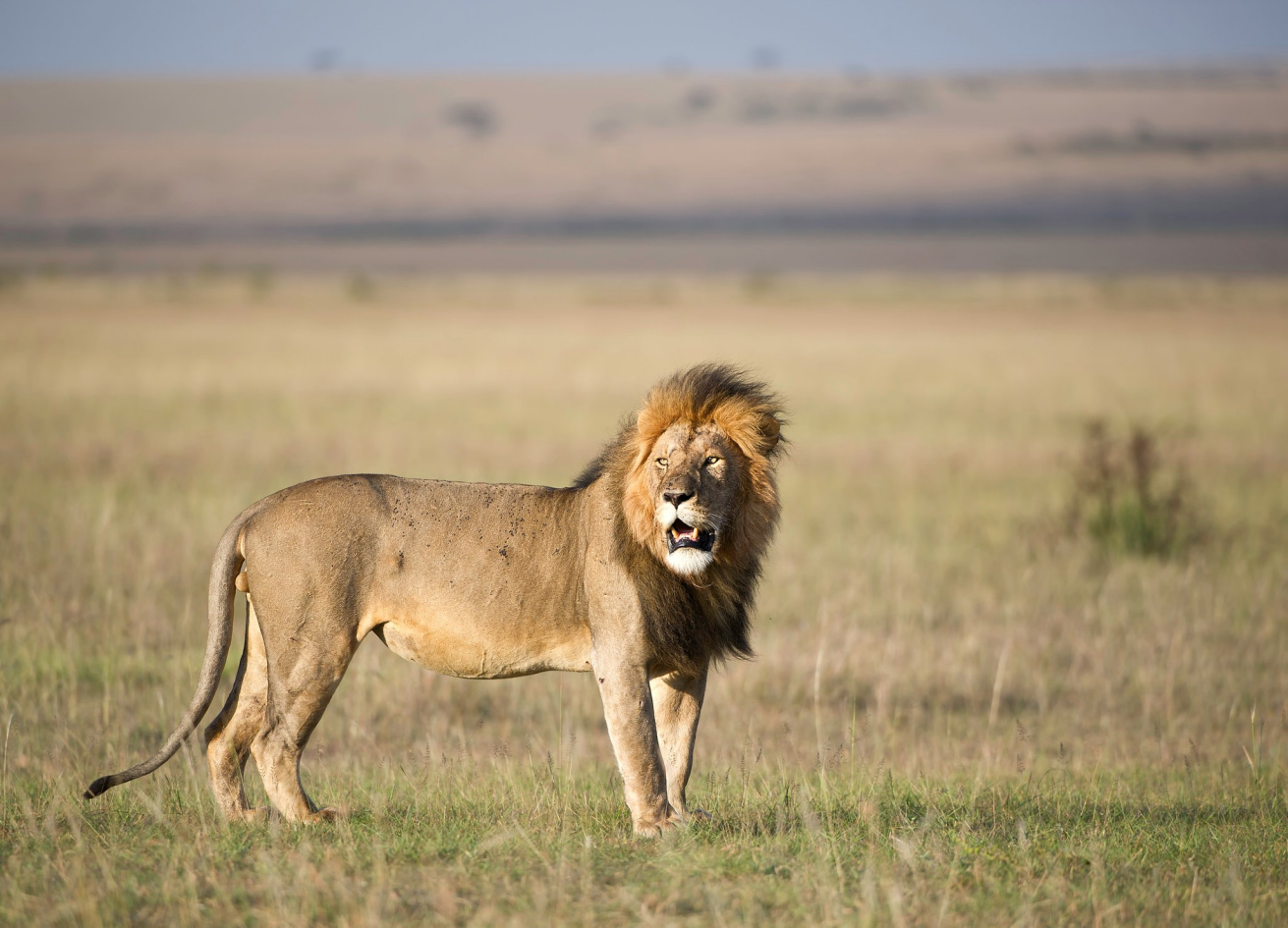

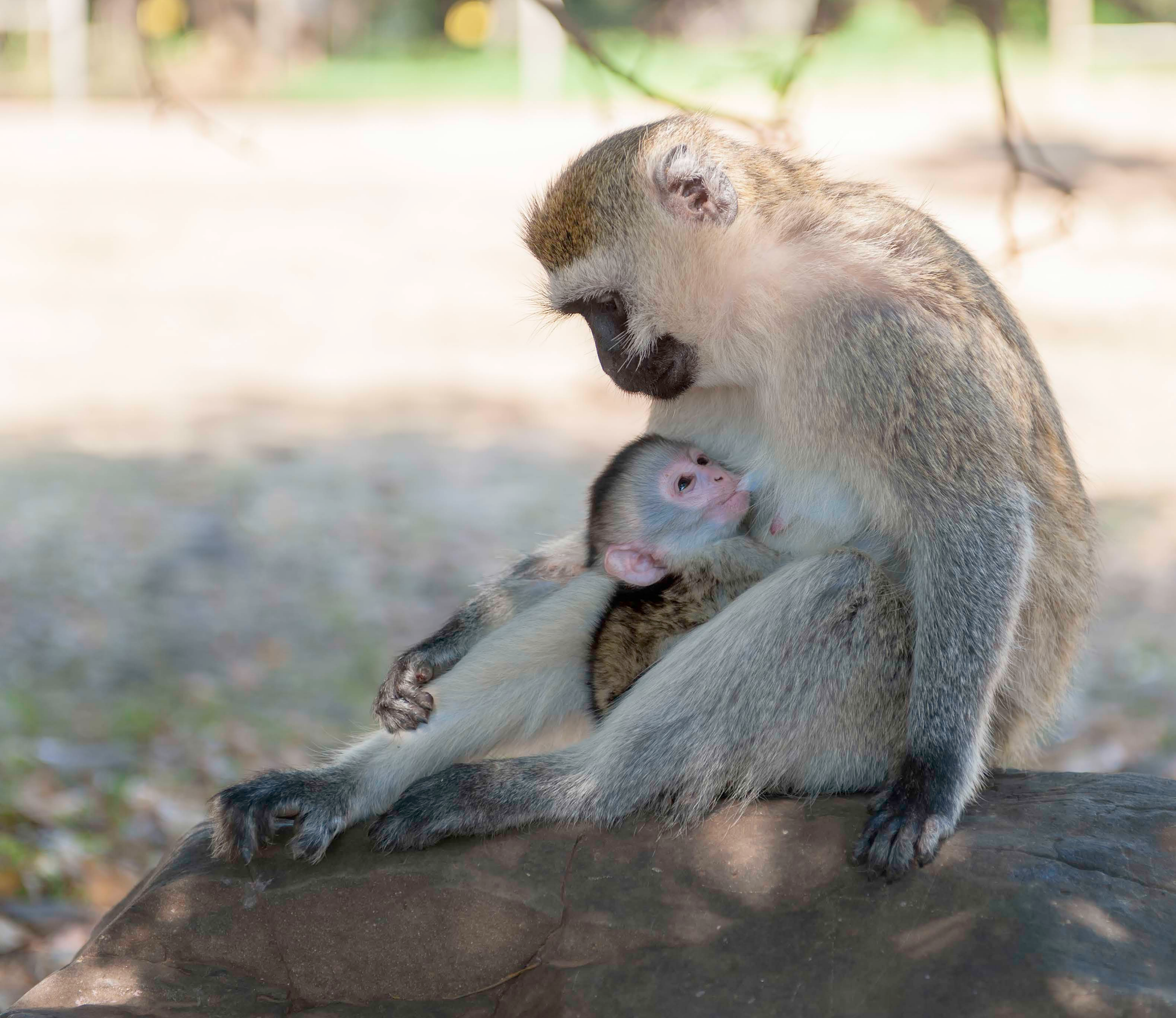

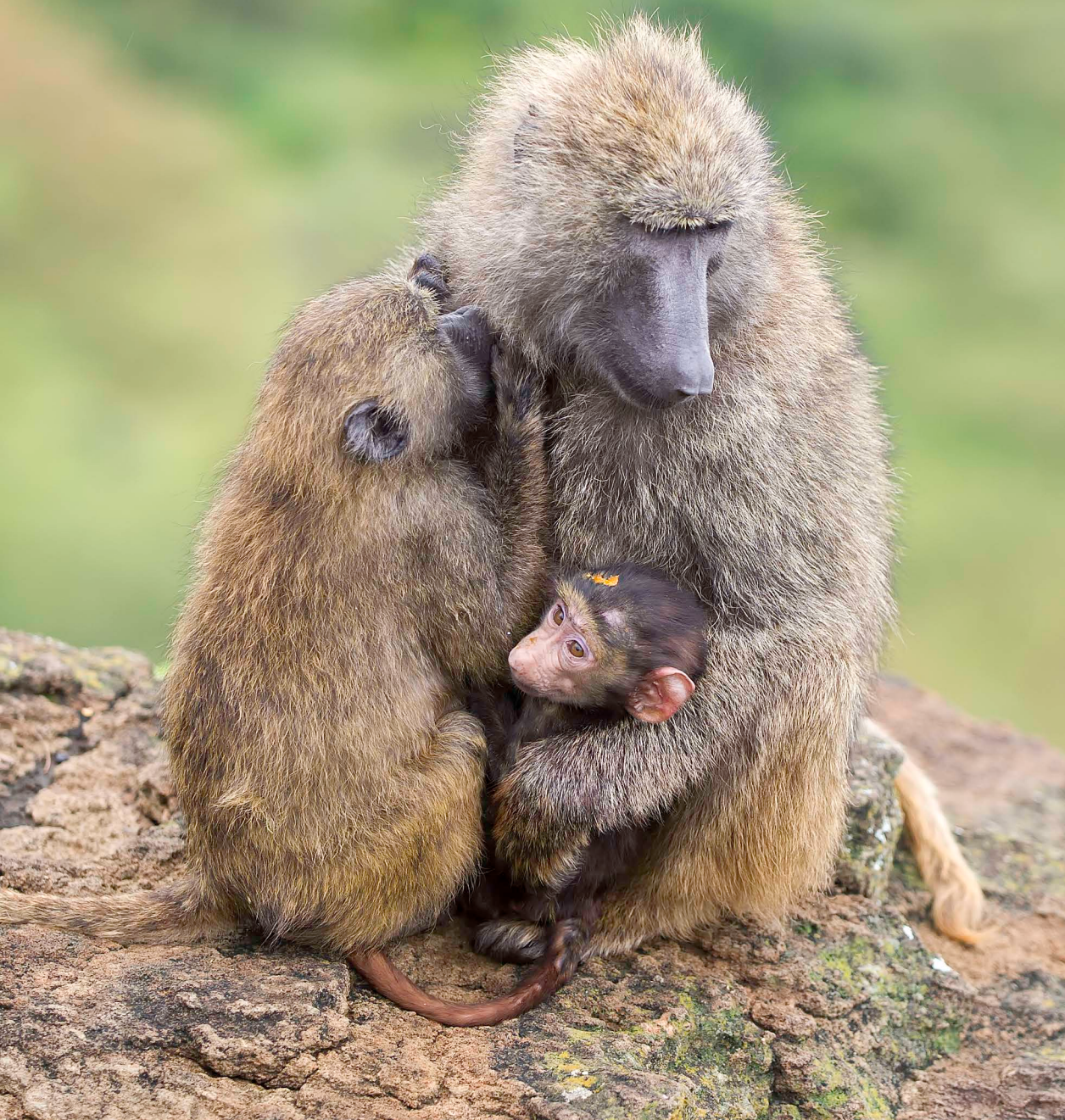

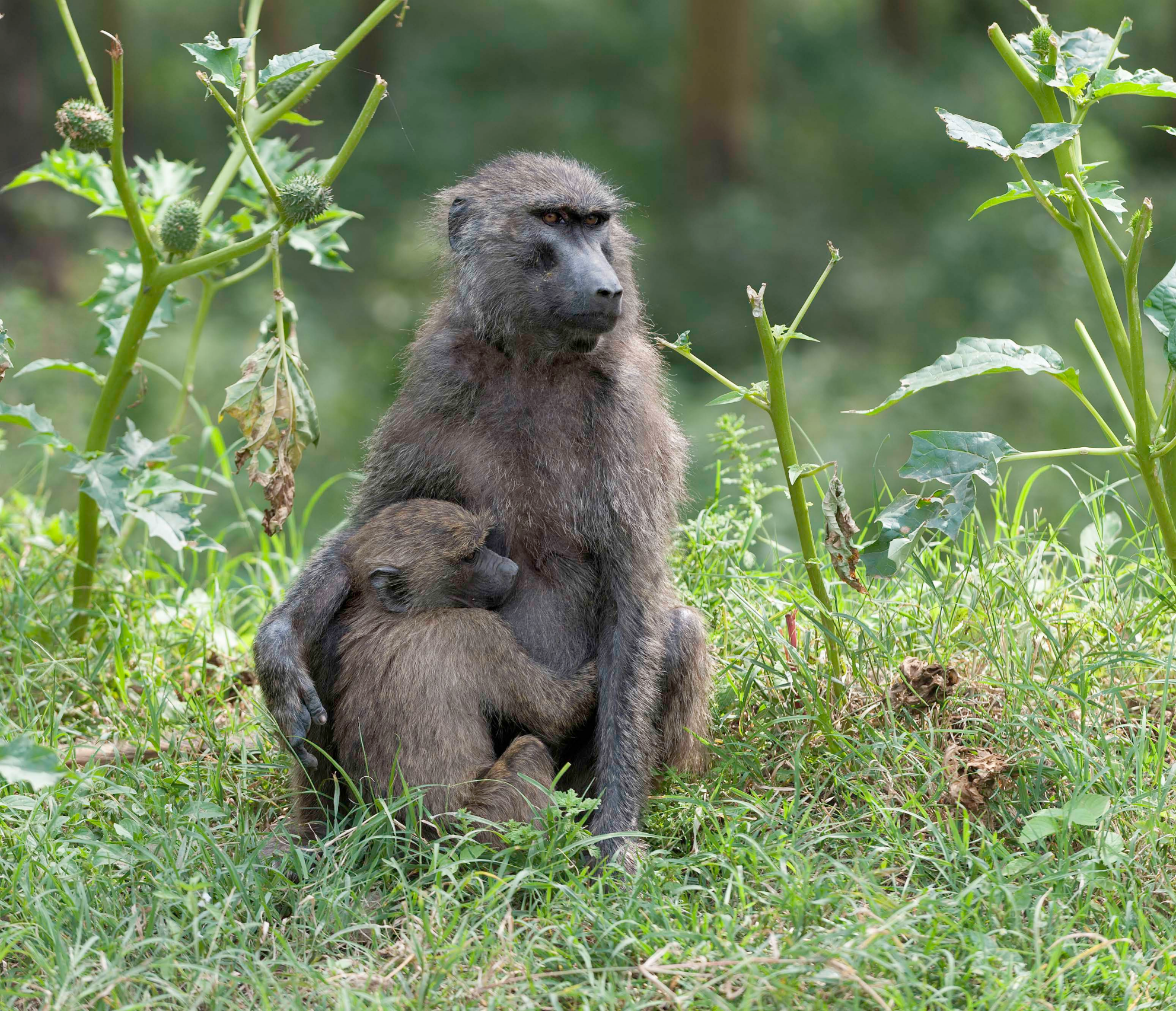

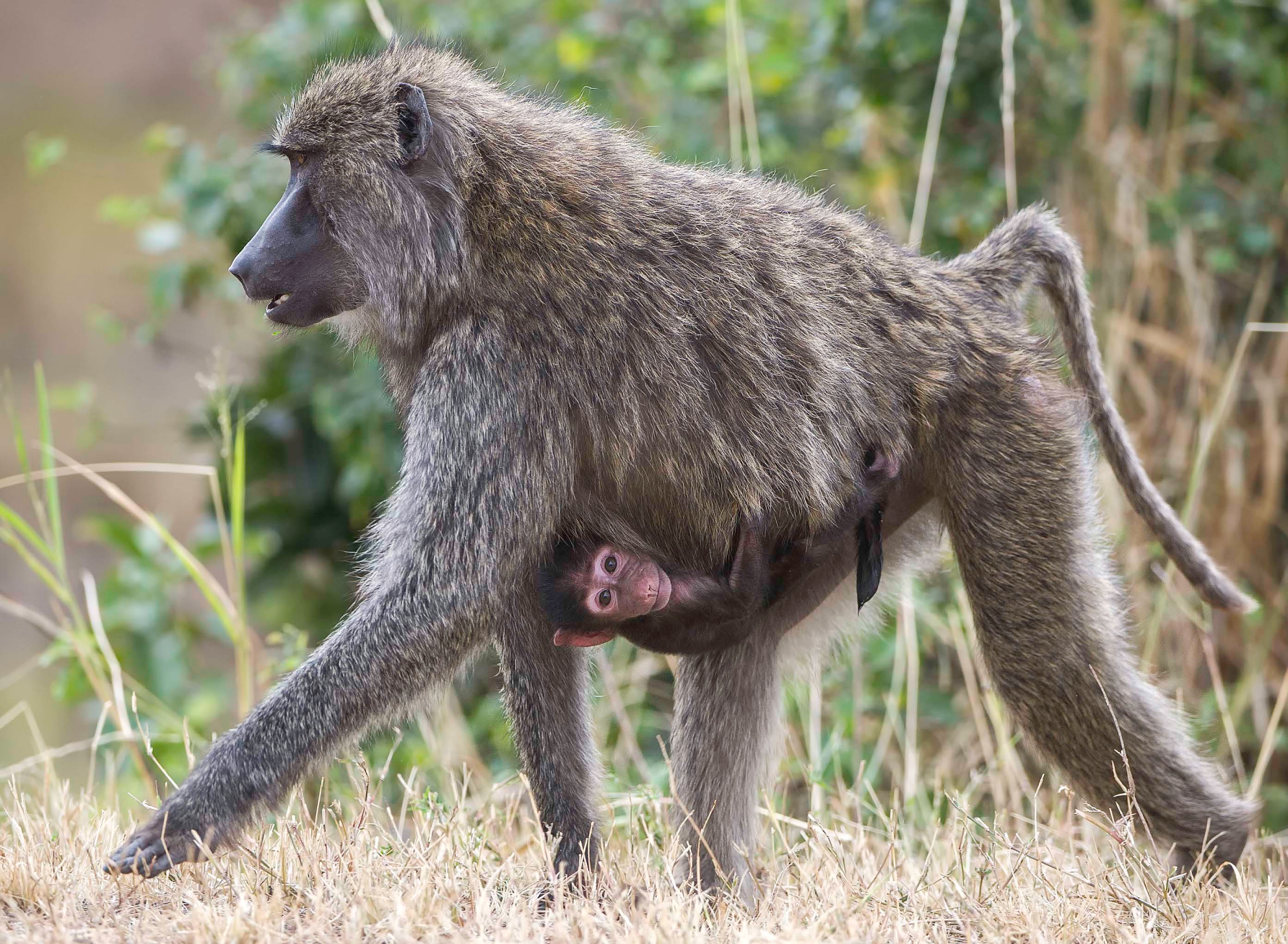

Supplement: S1 Fig — Photo Credit: Reto Buehler took all the photos except the photo of Thomson’s gazelle that was taken by Niels Mogensen. (PDF) [file pone.0169730.s005.pdf]

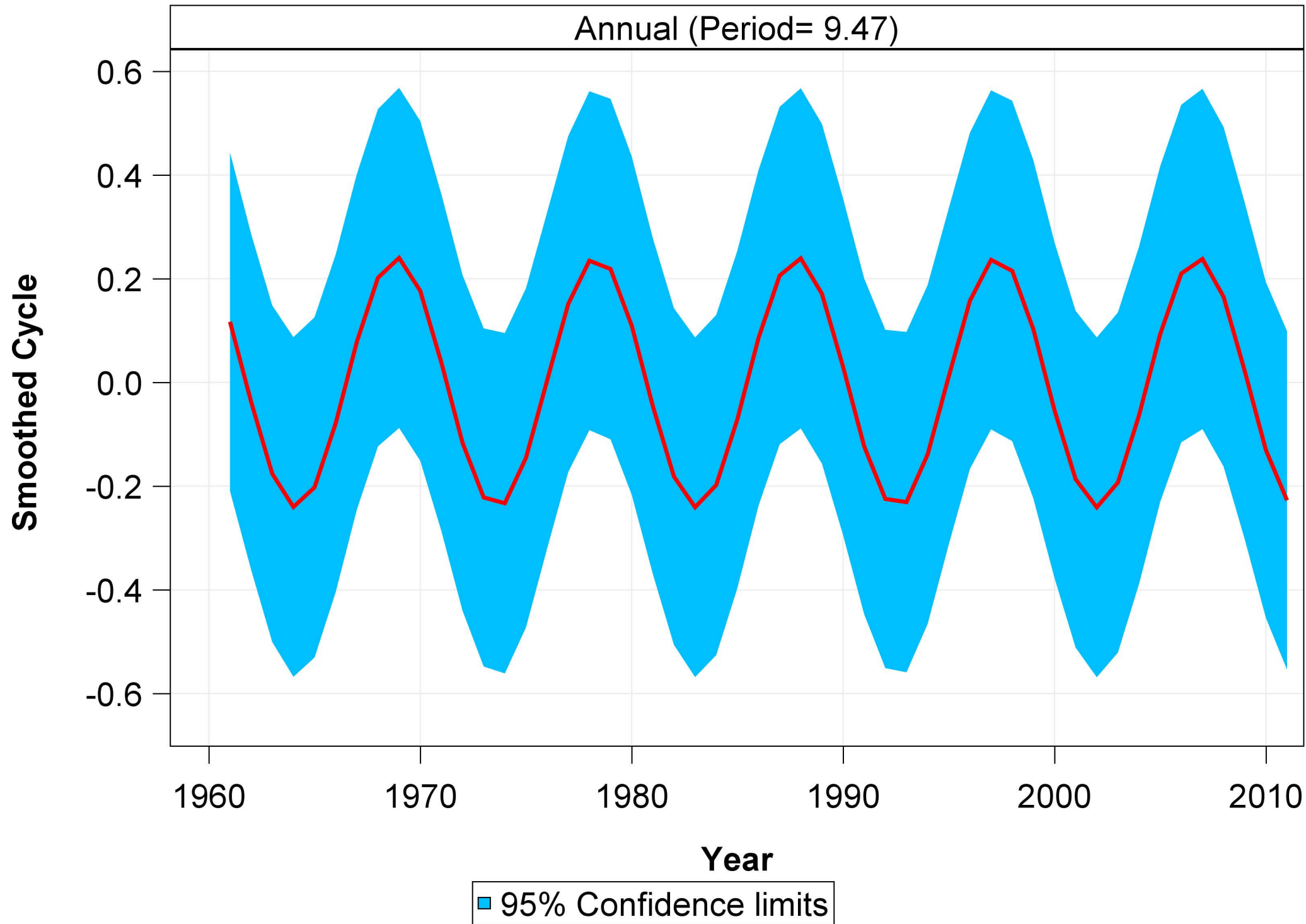

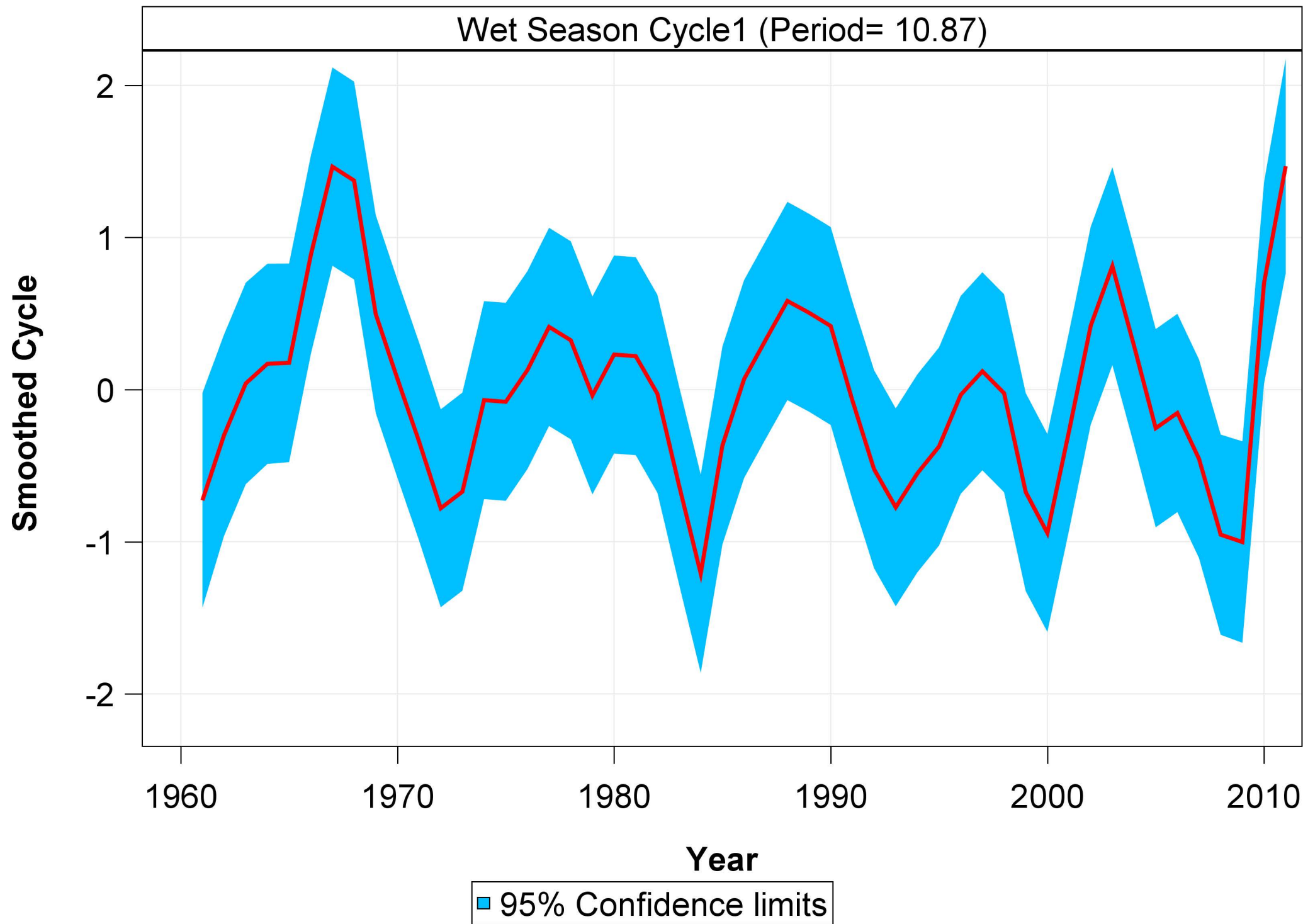

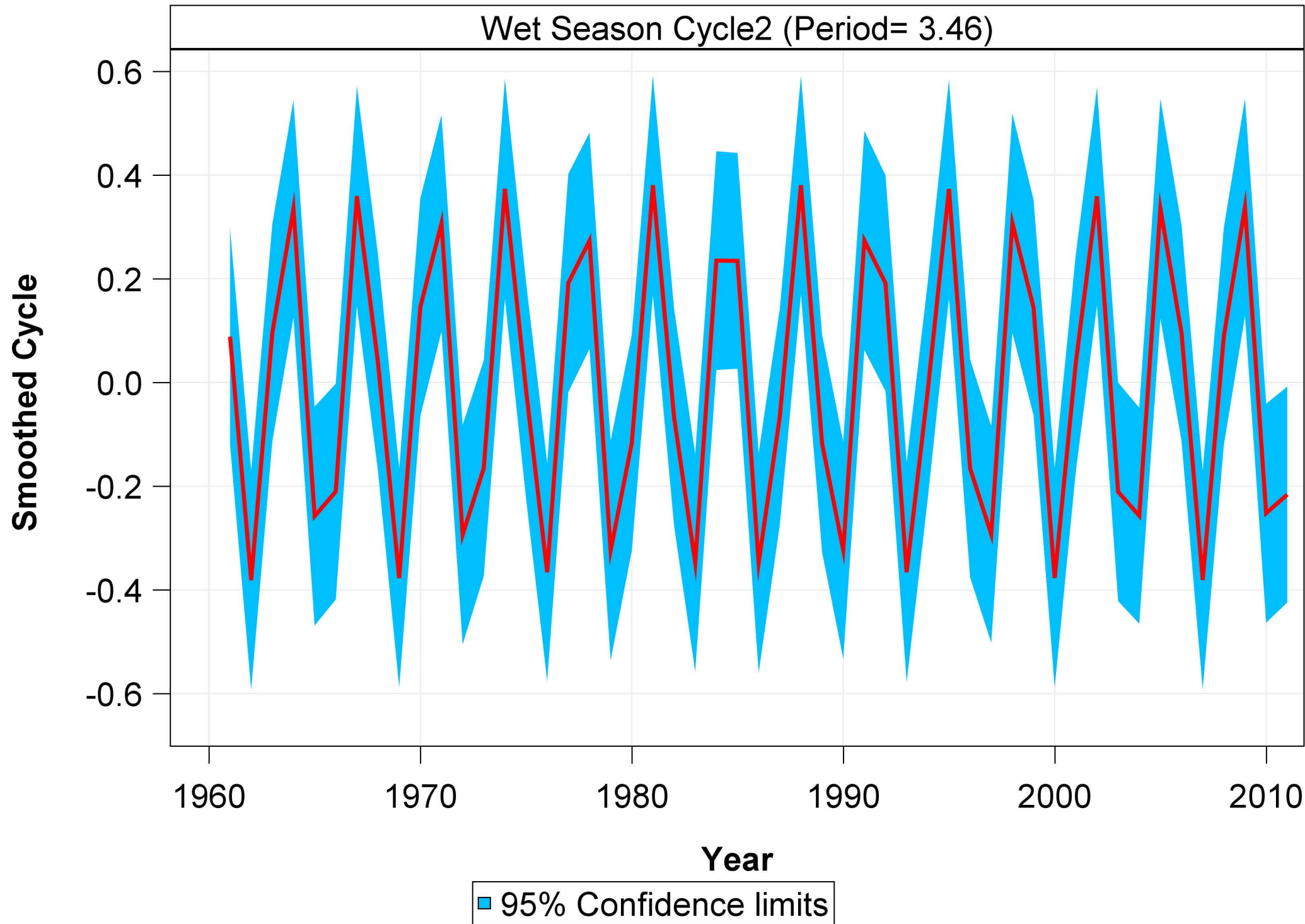

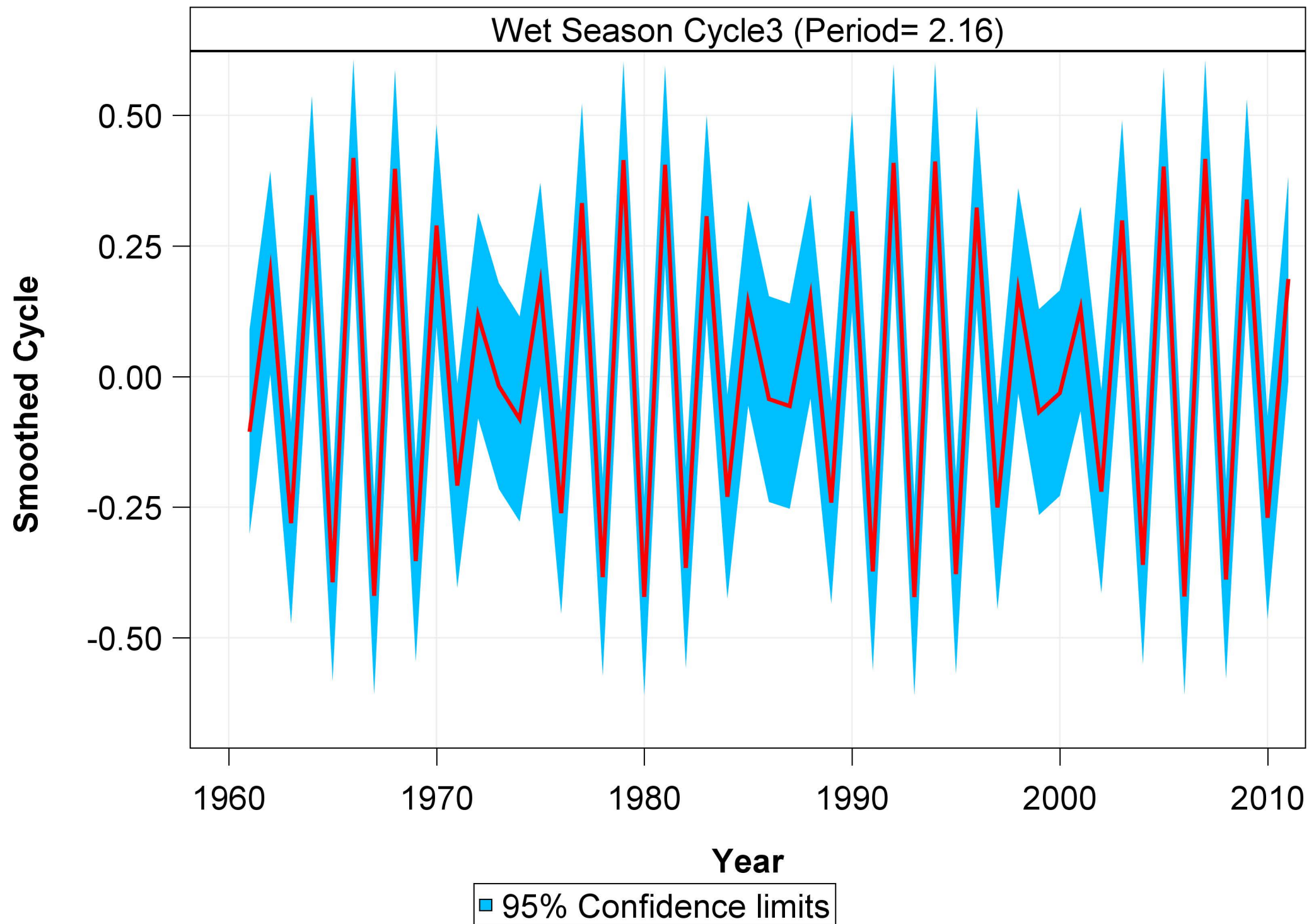

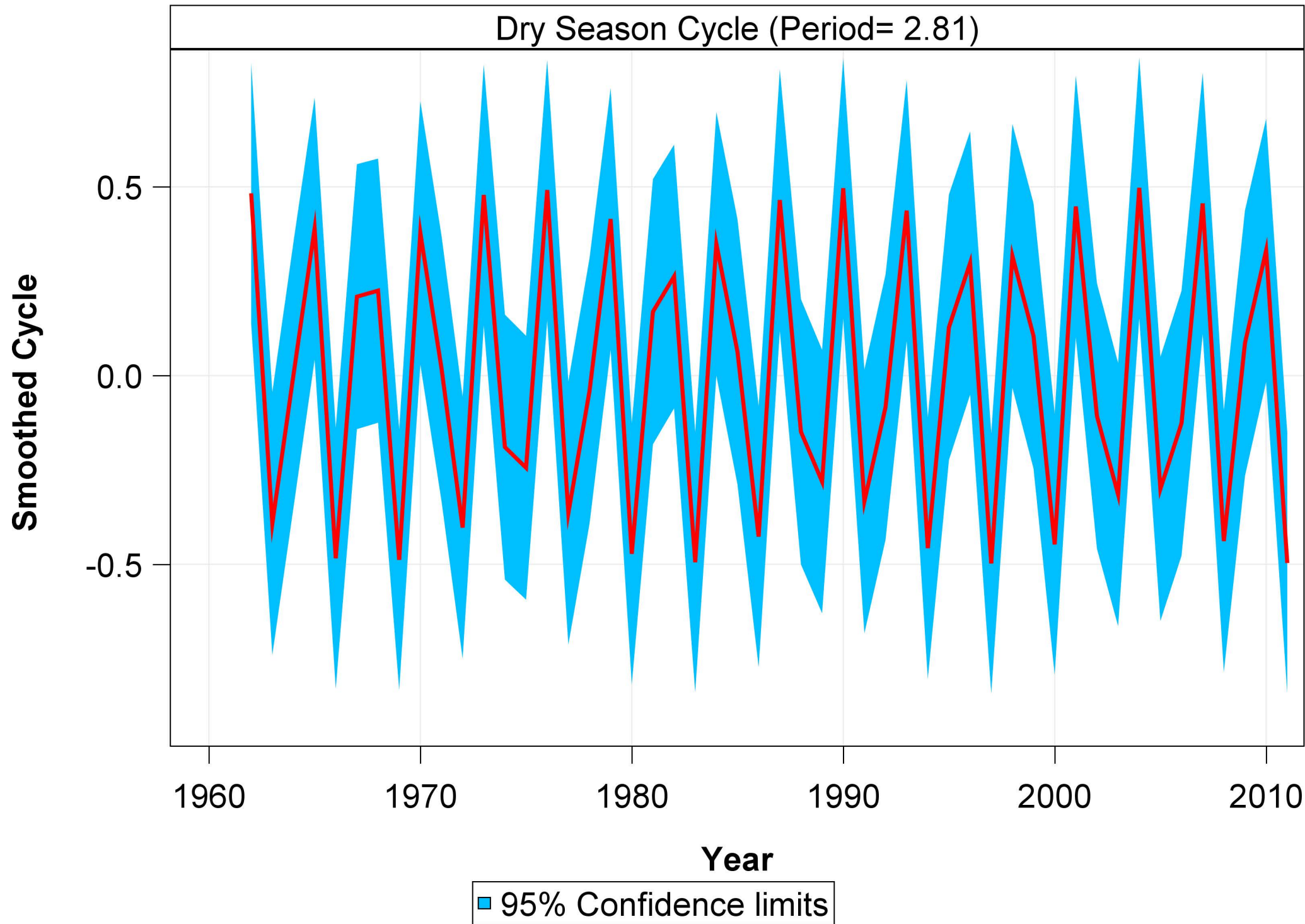

Supplement: S2 Fig — The shaded bands around the smoothed solid curves are the 95% confidence bands. (PDF) [file pone.0169730.s006.pdf]
