# Supplementary material for: Wildlife Population Dynamics in Human-Dominated Landscapes under Community-Based Conservation: The Example of Nakuru Wildlife Conservancy, Kenya
Source: PLoS One. 2017 Jan 19;12(1):e0169730. doi: 10.1371/journal.pone.0169730 (PMC5245813; doi:10.1371/journal.pone.0169730)
Supplement: S1 Table — Under dietary guild, O = Omnivore, M = mixed grazer/browser, G = pure grazer and B = pure browser. (DOCX) [file pone.0169730.s007.docx]

| Number | Common English name | Scientific name | Group | Dietary guild | Digestive physiology | Unit weight (in kg) | Mean Number/km^2^  (1996-2015) | Standard Deviation |
| --- | --- | --- | --- | --- | --- | --- | --- | --- |
| 1 | Helmeted Guinea fowl | *Numida meleagris* | Herbivore |  |  | 1 | 0.3214 | 0.2427 |
| 2 | Cape or brown hare | *Lepus capensis* | Herbivore |  | Non ruminant | 2 | 0.2289 | 0.1067 |
| 3 | African Spring hare | *Pedetes capensis* | Herbivore |  | Non ruminant | 3 | 0.0747 | 0.0638 |
| 4 | Rock hyrax | *Procavia capensis* | Herbivore | O |  | 4 | 0.2355 | 0.2077 |
| 5 | Dik dik | *Madoqua kirkii* | Herbivore | M | Ruminant | 7 | 0.5798 | 0.1502 |
| 6 | Steinbuck | *Raphicerus campestris* | Herbivore | M | Ruminant | 11 | 0.0552 | 0.0356 |
| 7 | Klipspringer | *Oreotragus oreotragus* | Herbivore | B | Ruminant | 14 | 0.0139 | 0.0086 |
| 8 | Thomson's gazelle | *Gazella thomsoni* | Herbivore | M | Ruminant | 15 | 10.0969 | 1.9877 |
| 9 | Oribi | *Ourebia ourebi* | Herbivore | M | Ruminant | 16 | 0.0433 | 0.0178 |
| 10 | Duiker | *Cephalophus silvicultor* | Herbivore | M | Ruminant | 20 | 0.0356 | 0.0174 |
| 11 | Porcupine | *Hystrix africaeaustralis* | Herbivore |  |  | 20 | 0.0130 | 0.0271 |
| 12 | Bohor reedbuck | *Redunca redunca* | Herbivore | G | Ruminant | 30 | 0.0708 | 0.0296 |
| 13 | Bushbuck | *Tragelaphus stricptus* | Herbivore | M | Ruminant | 30 | 0.0519 | 0.0415 |
| 14 | Impala | *Aepycerus melampus* | Herbivore | M | Ruminant | 40 | 6.8556 | 2.1201 |
| 15 | Grant's gazelle | *Gazella granti* | Herbivore | M | Ruminant | 40 | 1.5579 | 0.5209 |
| 16 | Warthog | *Pharcocoerus africanus* | Herbivore | M | Non ruminant | 45 | 1.5507 | 0.9189 |
| 17 | Aardvark | *Orycteropus afer* | Formivore |  |  | 53 | 0.0048 | 0.0049 |
| 18 | Topi | *Damaliscus lunatus korrigum* | Herbivore | G | Ruminant | 100 | 0.0585 | 0.0271 |
| 19 | Bushpig | *Potamochoerus larvatus* | Herbivore | O | Non ruminant | 103 | 0.0109 | 0.0040 |
| 20 | Masai ostrich | *Struthio camelus massaicus* | Omnivore | O |  | 114 | 0.0462 | 0.0399 |
| 21 | Wildebeest | *Connochaetes taurinus* | Herbivore | G | Ruminant | 120 | 0.5037 | 0.3234 |
| 22 | Hartebeest | *Alcelaphus busephalus cokei* | Herbivore | G | Ruminant | 125 | 1.2355 | 0.6830 |
| 23 | Oryx | *Oryx gazella* | Herbivore | G | Ruminant | 150 | 0.0256 | 0.0114 |
| 24 | Waterbuck | *Kobus ellipsiprymnus* | Herbivore | G | Ruminant | 160 | 0.9080 | 0.2675 |
| 25 | Burchell's zebra | *Equus quagga* | Herbivore | G | Non ruminant | 200 | 8.5587 | 1.6231 |
| 26 | Greater kudu | *Tragelaphus strepsiceros* | Herbivore | B | Ruminant | 223 | 0.0022 | 0.0021 |
| 27 | Eland | *Taurotragus oryx* | Herbivore | M | Ruminant | 350 | 1.6392 | 0.4269 |
| 28 | Grevy's zebra | *Equus grevyi* | Herbivore |  | Non ruminant | 400 | 0.0125 | 0.0056 |
| 29 | Buffalo | *Syncerus caffer* | Herbivore | G | Ruminant | 450 | 2.0435 | 0.7692 |
| 30 | Hippopotamus | *Hippopotamus amphibius* | Herbivore | G | Pseudoruminant | 1000 | 0.5105 | 0.4454 |
| 31 | White rhino | *Ceratotherium simum* | Herbivore | G | Non ruminant | 1000 | 0.0098 | 0.0043 |
| 32 | Giraffe | *Giraffa camelopardalis* *tippelskirchi* and *G.c. rothschildi* | Herbivore | B | Ruminant | 1250 | 0.4348 | 0.2128 |
| 33 | Sykes monkey | *Cercopithecus albogularis* | Omnivore |  |  | 6 | 0.0858 | 0.0499 |
| 34 | Vervet monkey | *Chlorocebus pygerythrus* | Omnivore |  |  | 6 | 0.3084 | 0.1513 |
| 35 | Colobus monkey | *Colobus guereza* | Herbivore |  |  | 15 | 0.0828 | 0.0372 |
| 36 | Anubis baboon | *Papio anubis* | Omnivore |  |  | 20 | 0.9421 | 0.3117 |
| 37 | White tailed Mongoose | *Ichneumia albicauda* | Carnivore |  |  | 3 | 0.0322 | 0.0502 |
| 38 | Bat-eared fox | *Otocyon megalotis* | Carnivore |  |  | 5 | 0.0553 | 0.0849 |
| 39 | Serval cat | *Leptailurus serval* | Carnivore |  |  | 10 | 0.0090 | 0.0072 |
| 40 | Golden jackal | *Canis aureus* | Carnivore |  |  | 15 | 0.1377 | 0.0485 |
| 41 | Cheetah | *Acinonyx jubatus* | Carnivore |  |  | 45 | 0.0033 | 0.0026 |
| 42 | Leopard | *Panthera pardus* | Carnivore |  |  | 45 | 0.0097 | 0.0035 |
| 43 | Spotted hyena | *Crocuat crocucta* | Carnivore |  |  | 45 | 0.0353 | 0.0187 |
| 44 | Lion | *Panthera leo* | Carnivore |  |  | 80 | 0.0010 | 0.0027 |
