# Supplementary material for: Wildlife Population Dynamics in Human-Dominated Landscapes under Community-Based Conservation: The Example of Nakuru Wildlife Conservancy, Kenya
Source: PLoS One. 2017 Jan 19;12(1):e0169730. doi: 10.1371/journal.pone.0169730 (PMC5245813; doi:10.1371/journal.pone.0169730)
Supplement: S2 Table — (DOCX) [file pone.0169730.s008.docx]

| Number | Name of National Park, Private Conservancy or Ranch | Acres | Km^2^ |
| --- | --- | --- | --- |
| 1 | Archer Smith | 40 | 0.161874 |
| 2 | Kilima Ndege | 88 | 0.356124 |
| 3 | Bushy Island | 100 | 0.404686 |
| 4 | Crescent Island | 190 | 0.768903 |
| 5 | DDD (Domino De Doriano), later renamed ROCCO farm | 400 | 1.61874 |
| 6 | Shangrila | 500 | 2.02343 |
| 7 | Ol Olerai | 500 | 2.02343 |
| 8 | Nderit | 500 | 2.02343 |
| 9 | Kongoni Game Valley | 800 | 3.237488 |
| 10 | Mundui | 1150 | 4.653889 |
| 11 | Kenya Wildlife Service Institute (KWSTI) Annex (200 acres), (Mirera, Mararo, Karagita: Small farms around Kenya Wildlife Service Training Institute (KWSTI), later subdivided and settled by flower farmers) | 1460 | 5.908416 |
| 12 | Green Park Brixia Limited | 1500 | 6.07029 |
| 13 | Manera | 1600 | 6.474976 |
| 14 | Morendat | 1700 | 6.87966 |
| 15 | Ol Arag | 1800 | 7.284348 |
| 16 | Oserian Game Corridor (links Kedong, Hell’s Gate and Longonot to Lake Naivasha) | 3000 | 12.14058 |
| 17 | Kigio | 3500 | 14.16401 |
| 18 | Mbo I Kamiti | 4500 | 18.21087 |
| 19 | Malu | 5000 | 20.2343 |
| 20 | Loldia farms | 6000 | 24.28116 |
| 21 | KARI (6000 acres) + Ol Magogo (3000 acres) | 9000 | 36.421708 |
| 22 | Crater Lake | 10000 | 40.4686 |
| 23 | Nyondia | 10000 | 40.4686 |
| 24 | Hell’s Gate National Park | 16000 | 64.74976 |
| 25 | Kongoni Wildlife Sanctuary, later renamed Oserian Wildlife Conservancy | 18000 | 72.84348 |
| 26 | Longonot National Park | 20000 | 80.9372 |
| 27 | Kedong Ranch, Longonot Section | 30000 | 121.4058 |
| 28 | Marula | 30000 | 121.4058 |
| 29 | Ndabibi | 40000 | 161.8744 |
| 30 | Kekopey Group Ranch | 44000 | 178.0618 |
| 31 | Soysambu | 56000 | 226.6242 |
| 32 | Kedong Ranch, Akira Section | 77000 | 311.6082 |
| 33 | Kedong Ranch (until April 1999): Longonot and Akira sections combined | 80000 | 323.7488 |
