# Supplementary material for: Wildlife Population Dynamics in Human-Dominated Landscapes under Community-Based Conservation: The Example of Nakuru Wildlife Conservancy, Kenya
Source: PLoS One. 2017 Jan 19;12(1):e0169730. doi: 10.1371/journal.pone.0169730 (PMC5245813; doi:10.1371/journal.pone.0169730)
Supplement: S1 File — (DOCX) [file pone.0169730.s011.docx]

**Supplementary Materials S1 File.**

/*------Modeling trend and cycles in the wet season rainfall data using the Unobserved Components Model (UCM) in SAS (Version 9.4) UCM procedure--*/;

ods graphics on;

ods output SmoothedCycle=wet_SmoothedCycle outliersummary=wet_outliersummary ParameterEstimates=wet_ParameterEstimates

ComponentSignificance=wet_ComponentSignificance;

**proc** **ucm** data=Naivasha_rain4_std;

*id year interval=year;

model wet;

irregular;

level var=**0** noest;

cycle plot=(filter smooth) ;

cycle plot=(filter smooth) ;

cycle plot=(filter smooth) ;

estimate back=**10** plot=(loess panel cusum wn);

*forecast back=10 lead=10 plot=(forecasts decomp);

**run**;

ods graphics off;

/*--SAS (Version 9.4) code used to simultaneously model the population trends for the 44 wildlife species.*/

ods output covparms=covparms parameterestimates=Parmestimates;

**Proc** **glimmix** data=Naivasha10 method=RSPL initglm hessian asycov initglm outdesign(names)=XZmat;

class species;

Model count=species species*date /noint dist=negbin link=log ddfm=kr offset=logarea ;

Random date /sub=intercept type=pspline knotmethod=equal(**20**)V G solution ;

Random date /sub=species type=pspline knotmethod=equal(**20**) V G solution;

parms (**0.000000**) (**9.735E-6**)( **0.2795**);

output out=_Pred2 Pred(ilink)=mu lcl(ilink)=lower ucl(ilink)=upper;

nloptions tech=NRRIDG Maxiter=**1000** maxfunc=**5000**;

**run**;
